# The Effect of Daily Self-Measurement of Pressure Pain Sensitivity Followed by Acupressure on Depression and Quality of Life versus Treatment as Usual in Ischemic Heart Disease: A Randomized Clinical Trial

**DOI:** 10.1371/journal.pone.0097553

**Published:** 2014-05-21

**Authors:** Natasha Bergmann, Søren Ballegaard, Per Bech, Åke Hjalmarson, Jesper Krogh, Finn Gyntelberg, Jens Faber

**Affiliations:** 1 Herlev University Hospital, Department of Endocrinology, Herlev, Denmark; 2 Ull Care A/S, Hellerup, Denmark; 3 Psychiatric Research Unit, Psychiatric Centre North Zealand, Hillerød, Denmark; 4 Department of Cardiology, Sahlgrenska University Hospital, Gothenburg, Sweden; 5 The National Research Centre for the Working Environment, Copenhagen, Denmark; 6 Faculty of Health Sciences, Copenhagen University, Copenhagen, Denmark; University of Michigan, United States of America

## Abstract

**Background:**

Depressive symptoms and reduced quality of life (QOL) are parts of the chronic stress syndrome and predictive of adverse outcome in patients with ischemic heart disease (IHD). Chronic stress is associated with increased sensitivity for pain, which can be measured by algometry as Pressure Pain Sensitivity (PPS) on the sternum.

**Aim:**

To evaluate if stress focus by self-measurement of PPS, followed by stress reducing actions including acupressure, can decrease depressive symptoms and increase psychological well-being in people with stable IHD.

**Design:**

Observer blinded randomized clinical trial over 3 months of either intervention or treatment as usual (TAU). Statistical analysis: Intention to treat.

**Methods:**

Two hundred and thirteen participants with IHD were included: 106 to active treatment and 107 to TAU. Drop-out: 20 and 12, respectively. The active intervention included self-measurement of PPS twice daily followed by acupressure as mandatory action, aiming at a reduction in PPS. *Primary endpoint:* change in depressive symptoms as measured by Major depression inventory (MDI). *Other endpoints*: changes in PPS, Well-being (WHO-5) and mental and physical QOL (SF-36).

**Results:**

At 3 months PPS decreased 28%, to 58, in active and 11%, to 72, in TAU, p<0.001. MDI decreased 22%, to 6.5, in active group vs. 12%, to 8.3 in TAU, p = 0.040. WHO-5 increased to 71.0 and 64.8, active group and TAU, p = 0.015. SF-36 mental score sum increased to 55.3 and 53.3, active and TAU, p = 0.08.

**Conclusions:**

PPS measurements followed by acupressure reduce PPS, depressive symptoms and increase QOL in patients with stable IHD.

**Trial Registration:**

ClinicalTrials.gov NCT01513824

## Introduction

The bidirectional interaction between depression and ischemic heart disease (IHD) has been documented numerous times and is generally accepted [1]. After an acute myocardial infarction (MI) the risk of being depressed is approximately 3 times increased as compared with the general population [2]. In out-patients the 12-month odds ratio of major depression has been found to be 2.3 times higher in individuals with cardiac disease as compared with those with no medical illness [3]. In initially healthy people clinical depression as well as depressive mood is associated with a significantly increased risk of developing IHD [4]. Further, depression after a MI doubles both the risk of a cardiac re-event during the first 2 years post MI, as well the risk of 2 years mortality [5].

Depression, quality of life and general well-being is all part of the chronic stress concept [6]. Stress is vaguely defined but is generally accepted as a risk factor for a poor outcome in IHD [7]. Chronic stress is associated with cardiovascular re-events and death from IHD and patients with MI have been shown to have higher stress levels when measured both as stress at work, stress at home, financial stress and major life-event stress [7], [8].

Chronic stress and depression is associated with widespread increased pain sensitivity, leading to both hyperalgesia (pain induced by noxious stimuli) and allodynia (pain induced by non-noxious stimuli) [9], [10]. The increase in pain sensitivity might be due to the neuroplastic effects of chronic stress on pain circuitry i.e. the diffuse noxious inhibitory control system (DNIC) [9], [11]. DNIC is an endogenous pathway mediating inhibition of lamina I neurons in the spinal dorsal root when pain signals ascend from the periphery through sensory C fiber neurons distributed wide spread over the body in the epidermis and up through the spinal cord [11]. Patients with hypersensitivity to pain have been shown to have an impaired DNIC modulation [12], [13]. Theoretically an intervention aiming at restoring this afferent-efferent disturbance may be beneficial for treating the increased pain sensitivity and at the same time lowering the stress-level and depressive symptoms. It has previously been theorized that therapies such as acupuncture may exert their effects through activation of DNIC [11].

The gold standard in measuring pain sensitivity is by algometry. Recently a simple and handheld algometric device has been designed to asses pressure pain sensitivity (PPS) [14]. The PPS measure has in patients with IHD been found to be significantly correlated to the major depression inventory score (MDI), WHO-5’s well-being index as well as to the SF-36 quality of life (QOL) score [15].

Several studies have evaluated the effect of various stress-reducing interventions in patients with IHD and some have shown to improve the prognosis and to reduce the risk of new cardiovascular events [16], [17]. We hypothesize that an intervention built on an increased focus on stress and the ability to perform stress reduction should be beneficial for patients with IHD. In analogy to people with diabetes measuring blood glucose levels, a therapy based on a daily semi-objective stress-measurements based on PPS followed by stress-reducing actions theoretically leads to increased empowerment and may have a positive effect on stress-parameters.

Acupressure, i.e. applying a continuous pressure for approximately one minute at specific hyperalgesic points at the body, has been shown to reduce both local and spreading pain in chronic low back pain and neck pain syndromes [18], [19] and we have observed that acupressure results in an acute reduction in pain sensitivity and PPS [20].

Thus we hypothesized that the combination of daily self-measurements of PPS aiming at increased empowerment followed by acupressure aiming to restore DNIC, together would resolve in a reduction of the following elements of chronic stress: Depressive symptoms measured by MDI, general well-being measured by WHO-5, and physically and mentally QOL measured by SF-36 QOL-score.

Aiming to test this hypothesis we performed an observer blinded randomized clinical trial with blinded outcome assessment over 3 month in which the active group measured PPS twice daily followed by acupressure as mandatory action. Our primary end point was changes in MDI.

## Methods

### Ethics Statement

The original protocols and a CONSORT checklist are available as supporting information (see Protocol S1 (Danish), Protocol S2 (English), Checklist S1). The study was approved by the local ethical committee (The Regional Ethical Committee of the Copenhagen Region, Denmark, Kongensvaenge 2, DG-3400 Hilleroed, www.regionh.dk/vek, identifier: H-4-2010-135, and amendment 31962) and the Danish Data Protection Agency (identifier: 2011-41-7022), and was registered on www.clinicaltrials.gov (identifier: NCT01513824). All participants gave their written informed consent after oral and written information about the study. The study was performed according to the declaration of Helsinki.

### Study Population

**Figure 1:**
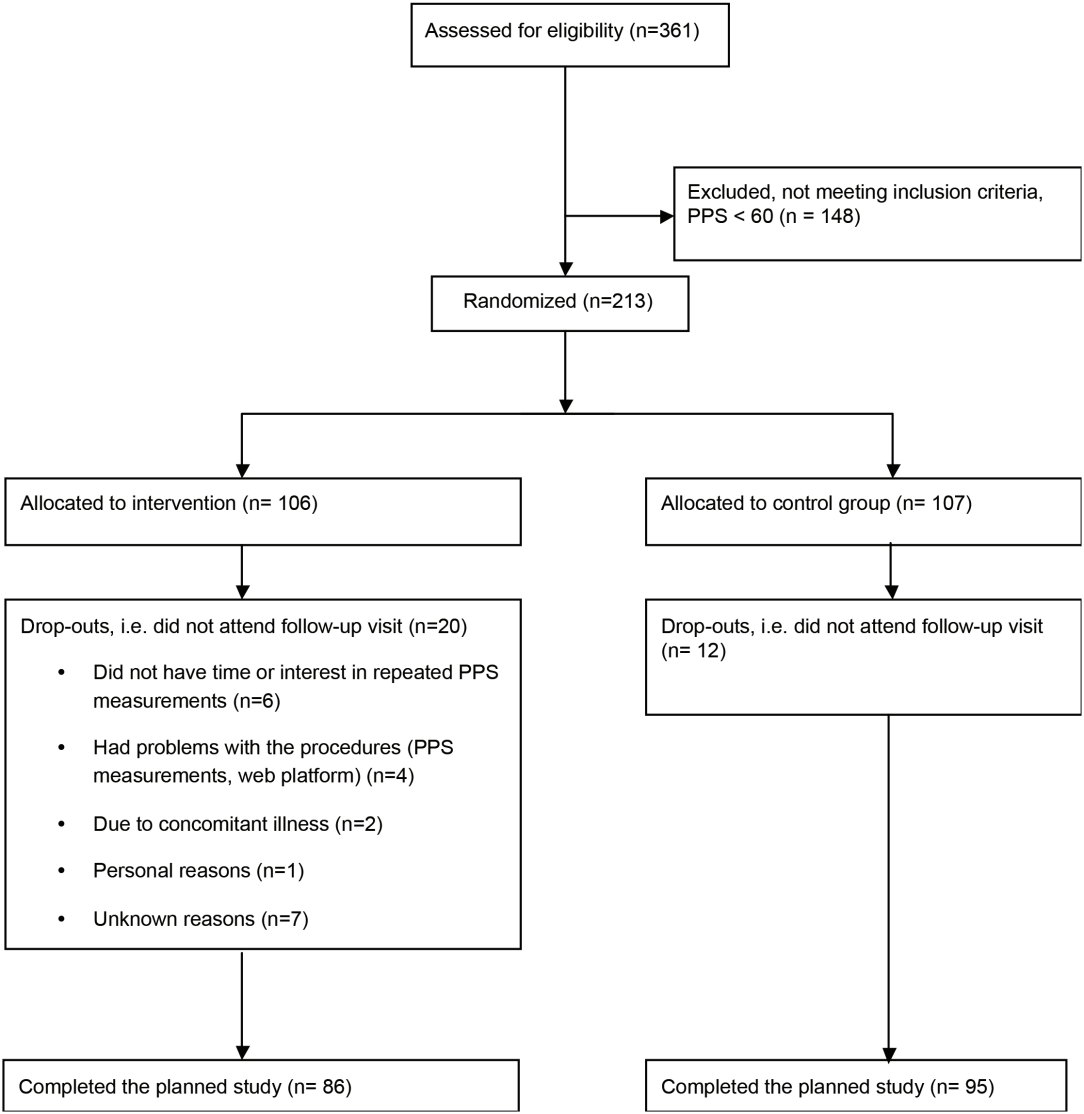
Consort.

361 patients with IHD participated in a cross-sectional study on the associations between PPS and stress in patients with IHD [15]. From previous studies it seems that PPS≥60 indicates an increased chronic stress level [21]. Participants from the cross-sectional study with PPS≥60, were enrolled in the RCT. Two hundred and thirteen of the 361 patients from the cross-sectional study fulfilled the criteria of having PPS above 60 (see consort, Fig. 1). All participants fulfilled the following additional inclusion criteria and none of the exclusion criteria: *Inclusion criteria*: 1) documented IHD defined as having had a MI, percutaneous coronary intervention (PCI) or coronary artery bypass graft surgery (CABG), 2) completed cardiac rehabilitation more than six months prior to inclusion, 3) age 75 years or younger at inclusion; *exclusion criteria*: 1) hospitalization due to psychiatric disease prior to IHD, 2) scheduled cardiac surgery, 3) changes in heart medication within the last month prior to inclusion, 4) a chronic competing disorder clearly impairing the patients QOL, 5) chronic pain syndromes such as fibromyalgia or severe arthritis [15].

### Randomization

After the baseline visit the participants were allocated randomly with a 1∶1 ratio to either the intervention arm or to treatment as usual (TAU). Randomization was stratified according to age, sex, MDI, diabetes, and chronic heart failure. The randomization was performed and carried out by an external statistician using a computerized randomization sequence with a block size of 16, which was unknown to the investigators. The participants received the allocation result by a confidential e-mail generated automatically. One hundred and six participants were allocated to active intervention and 107 to TAU.

### Study Design

A single center, two-armed, parallel-group, observer-blinded randomized clinical superiority trial. The participants were enrolled at the trial cite in Copenhagen (Denmark).

### Intervention Procedure

#### Active group

All subjects were instructed by a professional instructor (who had undergone education in PPS instruction and measurement and performing acupressure by passing a 4 week course plus a practical examination) to perform PPS measurements twice daily, in the morning (before breakfast) and in the evening (before going to bed). After the measurement, if the PPS level and thus the stress-level was high, the participant should perform acupressure as a mandatory stress-reducing procedure, as well as reflection on both the PPS level and on ones general feeling of need for stress handling on a voluntary basis.

All subjects received a personal PPS measurement instrument, together with an instruction manual and were individually instructed during a 45 minutes period at their private homes on how to use the PPS device. This included education in performing acupressure. The acupressure procedure has previously been described in “Magnusson et al,” [22]. In brief acupressure is performed by applying finger pressure for one minute sufficiently enough to feel the pressure but without causing pain. The pressure is applied on two points of the body: at the sternum, at the level of the fourth intercostal space and on the back 1.5 inch lateral to the spinal process of the fourth and fifth thoracic vertebra [22].

The participants were offered a new appointment and/or 1–2 phone contacts with the instructor if they had problems with self-measurements of PPS after the visit, or had other questions.

The participants were instructed to report their PPS measurements each day on their personal login on the website www.songheart.org. On the website each participant was able to track results and changes in PPS during the intervention period.

Both the active and the TAU group was informed at baseline, that their stress level was elevated and received a booklet on general stress handling principles [23].

### Predefined Endpoints

#### Primary end-point

Change in MDI from baseline to follow up after three months.

#### Secondary endpoints

Changes in stress-level as measured by PPS as well as changes in well being as measured by WHO-5.

#### Tertiary endpoints

Changes in quality of life as measured by SF-36 physical (PCS) and mental (MCS) QOL scale.

#### Predefined subgroup analysis

The stress-reducing effects in participants with the highest depression/stress level measured as the subgroup of participants with MDI≥15 at baseline and the subgroup of participants having the highest 50% of both PPS and Clinical stress signs score (CSS) at baseline.

### Outcome Measures

#### PPS

Before and after three months all participants came to the metabolic ward in the morning. The PPS level was measured by a professional after 5–10 min of resting in a supine position. The PPS measurement procedure has previously been described in details [14], [15]: The most sensitive area on the sternum was identified by palpation. The instructor applied a gradually increasing pressure with the PPS algometer on the most sensitive point until the pain threshold was reached. The PPS algometer transforms the pressure applied into a logarithmic scale of sensitivity level from 30 to 100. One hundred equals the highest sensitivity level, and thus the highest stress-level, 30 equals lowest measurable stress-level. An increase in 30 PPS units equals a 100% increase in sensitivity [14].

#### Questionnaires

A website was established for the study (www.songheart.org) and each participant received a personal profile with login. The day prior to the visit at the metabolic ward, the participants answered the following questionnaires on their www.songheart.org profile: 1) MDI, assessing depressive symptoms on a score form 0–50, zero being equivalent to no signs of depressive symptoms, 2) WHO-5, assessing psychological well-being with 100 equivalent to best psychological well-being, 3) SF-36, assessing PCS and MCS QOL with 100 equivalent to best QOL. At baseline a fourth questionnaire was included, measuring CSS as well as a demographic questionnaire. The CSS score is a newly developed score of 56 clinical stress symptoms experienced during the last four weeks [15]. The demographic questionnaire included questions on social status, employment status, cardiac medical history, co-morbidity and medication. This questionnaire should be answered before the baseline visit.

### Blinding

The professional instructor measuring PPS was blinded with regard to all results of online questionnaires as well as to the results of the randomization. Further the PPS device was designed in a way making the measure non-visible before the end of each measurement for both instructor and patient. The patients were instructed before randomization not to reveal the result of the randomization to the research personal performing the follow up investigations at three months. Statistical analysis was performed prior to the unveiling of the randomization codes.

### Estimation of Sample Size

In the original and approved protocol, sample size was calculated based on an anticipated effect size of 0.4, alpha of 0.05 and beta of 0.20 which corresponded to a sample size of 300 patients.

## Statistical Analysis

Data were evaluated by parametric statistics including paired and non-paired t-test on an intention to treat basis (ITT). Both the observed and values are reported. For testing of statistical significance we included all randomized patients in our analysis regardless of subsequent adherence to treatment. We imputed missing values treating the outcomes as dependent variables using multiple imputations. For this we used a linear regression model with the following predictor variables: Allocation, sex, and age. The imputation was conducted using 100 imputations and 20 iterations. The pooled estimates from these imputations were subsequently used for analysis [24].

Cohens effect size was used to compare active to TAU group using ITT data. The effect size was evaluated according to Hedgen and Olkin as the difference in mean change score from baseline to follow up between active group and TAU divided by the pooled standard deviation [25], [26]. In relation to clinically significant effects, the following have been proposed: Effect size<0.19, minor clinically significant effect; 0.20–0.39 small effect; the interval between 0.40 and 0.69, medium effect and >0.70, a large effect [27].

Analyses were performed using the statistical package SPSS version 19. All statistical tests were two-sided and p-values below 0.05 were considered statistically significant.

## Results

Table 1 shows the demographic characteristics of the participants from the active and TAU group respectively as well as together. There were no significant differences between the two groups regarding age, gender, psychometric measurements, social status, employment status, cardiac variables or risk factors, nor medication. However a group difference was found regarding co-morbidities on self-reported heart failure and self-reported diabetes mellitus (Table 1).

**Table 1:** Distribution of baseline factors according to treatment group.

	Full sample	Active group	Treatmentas usual	P-value
**N**	213	106	107	
**Male (n, %)**	156 (73%)	78 (74%)	78 (73%)	NS*
**Age in years (mean, SD)**	62 (8.1)	62 (8.1)	62 (8.2)	NS
***Psychometrics***				
**MDI (mean, SD)**	8.9 (7.4)	8.4 (7.7)	9.4 (7.0)	NS
**WHO-5 (mean, SD)**	65 (19)	67 (19)	63 (19)	NS
**PPS (mean, SD)**	81 (13)	81 (13)	81 (13)	NS
**SF-36 PCS (mean, SD)**	48 (8.4)	48 (9.1)	48 (7.6)	NS
**SF-36 MCS (mean, SD)**	52 (9.3)	53 (9.3)	52 (9.3)	NS
**CSS (mean, SD)**	9.7 (7.1)	9.2 (6.5)	10 (7.6)	NS
**Social status**				
**Married or cohabiting (n, %)**	175 (82%)	83 (78%)	92 (86%)	NS
**Have children (n, %)**	190 (92%)	97 (91%)	96 (90%)	NS
***Employment status***				
**Employed (n, %)**	106 (50%)	54 (51%)	52 (49%)	NS
**Unemployed (n, %)**	4 (2%)	3 (3%)	1 (1%)	NS
**Retired (n, %)**	92 (47%)	46 (44%)	52 (48%)	NS
***Cardiac variables***				
**Selfreported time (years) since** **diagnosed with IHD (mean, SD)**	7.5 (5.8)	8.2 (6.5)	6.8 (5.0)	NS
**Treated with PCI (n, %)**	147 (69%)	73 (69%)	74 (69%)	NS
**Treated with CABG (n, %)**	52 (24%)	27 (25%)	25 (23%)	NS
**Resting pulse (mean, SD)**	61 (11)	61 (11)	60 (11)	NS
**MAP (mean, SD)**	98 (10)	98 (9.7)	97 (11)	NS
***Cardiac risk factors***				
**BMI (mean, SD)**	27.6 (4.3)	27.8 (4.3)	27.4 (4.4)	NS
**Triglyceride (mean, SD)**	1.5 (0.9)	1.4 (0.7)	1.5 (1.0)	NS
**Current smoker (n, %)**	22 (10%)	9 (9%)	13 (12%)	NS
***Self reported co-morbidity***				
**Heart failure (n, %)**	72 (34%)	29 (27%)	43 (40%)	P = 0.047
**Chronic obstructive lung disease (n, %)**	13 (6%)	5 (5%)	8 (8%)	NS
**Diabetes (n, %)**	28 (13%)	20 (19%)	8 (8%)	P = 0.013
**Previous cerebral insults (n, %)**	15 (7%)	7 (7%)	8 (8%)	NS
**Have been treated for depression (n, %)**	32 (15%)	12 (11%)	20 (19%)	NS
***Medication***				
**Beta-blockers (n, %)**	125 (60%)	65 (61%)	60 (57%)	NS
**Cholesterol-lowering medication (n, %)**	188 (90%)	94 (89%)	94 (88%)	NS
**Calcium antagonists (n, %)**	47 (23%)	26 (25%)	21 (20%)	NS
**Angiotensin-II antagonist and/or** **ACE inhibitors (n, %)**	115 (55%)	56 (53%)	59 (55%)	NS
**Diuretics (thiazide or furosemide) (n, %)**	74 (36%)	40 (39%)	34 (33%)	NS
**Anti-depressive medication (n, %)**	12 (6%)	4 (4%)	8 (8%)	NS

*NS: p>0.05 between active group and TAU.

Twenty dropouts (19%) were reported in the active group and 12 (11%) in the TAU group, p = 0.18. (Fig. 1) Drop outs were similar to those who completed with regard to sex, age, MDI and WHO-5 scores.

### Adaptation of the Interventional Procedure

Ninety four in the active group reported repeated PPS measurements at home, in mean 90 measurements over three months (range 6–192).

### Primary Endpoint, MDI

MDI was significantly reduced by 22% after three months in the active group and by 12% in the TAU group. The reduction was significantly greater in the active group as compared with the TAU group, which resulted in a difference between the two groups at three months, p = 0.040 (Table 2).

**Table 2:** Results of questionnaires and PPS before and at three months follow up in.

	Statisticalanalyses	Treatment as usual(TAU), baseline(mean, SD)	Treatment as usual(TAU),follow up(mean, SD)	Baseline vs.follow up, P	Active group, baseline(mean, SD)	Active group, follow up(mean, SD)	Baseline vs.follow up, P	3 month analysisTAU vs. active,P	Effect size
**MDI**	ITT	9.40 (6.99)	8.31 (6.74)	0.025	8.36 (7.70)	6.48 (6.58)	0.001	0.040	0.12
	PP	9.34 (6.21)	8.32 (6.23)		7.93 (6.72)	6.12 (5.35)			
**WHO-5**	ITT	62.5 (19.0)	64.8 (20.9)	0.158	66.7 (19.1)	71.0 (18.3)	0.015	0.016	0.11
	PP	63.3 (18.4)	65.2 (19.2)		67.2 (19.1)	71.2 (15.4)			
**PPS**	ITT	80.9 (13.3)	72.1 (20.5)	<0.001	80.8 (13.3)	58.4 (22.1)	<0.001	<0.001	0.63
	PP	80.6 (13.3)	71.8 (17.5)		82.4 (12.3)	59.7 (20.6)			
**SF-36 physical component summary**	ITT	47.8 (7.68)	47.7 (9.61)	0.85	48.2 (9.14)	49.5 (9.99)	0.04	0.14	0.14
	PP	47.6 (7.90)	47.7 (8.56)		48.8 (8.50)	50.5 (6.47)			
**SF-36 mental component summary**	ITT	51.9 (9.31)	53.3 (9.72)	0.094	52.7 (9.35)	55.3 (9.34)	0.004	0.08	0.13
	PP	51.9 (9.23)	53.3 (6.92)		53.1 (9.28)	55.1 (7.84)			

Only statistical analyses using ITT data are presented.

ITT: intention to treat; PP: per protocol; TAU: treatment as usual; MDI: major depression inventory; PPS: pressure pain sensitivity; CSS: Clinical stress signs.

### Other Endpoints

WHO-5, increased by 6.4% in the active group and by 3.7% in the TAU group from baseline to follow up (p = 0.015 and p = 0.158, respectively), which resulted in significantly different levels between active group and TAU at 3 months (p = 0.016) (Table 2).

PPS was significantly reduced by 28% after three months in the active group and 11% in the TAU group (both p<0.001), and at 3 months the PPS values had become different between the two groups (p<0.001).

SF-36 PCS was improved in both groups although not reaching statistically difference at three months, p = 0.14. SF-36 MCS improved in the active group (p = 0.004), however, the difference after three month compared with TAU values only tended to be significant (p = 0.09) (Table 2).

### Subgroup Analyses

Two subgroup analyses were performed: One on subjects with MDI≥15 at baseline and another on subjects having both the PPS and CSS within the upper half at baseline (Table 3).

**Table 3:** Subgroup analyses.

MDI ≥15*	Statistical analyses	TAU: baseline(mean, SD)	TAU: follow up(mean, SD)	TAU: Baseline vsfollow up, P	Active group:baseline(mean SD)	Active group:follow up(mean, SD)	Active group:baseline vs follow up, P	Three monthfollow upTAU vs active, P	Effect size
**MDI**	ITT	20.5 (6.59)	16.1 (8.31)	<0.001	21.4 (6.19)	14.2 (7.55)	0.005	0.44	0.35
	PP	19.4 (4.91)	15.5 (8.19)		19.3 (4.18)	12.6 (6.86)			
**WHO-5**	ITT	39.8 (18.4)	51.1 (22.9)	0.015	41.3 (15.4)	56.8 (16.7)	<0.001	0.36	0.21
	PP	39.8 (17.5)	51.3 (22.7)		41.6 (17.3)	57.1 (16.1)			
**PPS**	ITT	85.0 (12.7)	78.7 (16.5)	0.017	86.1 (12.7)	71.9 (20.4)	<0.001	0.23	0.43
	PP	83.8 (12.5)	78.1 (14.3)		85.1 (13.1)	72.8 (20.0)			
**SF-36 physical** **component summary**	ITT	42.4 (8.91)	43.2 (8.76)	0.60	45.0 (10.7)	45.7 (9.93)	0.66	0.40	0.01
	PP	42.2 (8.88)	43.0 (8.43)		48.9 (9.61)	48.3 (8.32)			
**SF-36 mental** **component summary**	ITT	42.0 (11.3)	46.3 (8.71)	0.14	38.9 (8.58)	48.5 (11.5)	0.001	0.48	0.52
	PP	41.7 (11.0)	44.9 (7.39)		39.9 (10.0)	45.5 (10.4)			
**High PPS and high CSS** (PPS>81 and CSS≥8)**		**TAU: baseline** **(mean, SD)**	**TAU: follow up** **(mean, SD)**	**TAU: Baseline** **vs follow up, P**	**Active group: baseline** **(mean SD)**	**Active group:** **follow up** **(mean, SD)**	**Active group:** **Baseline vs** **follow up, P**	**Three month** **follow up** **TAU vs active,** **P**	**Effect size**
**MDI**	ITT	13.3 (9.8)	12.5 (8.6)	0.55	14.3 (9.9)	9.11 (8.30)	<0.001	0.15	0.52
	PP	12.1 (7.18)	12.1 (7.22)		12.3 (7.90)	8.25 (7.52)			
**WHO-5**	ITT	56.1 (21.7)	56.6 (19.9)	0.09	54.3 (22.9)	67.0 (19.6)	<0.001	0.066	0.62
	PP	57.7 (20.0)	56.7 (18.9)		55.2 (22.3)	68.6 (18.2)			
**PPS**	ITT	94.3 (5.62)	83.8 (15.3)	<0.001	96.4 (5.31)	77.3 (22.1)	<0.001	0.223	0.46
	PP	92.8 (6.19)	83.3 (13.1)		95.9 (6.06)	74.8 (23.5)			
**SF-36 physical** **component summary**		42.4 (7.93)	41.9 (9.50)	0.99	43.3 (9.41)	47.3 (9.75)	0.009	0.044	0.47
		42.2 (7.64)	42.3 (9.15)		47.6 (7.80)	51.8 (5.87)			
**SF-36 mental** **component summary**		48.6 (11.8)	49.5 (9.38)	0.64	47.3 (9.42)	52.9 (8.92)	0.004	0.199	0.51
		49.8 (11.0)	49.7 (9.05)		48.47 (9.42)	52.1 (10.2)			

Results of questionnaires and PPS before and at three months follow up in subjects from active and treatment as usual groups. Only statistical analyses using ITT data are presented.

ITT: intention to treat; PP: per protocol; TAU: treatment as usual; MDI: major depression inventory; PPS: pressure pain sensitivity; CSS: Clinical stress signs.

*) Forty-two participants had MDI≥15 at baseline. Twenty-one were randomized to active and 21 to TAU group. Thirty-six participants, 18 active and 18 controls completed the study.

**) Fifty-nine patients had PPS>81 and CSS≥8 at baseline, corresponding to the highest 50% of both PPS and of CSS. Twenty-nine

were allocated to active treatment and 30 to TAU, of whom 24 and 26, respectively, completed the study.

Fourty-two participants had MDI≥15 at baseline and 21 were randomized to active and 21 to TAU group, and 18 subjects in each group completed the study (Table 3). Fifty-nine patients had both PPS>81 and CSS≥8 at baseline, corresponding to the highest 50% of PPS as well as of CSS. Twenty-nine were allocated to active treatment and 30 to TAU, of whom 24 and 26 respectively completed the study (Table 3).

### Effect Size

The effect of active vs. TAU for the whole group was 0.12 for MDI, 0.11 for WHO-5 and 0.63 for PPS. Effect size for the subgroup with MDI≥15 was 0.35 for MDI, 0.21 for WHO-5 and 0.43 for PPS and for the subgroup with both the highest PPS and CSS, the effect size was 0.52 for MDI, 0.62 for WHO-5 and 0.46 for PPS.

## Discussion

In the present randomized interventional trial we found, that in patients with stable IHD the combination of daily self-measurements of PPS followed by acupressure and reflection on PPS as a surrogate for current stress-level resulted in a modest but statistically significant improvement in PPS, MDI and WHO-5.

This beneficial effect of the intervention program was more pronounced in subgroups of patients with higher baseline levels of components of the chronic stress syndrome, such as elevated MDI, as well as the combination of an elevated PPS and CSS. These subgroup analyses, however, were hampered by a limited sample size.

The primary endpoint was a reduction in MDI. MDI was chosen as a well validated often used psychometric questionnaire on depressive symptoms. It is well known that patients with IHD as well as other chronic diseases have increased levels of depressive symptoms which leads to a poor outcome [28]. The intervention resulted in a reduction in MDI of 22% in the active group while the TAU group only demonstrated a reduction of 12%, resulting in a significant difference between the active and the TAU group at follow up. This corresponded to a small effect size of 0.12. When looking at the subgroups, however, the reduction in MDI was much greater resulting in an effect size of 0.35 in the MDI≥15 subgroup representing participants with the most depressive symptoms and 0.52 for the high PPS high CSS group representing the participants with an elevated chronic stress level. These effect sizes are clinically significant [27]. Thus our findings point to a greater effect of our intervention among more psychologically vulnerable subjects. These data on effect size should be compared to the effect size of antidepressants found in placebo-controlled clinical trials of treatment resistant patients with overt depression of approximately 0.40, although our patients did not have overt depression [29]. Further, a meta-analysis on exercise training, a well-known and beneficial treatment, on depressive symptoms among patients with a chronic illness, demonstrated an effect size of 0.30 in patients with mild-to-moderate depression [30].

Similar to a positive effect on MDI we found a beneficial effect of the intervention on general well-being as measured by WHO-5′s well-being index. The intervention resulted in an increase in WHO-5 with a small effect size, however subgroup analysis demonstrated a substantially increase in well-being, although the difference at three months between the active and the TAU group, did not reach statistically significance probably due to a limited number of subjects in the subgroup analyses.

The intervention was based on the concept that self-measurement of pain sensation threshold measured by PPS resulted in an awareness of ones current stress-level, leading to reflection as well as to action i.e. increased empowerment, aiming at improving overall well-being.

Acupressure was the only mandatory action for reducing pain sensation in the intervention program. General advice about stress reduction was presented for both the active and the TAU group in the form of a booklet on stress-coping [23].

Parts of the intervention program have been used with success previously in patients with advanced angina pectoris in a non-randomized fashion [31], [32]. The intervention procedure was accepted by the participants of the active group, in terms of repeated PPS measurements reported the website. The participants reported in mean 90 measurements over the three month period. This is probably a lower bound since the participants might have performed PPS measurements without reporting them on the website. The current study serves as proof of concept concerning repeated PPS measurements at home followed by acupressure and reflection in general as a stress-reducing intervention.

PPS measures pain sensitivity threshold like other algometers within pain research. We have recently demonstrated a close correlation between PPS and another pressure pain algometer [15].

An inherent weakness of pressure pain algometry is the fact that there is no objective measure of pain threshold since it is the subject itself that reacts to discomfort and ends the measurement. Therefore a potential bias to repeated measurements is habituation both mentally and physically. If present this could result in a progressive reduction in PPS over time. We have performed validation studies in which we have found a very close association between two measurements performed within 5 seconds as well as with one day between measurements [31]. This suggests that a drift towards lowering of the PPS value due to repeated measurements is probably of minor importance.

In the present study the PPS measure was reduced with 22 units in the active group. The PPS device is constructed such that a reduction of 30 units equals a doubling of the pressure applied to the sternum. This quite robust change also speaks against a drift in PPS sensation due to habituation.

The instructors measuring PPS at baseline as well as after three months were blinded to the randomization results, the answers of the questionnaires, the results of the PPS home measurements and to the concrete PPS result while measuring at baseline and follow up visits. The latter is due to the construction of the PPS device, which first shows the result of the pressure when ending the pressure, thus revealing only the final result.

Chronic illness in general is associated with both stress and depressive symptoms which includes IHD [33]. Chronic stress and depression are associated with increased pain sensitivity, clinically presenting with widespread hyperalgesia [10]. A 28% reduction in pain sensitivity from baseline to follow up as found in the present study may be of clinical relevance.

Whether the reduction in pain sensitivity is the primary event leading to reduced feeling of depressive symptoms and increased well-being as found by an improvement in MDI and WHO-5 results, or the intervention works primarily by changing depressive symptoms and well-being and thereafter leads to a reduction in pain sensitivity is not clear. Concerning the mandatory intervention, acupressure, it is a well known clinical observation, that acupressure on a distinct sore point of the body leads to pain reduction locally with a spreading effect into the surroundings, which also has been demonstrated in females with chronic neck pain [18]. Further, the use of acupressure in low back pain has been proven in a RCT setting [19]. We have observed an acute reduction in PPS due to acupressure over the sternum, and it is postulated that acupressure works by reducing pain sensitivity by restoring DNIC [11]. If this is the primary effect of our intervention, the primary event will be reduced pain sensitivity. On the other hand the reflection on ones stress level based on repeated PPS measurements could also be the leading event resulting in increased empowerment which might result in a reduction of depressive symptoms and increased well-being as the primary effect. Several studies have found that psychological interventions including cognitive, behavioral and educational approaches all aiming at enhanced empowerment has been effective in reducing depression and anxiety in patients with IHD [16], [34]. Cognitive stress-reducing therapy has also been found to reduce recurrent cardiovascular events [17]. These studies imply that the empowering part of the intervention may be the primary effect.

### Strengths and Limitations

#### Limitations

We aimed at 300 and included 213 participants. The power analysis was based on an effect size of 0.40, but we only obtained an effect size of 0.12. This means that the study ended up underpowered. However looking at both primary endpoint (MDI) and the other endpoints we found a rather clear tendency towards a beneficial effect on all parameters studied during intervention. This indicates that all bights the effect size was rather small, it seems real and is not a result of a type 1 error. Although the study was underpowered we did find significant changes probably avoiding a rather large risk of a type 2 error.

The randomization procedure included DM and chronic heart failure yes/no, however, the randomization ended up with significantly more diabetics in the active group and in contrast less patients with chronic heart failure compared with the TAU group. Given the small number of diabetics and patients with heart failure the consequence of this randomization failure is probably mild or absent.

The study was single blinded. This was intentionally since the intervention was of an open nature i.e. the participants should reflect on the PPS measurements and also because sham PPS measurement giving false values did not seem ethically acceptable. However, the single-blinded design may have caused bias to the results of the intervention and may have affected the responses from the participants regarding the questionnaires. With regard to the PPS measurements they were performed by professionals who did not have knowledge about the allocation to active or control group. Further the PPS device was designed in a way making the measure non-visible before the end of each measurement for both instructor and patient. Thus the results from PPS measurements can be regarded as double blinded.

#### Strengths

Rather few in- and exclusion criteria make our study representative to most patients with IHD, further the intervention technique used is easy to adapt and use, and without side effects.

Statistical evaluation was performed by an ITT analysis using all randomized subjects (n = 106). This is the most correct, however not always used in RCT [35].

### Conclusions

Self-measurements of PPS followed by acupressure as a mandatory action and otherwise individual reflection, in subjects with stable IHD resulted in a reduction in PPS, reduced amounts of depressive symptoms and increased well-being. This effect seemed more pronounced among the most psychologically vulnerable subjects. Due to the study ending up being underpowered further studies which are properly powered should be performed in order to establish the impact of this new type of intervention.

### Perspectives

The concept of biofeedback guided stress handling, i.e. increased patient empowerment, based on frequent self-measurement of PPS might be broadened out to other chronic illnesses like diabetes, as well as to otherwise healthy subjects on work places where the employees demonstrate signs of increased stress due to a high psychological stress demand.

## Supporting Information

Protocol S1 **Trial Protocol (Danish).**
(PDF)

Protocol S2 **Trial Protocol (English).**
(PDF)

Checklist S1 **CONSORT Checklist.**
(DOC)
