# Supplementary material for: The Effect of Daily Self-Measurement of Pressure Pain Sensitivity Followed by Acupressure on Depression and Quality of Life versus Treatment as Usual in Ischemic Heart Disease: A Randomized Clinical Trial
Source: PLoS One. 2014 May 21;9(5):e97553. doi: 10.1371/journal.pone.0097553 (PMC4029626; doi:10.1371/journal.pone.0097553)
Supplement: Protocol S1 — Trial Protocol (Danish). (PDF) [file pone.0097553.s001.pdf]

PROTOKOL SongHeart studiet

Stress, ongoing self monitoring and ischemic heart disease rehabilitation, a randomized controlled trial.

(dansk titel: Selv monitorering af stress i hjerterehabilitering, en randomiseret undersøgelse)

Version 10, dateret 110415; VEK reg. Nr. 27890

Projekt ansvarlig

Jens Faber, professor dr.med. [1)]

Styregruppe

Per Bech, professor dr.med. [6)]

Åke Hjalmarson, professor dr.med. [5)]

Søren Ballegaard, speciallæge. [4)]

Deltagere

Finn Gyntelberg, professor dr.med. [3)]

Lars Juel Andersen, dr.med., Ph.D.[2)]

Åse Marie Hansen, senior forsker, cand pharm. Ph.D. [3)]

Jesper Kristiansen, senior forsker, M.Sc., MD Sci. [3)]

Kjeld Møller Pedersen, professor dr.med. [7)]

Lars Arendt-Nielsen, professor dr.med. [8)]

Caroline Kistorp, overlæge Ph.D. [1)]

[1] Medicinsk Afdeling O, Endokrinologisk Enhed

Herlev Hospital

Københavns Universitet

Herlev Ringvej 75

2730 Herlev

E-mail: jenfab01@heh.regionh.dk

Telefon: 38 68 90 16, mobil: 23 23 51 34

[2] Kardiologisk Afdeling P,

Gentofte Hospital

Niels Andersens Vej 65

2820 Gentofte

E-mail: lajuna01@geh.regionh.dk

Telefon: 39 77 22 61, mobil: 51 50 27 57

[3] Det Nationale Forskningscenter for Arbejdsmiljø

Lersø Park Alle 105

2100 København Ø

E-mail: aamh@arbejdsmiljoforskning.dk

E-mail: jkr@arbejdsmiljoforskning.dk

[4] Ull Care A/S

Lemchesvej 1

2900 Hellerup

Telefon: 39 40 41 42, mobil: 40 19 26 45

E-mail: sba@ullcare.com

[5] Kardiologisk Institut

Sahlgrenska Universitetshospital

Gøteborgs Universitet

SE 413 45 Gøteborg, Sverige

E-mail: ake.hjalmarson@gu.se

[6] Psykiatrisk Forskningsenhed

Psykiatrisk Center Nordsjælland

Dyrehavevej 48

3400 Hillerød

Telefon: 48 29 32 53

E-mail: per.bech@regionh.dk

[7] Sundhedsøkonomi

Syddansk Universitet

Københavns Afdeling

Øster Farimagsgade 5 A, 2.
1399 København K

Telefon: 65 50 38 47
E-mail: kmp@sam.sdu.dk

[8] Institut for Sundhedsvidenskab og Teknologi, Center for Sanse-motorisk Interaktion

Aalborg Universitet

Frederik Bajers Vej 7

9220 Aalborg

Telefon: 99 40 88 30

E-mail: lan@hst.aau.dk

Anvendte forkortelser:

HRV: Heart rate variability: et mål for balancen mellem det sympatiske og parasympatiske nervesystem.

MDI: Major Depression Inventory. Et spørgeskema der giver et mål for graden af depression

PPS: Pressure Pain Sensitivity på brystbenet. Er en markør for fysiologisk stress.

PRP: Pressure-Rate Product; systolisk blodtryk x puls. Målet er en markør for hjertets arbejde og hjertets iltforbrug

SF 36: et spørgeskema, der giver et mål for generelle fysiske og psykiske helbred samt livskvalitet

QOL: quality of life, livskvalitet

Resume

Baggrund: Stress og depression har vist sig at være gensidigt forbundne samt at have en selvstændig prognostisk indflydelse på hjertepatienters overlevelse. Et nyt biologisk mål, der måler følsomheden af tryk på brystbenet (Pressure Pain Sensitivity, PPS), har vist sig at være en pålidelig markør for fysiologisk stress samt forbundet med grad af depression og livskvalitet. En intervention der bruger PPS målet som en biologisk feedback markør for stress har vist sig at reducere stress og depressions niveauet samt øge livskvalitet.

Formål: Det primære formål med studiet er at undersøge, om en simpel individualiseret egenomsorgsbaseret intervention, der bruger PPS som biofeedback markør for stress, reducerer niveauet af depression og stress og øger livskvalitet hos patienter med iskæmisk hjertesygdom.

Design: Et prospektivt, randomiseret enkelt-blindet klinisk forsøg, hvor effekten af et egenomsorgsbaseret interventionsprogram sammen med den bedste behandling indenfor hjerterehabilitering (REHAB + ULL) bliver sammenlignet med en kontrolgruppe, der alene modtager den bedste behandling indenfor hjerterehabilitering (REHAB + CARE) over en 3 måneders periode. Studiet er åbent for patienten og den professionelle instruktør, men blindet for de evaluerende forskere.

Patienter: 300 patienter med iskæmisk hjertesygdom, som har været igennem et hjerterehabiliteringsforløb på henholdsvis Gentofte og Herlev Hospital.

Effektvariabler: Den primære effektvariabel er graden af depression. De sekundære effektvariabler er graden af stress målt med PPS og livskvalitet.

Behandling: Alle patienter har været igennem samme praksis indenfor hjerterehabilitering. Halvdelen af dem, valgt ved randomisering, modtager i tillæg et egenomsorgsbaseret interventionsprogram, der fokuserer på daglig PPS måling for kognitiv refleksion og nerve stimulation for at reducere stress.

Etik: Alle patienterne fortsætter uændret med standard hjerterehabiliterings behandling. Det egenomsorgsbaserede interventionsprogram er en risikofri intervention.

Projekt organisation og flow: Jens Faber leder projektet. Den medicinske vurdering af patienter vil blive udført af blindet sygeplejerske i samarbejde med en læge. Den samlede periode for projektet er 1 år.

Perspektiv: Hvis studiet møder succeskriterierne, konkluderes det at interventionen er mulig

og evidensbaseret med henblik på at reducere graden af depression og stress hos hjertepatienter.

1. Videnskabelig baggrund

1.1 Prognostiske faktorer ved hjertesygdom med speciel henvisning til det foreliggende forsøg:

Der er international konsensus om at de følgende faktorer har en prognostisk indflydelse i forhold til hjertepatienter med iskæmisk syge og deres overlevelse: serumkolesterol (Lewington et al., 2007); blodtryk (Bibbins-Domingo et al., 2010;Lewington et al., 2002); hjertets arbejde målt som systolisk blodtryk gange puls (PRP) (Elhendy et al., 2003); depression (Sher et al., 2010;Surtees et al., 2008); kronisk stress (De et al., 2003;Dimsdale, 2008;Holmes et al., 2006; Orth-Gomer et al., 2009).

1.2 Definitioner og forudsætninger med hensyn til stress

Selvom stress defineres og måles forskelligt inden for forskningen, er der dog almindelig enighed med hensyn til følgende to aspekter:

1) Der skelnes mellem forbigående (akut) og vedvarende (kronisk) stress:

- *Forbigående stress* er en fysiologisk tilstand af beredskab, en tilstand, der automatisk induceret gennem neurale og hormonelle signaler fra hjernen, når en trussel eller udfordring opstår. Beredskabet fungere som en forsvarsmekanisme og bidrager til en øget ydeevne, uanset om denne skal benyttes i en flugt situation eller til at løse en arbejdsopgave. Når truslen eller udfordringen er overvundet, slukkes beredskabsfasen og kroppen genetablerer homeostasis.
- *Vedvarende stress* opstår ved en langvarig eksponering af de hormoner, der er involveret i forbigående stress hvilket kan medføre psykologiske og fysiologiske ændringer, som på sigt kan være skadelige for helbredet.

2) Omfanget af vedvarende stress afhænger af balancen mellem miljømæssige krav og den enkeltes forventning til resultatet, samt opfattelsen af balancen mellem krav og tilgængelige ressourcer. Derudover har den individuelle tilpasningsevne også betydning for niveauet af stress.

1.3 Stress, depression og iskæmisk hjertesygdom

I de seneste år er der kommet øget fokus på koblingen mellem hjertesygdom, depression og kronisk stress (Grippo and Johnson, 2009). I forlængelse heraf er der opstået evidens for et sammenfald af væsentlige metaboliske forandringer for følgende tilstande: hypertension,

hjertesvigt, fedme, diabetes mellitus, metabolisk syndrom, kronisk stress og depression samt andre psykiske lidelser (Koschke et al., 2009;Szczepanska-Sadowska et al., 2010).

Forekomsten af depression, målt ved MDI score ≥ 20, er fundet at være cirka 20 % hos patienter med iskæmisk hjertesygdom (Olesen 2003; Bech 2001)

1.4 Måling af stress

Der er hidtil ingen international konsensus omkring biokemiske diagnostiske metoder til måling af forbigående og vedvarende stress (Ekman and Lindstedt, 2002;Noble, 2002). Dette skyldes bl.a., at det er vanskeligt at identificere et enkelt fysiologisk mål for stress, fordi stress involverer flere faktorer, der hver især har været brugt som markører på stress i forskningen. Disse er: 1) objektive miljømæssige stimuli, 2) den enkeltes opfattelse af stimuli, 3) coping-mekanismer, samt 4) fysiologiske reaktioner. Disse fire faktorer er ikke nødvendigvis til stede samtidigt, hvilket har kompliceret målingerne yderligere (Holmes et al., 2006).

1.4.1 Biologiske varslingssystemer og stress (PPS målet)

Biologiske varslingssystemer har altid været essentielle for overlevelses af levende organismer (Kumazawa, 1998a). Hos dyr er evnen til at opdage noget i miljøet, som kan være en trussel, udviklet til bl.a. et nociceptivt system, der involvere en refleks, som får dyret til at trække sig tilbage fra en potentiel truende stimuli. Det nociceptive system er baseret på en polymodal receptor, en udifferentieret nervecelle af samme art i hele den evolutionære kæde fra fisk til højere hvirveldyr og mennesker (Sneddon et al., 2003). Receptoren stimuleres af mekanisk tryk, temperatur og surhedsgrad.
Følsomheden af receptoren kan undergå forskellige former for graduering, et eksempel er stress-induceret analgesia (smertelindring), hvor smerte sensation bliver undertrykt (Lewis et al 1980). Denne analgesia hjælper det sårede dyr til at undertrykke generelle smerte fornemmelser, for at kunne kæmpe videre eller flygte. Et andet eksempel er stress-induceret hyperalgesia (smerteoverfølsomhed), hvor smerte fornemmelsen øges. Dette afspejles i dyreforsøg som en øget følsomhed i pote-tryktests samt hale svirp tests (Meagher et al., 2001;Pilcher and Browne, 1983).

Kliniske observationer af hjertepatienter viser, at en forhøjet tonus i det sympatiske nervesystem forsager en øget ømhed i bestemte områder på thorax. En pilotundersøgelse af 250 raske personer viste en signifikant sammenhæng mellem graden af ømhed i særlige områder på thorax til antallet af kliniske stresstegn. Disse observationer er senere blevet bekræftet i et tværsnitstudie (Ballegaard et al., 2009). Sådanne observationer har ikke tidligere været beskrevet som en del af varslingssystem i mennesker, men fra et evolutionsbiologisk synspunkt, forbedres overlevelsespotentialet ved begge aspekter af stress induceret modulering, ved hjælp af en mekanisme, der kan påvises så langt tilbage i udvikling som fisken (Sneddon et al., 2003).

1.4.1.1 Sensoren, PPS målet og kendte markører for stress

Polymodale sensoriske nerver er ansvarlige for nociception og sender deres impulser gennem små myeliniserede A-delta fibre eller umyeliniserede C-fibre (Kumazawa, 1998). De er følsomme over for sympatiske input (Nilius et al., 2004). De polymodale sensoriske nerver i brystbenet har vist sig at øge deres følsomhed under stress (Ballegaard et al., 2009). Dette kan måles ved hjælp af et nyt instrument der registrerer følsomheden overfor smerte påført ved tryk (Pressure Pain Sensitivity, PPS). PPS målingen er simpel, og har vist sig at være meget reproducerbar, både målt af den enkelte person samt af professionelle (Ballegaard et al., 2009), og gentagne målinger synes ikke at medføre systematiske ændringer betinget af tilvænning til måleudstyr. Det betyder, at PPS kan blive et nyt værktøj for stresshåndtering: Daglige målinger efterfulgt af refleksion og handling, på samme måde som blodsukker måling hos diabetes patienter. Et interventionsprogram er udviklet til personlig PPS baseret bio-feedback guidet stresshåndtering (se punkterne 1.5 og 9.2.2).

Det nye medicinske mål har vist sig at være knyttet til kendte markører for forbigående stress som puls, blodtryk, spyt kortisol samt hjertets arbejde målt som Pressure-Rate-Product (PRP)(Ballegaard et al., 2009). PRP har vist sig at være knyttet til stress hos patienter med iskæmisk hjertesygdom (Jain et al., 2001).

Hvad angår markører for vedvarende stress, har PPS hos 292 kontorarbejdere vist sig at være knyttet både til antallet af kliniske stress tegn og til forekomsten af depression målt ved IC 10 Major Depression Inventory (MDI) (Ballegaard et al., 2011). Depression har vist sig at have prognostisk indflydelse på iskæmisk hjerte patienter (Surtees et al., 2008). PPS var også koblet til generelt psykisk og fysisk helbred målt ved SF 36 spørgeskema (Ballegaard et al.,

2011) – et spørgeskema, som afspejler livskvaliteten hos patienter med iskæmisk hjertesygdom (Dempster and Donnelly, 2000;Oldridge et al., 2002).

1.4.1.2 Validitetsstudier af selve PPS målingen

I samarbejde med forskere fra Det Nationale Forskningsinstitut for Arbejdsmiljø, Columbia University i New York og Københavns Universitet, er der foretaget en række validitets studier af selve PPS målingen med henblik på, at belyse følgende forhold:

1. Reproducerbarheden, evnen til at reproducere samme måleværdier ved gentagende målinger, er undersøgt i to særskilte situationer med henholdsvis 5 sekunder og en hel dag mellem de to målinger.
2. Kategoriserings evne og dennes reproducerbarhed; evnen til at identificere grupper af personer med en højere helbredsrisiko end en anden gruppe + evnen til at reproducere denne kategorisering.
3. PPS målets specificitet og sensitivitet, når andre helbreds markører bruges som kategoriserings variable
4. Risikovurdering med hensyn til systematiske ændringer i PPS målet forårsaget af tilvænning til måleudstyret.

Ad 1: Reproducerbarhed

a) Undersøgelser med 5 sekunder mellem de to PPS målinger
    a. Hos 181 patienter i en ambulant lægeklinik blev målingerne foretaget af en professionel måler og viste høj overensstemmelse mellem første og anden måling (korrelations koefficienten $r = 0{,}97$, $p < 0.001$) med en gennemsnitlig forskel (mean) på 0,3 måleenheder ($p > 0{,}5$), og en standard afvigelse (SD) på 5,7 måleenheder.
    b. En høj overensstemmelse mellem 1. og 2. måling var også fundet da 10 forskellige professionelle målere målte 292 kontorarbejdere ($r = 0{,}90$, $p < 0.001$; gennemsnitlig forskel 1.32 måleenheder, SD = 7,40 måleenheder).
    c. I et studie af 35 operasangere der målte sig selv 2 gange dagligt i en 2 ugers periode, i alt 698 sæt PPS målinger ($r = 0{,}95$, $p < 0.001$; gennemsnit forskel = - 0,32 måleenheder, SD = 7,6 måleenheder) (Ballegaard et al., 2011).

b) En Bland-Altmann analyse på de 3 ovennævnte studier (a, b og c) viser en ensartet måleforskel mellem 1. og 2. måling over hele måle skalaen (Ballegaard et al., 2011).

c) Undersøgelser med en hel dag mellem de to PPS målinger
   a. I studiet med 35 opera sangere, der målte sig selv to gange dagligt i to uger, var der en signifikant korrelation mellem de i alt 318 sæt dagsmålinger (morgen og samme dags aften); $r = 0.91$ ($p < 0.0001$); gennemsnitlig forskel: 2,2 enheder (SD = 10,5 enheder)($p < 0.001$).
   b. I et andet studie hvor 60 kontor arbejder målte sig hjemme i 3 måneder, var der også en signifikant korrelation mellem de i alt 1854 sæt dagsmålinger: $r = 0.87$ ($P < 0.0001$), gennemsnitlig forskel + 2,2 enheder (SD 8,9)($p < 0.001$).

Ad 2: Kategorisering

a) PPS målets evne til en 2-delt klassifikation af personer i ”vedvarende stressede” (= PPS ≥ 60) og ”ikke vedvarende stressede” (= PPS < 60) er undersøgt via en ROC analyse i studiet af de 292 kontorarbejdere (Ballegaard et al 2011) i forhold til en analoog 2-delt klassifikation ”havende en forhøjet helbreds risiko markør” og ”ikke havende en forhøjet helbreds risiko markør” for følgende effekt variable og ud fra følgende diskriminations kriterier: 1) antallet af kliniske stress tegn (færre end 10/ 10 eller flere kliniske stress tegn)(gennemsnitlig PPS 49/58; $p < 0.001$), 2) SF-36 (over eller under 25 % percentilen ift den danske normale befolkning matchet for køn og alder): generelt fysisk helbred (PCS skala)(gennemsnit PPS 49/56; $p = 0.004$) og psykisk helbred (MCS skala) (gennemsnit PPS 49/57; $p = 0.004$), samt 3) forekomsten af depression målt via spørgeskemaet Major Depression Inventory (MDI score under 20/lig med eller højere end 20)(gennemsnitlig PPS 51/61; $p = 0.012$). For alle de 3 nævnte variable var der således signifikant højere PPS mål for personer, der indledningsvist var kategoriserede som værende i den gruppe med højere stress niveau.
b) PPS målets evne til at reproducere klassifikation af personer ved gentagende målinger er belyst i to aspekter med henholdsvis 5 sekunder og en hel dag mellem de to målinger:
   a. Undersøgelser med 5 sekunder mellem de to målinger:
      i. 10 forskellige instruktørers målinger på 282 kontorarbejdere; sandsynlighed for ensartet klassifikation (PPS ≥ 60): 89 %
      ii. 698 egen PPS målinger hos 35 operasangere; sandsynligheden for tilsvarende ensartet klassifikation: 82 %

iii. To forskellige instruktørers målinger på 181 ambulante patienter. Her var den tilsvarende sandsynlighed 83 %.
iv. Indføres en tolerance på 1 standard afvigelse for anden måling, ville de samme sandsynligheder være 97 %, 95 % og 98 % respektivt (Ballegaard et al., 2011);

b. Undersøgelser med en hel dag mellem de to målinger
  i. sandsynligheden for en ensartet klassifikation (PPS ≥ 60) er 87 % når alle 6004 morgen PPS målinger på Ull Care's webplatform per 12.april 2011 sammenlignes med PPS målinger foretaget samme dags aften (Ballegaard et al 2011).
  ii. Sandsynligheden for en tilsvarende ensartet klassifikation blandt de 318 dagsmålinger, som blev foretaget af 35 opera sangere over en 2 ugers periode, var 83 %.

Ad 3: PPS målets specificitet og sensitivitet

Når de ovenfor nævnte diskriminations kriterier anvendes til en todelt kategorisering i forhold til gruppering ud fra et PPS mål på 60 måleenheder, fandtes en specificitet på 0,79 samt en sensitivitet på 0, 32, hvad angår forekomsten af forhøjet risiko profil i forhold til én eller flere af de nævnte markører; antallet af kliniske stress tegn, psykisk og psykisk helbred målt ud fra SF 36 samt forekomsten af depression målt ud fra MDI. Resultatet skønnes acceptabelt, idet den høje specificitet sikre, at en stor procentdel af de personer der ikke har behov for behandling, heller ikke får tilbudt en. Den lave sensibilitet kan medføre, at nogle personer får tilbudt behandlingen, selvom behovet ikke er til stede, men da behandlingen er harmløs og uden bivirkninger skønnes dette acceptabelt.

Ad 4: Risikovurdering med hensyn til tilvænnings bias ved gentagne PPS målinger

De hidtil gennemførte undersøgelser kan ikke med sikkerhed udelukke, at måleadaption påvirker måleresultat, når PPS måles dagligt over længere tid, men nedenstående undersøgelser taler for at en sådan indflydelse ikke har udslagsgivende betydning i patienternes kliniske hverdag.

a) En gennemgang af den videnskabelige database PUBMED har ikke kunnet give videnskabelig belæg for at fænomenet eksister, hvilket i sig selv naturligvis ikke betyder, at fænomenet ikke eksisterer.

b) I to case-control database studier hos patienter med henholdsvis iskæmisk hjertesygdom og apopleksi og med henholdsvis 3 og 4 års observationstid, brugte den enkelte patient den daglige PPS måling anvendt som en biologisk bio-feedback markør for stress-niveauet med hensyn til kognitiv refleksion over sit stress niveau. Resultaterne viste et markant fald i brugen af sundhedsydelser samt en forbedret overlevelse i begge studiet (Ballegaard et al., 2004;Magnusson et al., 2010).

c) I to randomiserede interventionsundersøgelser med henholdsvis 50 opera sangelever og 292 kontorarbejdere og med henholdsvis 6 og 3 måneders observationstid, var et fald i PPS koblet til signifikante og klinisk relevante forbedringer i kendte risiko faktorer som blodtryk, puls, hjertets arbejde og serumkolesterol. (Ballegaard et al., 2011)).

d) En statistisk analyse af 6004 egen-PPS-målinger på Ull Care webplatformen, hvor mindst et af dagens PPS mål er >30 viser, at der er en signifikant stigning i gruppen som helhed fra morgen til samme dags aften (gennemsnitlig stigning (mean) = 2,9 måleenheder; SD = 8,4; $p < 0,001$), samt at der er et tilsvarende fald i PPS fra sengetid til næste morgen (mean = - 3,1 måleenheder; SD = 8,6, $p < 0,001$)(Ballegaard et al., 2011).

e) En mulig forklaring på den fortsatte anvendelighed af PPS målet kan skyldes det forhold, at det sjældent er den samme polymodale sansecelle, der er den ømmeste to dage i træk, hvorfor måle punktet flyttes til en ny sansecelle, hvorfor risikoen for smertetærskel adaptation på grund af repetitiv sensorisk stimulation er begrænset. Den enkelte polymodale sansecelle har et måleområde på cirka 1 kvadrat mm (Kumazawa T 1998), måle fodpuden et areal på cirka 1 kvadrat cm og selve måle området er håndflade stort, svarende til cirka 50 kvadrat cm.

Sammenfatning på validitets-undersøgelser.

- Der er en tilfredsstillende overensstemmelse mellem gentagne målinger, foretaget såvel med 5 sekunders mellemrum, som morgen og aften på samme dag..
- En klassifikation af personer med et PPS ≥ 60 som tilhørende en gruppe personer med en forhøjet helbredsmæssig risikoprofil i forhold til personer med et lavere PPS mål, synes klinisk anvendelig og reproducerbar.
- En mulig klinisk udslagsgivende betydning af systematisk fejl opstået ved tilvænning til måleudstyret synes ikke at være tilfældet, hverken hvad angår evnen til at opnå relevante

kliniske effekter efter henholdsvis måneders eller års PPS måling, eller hvad angår muligheden for at bruge ændringer i PPS målet som en markør for stress i den daglige kognitive refleksion.

1.4.1.2 Compliance af hjemme PPS måling

I studiet med 35 opera sangere, der skulle måle sig to gange dagligt i to uger var compliance 99 %, mens den var 20 % blandt de 60 kontor arbejdere, der skulle måle sig dagligt i 3 måneder . Set i lyset af de resultater, der blev opnået blandt de 60 stressede kontor arbejdere, forekommer det ikke bekymringsværdigt, at deres måle compliance kun var 20 % målt over en 3 måneders periode. Årsagerne til den lavere compliance kan være mange: 1) at ikke alle målinger indtastes,; 2) at målinger ikke indtaster, når de i gennem længere tid har været lave (feks. < 40), 3) at det må lang sigt ikke er nødvendigt at måle dagligt, men måske bare få gange per uge – eller når behovet for cognitiv refleksion presser sig på.

1.4.2 Spørgeskemaer (MDI, SF 36, WHO 5)

Major Depression Inventory (MDI) er et validt og reproducerbart spørgeskema til måling af depression (Bech, 2010).

SF-36 er et spørgeskema som måler livskvalitet, og har vist sig værdifuldt som et prognostisk redskab ved iskæmisk hjertesygdom (Dempster and Donnelly, 2000;Nishiyama et al., 2005;Oldridge et al., 2002). Derudover har SF-36 været benyttet i store populationsstudier bl.a. til vurderingen af langsigtede sundhedsskadelige effekter fra arbejdsrelateret stress (Stansfeld et al.,1998). En sammenhæng imellem SF-36 og tidlig tilbagetrækning fra arbejdsmarkedet er også fundet (Harkonmaki et al., 2006).

WHO 5: Et studie har vist, at de hjertepatienter som har en lav score (< 50) på WHO 5 har en signifikant højere mortalitet inden for 6 år end patienter med en høj score (<50) (Birket-Smith et al., 2009).

1.4.3 Metaboliske variable og stress

Vedvarende stress er associeret til udvikling af det Metaboliske Syndrom (MES) (Folkow B 2001) (Rosmond R 2004). MES er en klynge af risikofaktorer for udvikling af hjerte-kar-sygdomme, herunder 1) nedsat glukose tolerance/diabetes type II, 2) abdominal fedtaflejring,

3) dyslipidæmi (forstyrret fedtstofskifte, og 4) hypertension (for højt blodtryk). Disse risikofaktorer betragtes som et væsentligt sundhedsproblem i den vestlige verden, og påvirker 40 millioner amerikanere.

Insulinresistens betragtes som en af de centrale faktorer i udviklingen af MES. Derudover peger et review af Sutherland et al. 2004, på, at MES er forbundet eller forårsaget af kronisk low-grade inflammation som følge af aktivering af immunsystemet. Kronisk low-grade inflammation er sandsynligvis også en relevant faktor i udviklingen af åreforkalkningssygdom i hjertet (Pradhan and Ridker, 2002). Insulinresistens, MES og kronisk low-grade inflammation er alle forbundet med endotel dysfunktion, som er kendetegnet ved, at blodkarrene bliver stivere og der sker ændringer i blodkarrenes struktur. Seematter et al. (2004), finder, at disse ændringer er forbundet med udviklingen af åreforkalkning.

1.4.4 Blodtryk som en markør til stress

Fedme, insulinresistens, forhøjet blodtryk og dyslipidermia (som alle ses i MES), er hver især risikofaktorer for hjerte-kar-sygdom og tidlig død (Farmer, 2004).

Den stigning i blodtrykket som ofte ses med en stigende alder, har vist sig at være relateret til vedvarende stress (Timio et al., 1997).

1.4.5 Heart-rate variabilitet og stress

Nedsat heart-rate variabilitet (HRV) har vist sig at være associeret til øget stress samt dårlig prognose for hjertesygdom. I studier med stress reducerende intervention har det vist sig, at HRV forbedres (Del Pozo et al., 2004;Nolan et al., 2008;Routledge et al., 2010).

1.4.6 Puls og Pressure-rate Product som stress markører

Hvilepuls har vist sig at have en prognostisk værdi ved iskæmisk hjertesygdom (Lanza et al., 2006), og koblingen har vist sig at være graden af vedvarende stress (Rogowski et al., 2007). Pressure-rate Product (PRP) er produktet af det systoliske blodtryk og pulsen, der bruges inden for kardiologien som et indeks for hjertets ilt optagelse og afspejler hermed hjertes arbejde. Hjertets arbejde er hovedsagelig kontrolleret af beta-adrenerge katekolaminer (Opie, 2001), hvilket gør PRP egnet som en indikator af den sympatiske tonus i hjertet (Noble, 2002) samt egnet som en prognostisk markør for iskæmisk hjertesygdom (Villella et al.,

1999).

Et tidligere studie har vist en signifikant sammenhæng mellem hvilepuls, PRP og PPS målet (Ballegaard et al 2009), dog har vi fravalgt at benytte Blodtryk, puls, tryk-rate-produkt og serum kolesterol som primære eller sekundære variabler i Songheart studiet, fordi studiets primære målgruppe får medicin der påvirker disse variabler.

1.5 PPS målet, stress og Diffuse Noxious Inhibitory Control System (DNIC)

Ændringer i DNIC systemets funktion kan være med til at forklare den forhøjede smerte følsomhed hos de kronisk stressede (overaktivt ascenderende stimulerende nervesignal i kombination med en svækket descenderende inhiberende nervesignal) (Arendt-Nielsen et al. 2010). Det danner også en plausibel hypotese for effekten af den daglige nervestimulation (akupressur), hvor patientens egen stimulation samt efterfølgende kontrol af at ømheden aftager, kan medføre en restitution af den tabte DNIC funktion, der ses hos den kronisk stressede.

1.6 Effekten af intervention der bruger PPS som biofeedback markør for stress:

Brugen af PPS som en bio-feedback markør i kombination med et multifacet stress-håndteringsprogram er blevet valideret i to blok-randomiserede kontrollerede forsøg; Et mindre på 50 opera sang elever (Ballegaard et al., 2011) samt et større på 292 kontoransatte (Ballegaard et al., 2011).

I det første studie blev 50 opera sang elever fra to forskellige skoler randomiseret til enten aktiv behandling eller kontrol behandling. Den aktive behandling svarer til den behandling, der anvendes i nærværende studie, mens kontrolbehandlingen var en times foredrag i generel stress håndtering. I forhold til kontrolgruppen opnåede aktiv gruppen et fald i puls (14%) et fald i hjertets arbejde målt som systolisk blodtryk x puls produkt (PRP)(14 %), et fald i antallet af kliniske stress tegn (67 %), i PPS (35 %) samt i det samlede antal af forhøjede helbredsmæssige risiko markører (60 %)(alle $p < 0.05$) (Ballegaard et al., 2011a).

I det efterfølgende større studie på 292 kontormedarbejdere, blev deltagerne grupperet i to grupper baseret på en PPS måling; meget stressede (PPS ≥ 60) og lav til moderat stressede (PPS <60). De meget stressede blev blok-randomiseret til enten aktiv behandling hvor de

modtog et PPS måleinstrument + en personlig guide til programmet i en 3 måneders periode, eller til kontrol gruppe. Resultaterne viste, at når den aktive gruppe blev sammenlignet med kontrolgruppen, havde interventionen en klinisk nyttig og videnskabelig signifikant effekt på blodtryk (10 %), hjertets arbejde (10 %), serum kolesterol (13 %) og serum LDL kolesterol (16 %) samt PPS (42 %) (alle p <0,001) og med en 80 % responsrate (Ballegaard et al., 2011).

I en prospektiv ukontrolleret undersøgelse på en tilfældig udvalgt gruppe af kvinder med brystkræft viste effekten af interventionen lignende resultater på PPS (Axelsson et al., 2011). Den gennemsnitlige ændring var fra 78 til 56 i PPS mål, sammenlignet med en gennemsnitlig ændring fra 74 til 49 blandt de 60 kontoransatte i aktiv gruppe i før omtalte undersøgelse (Ballegaard et al., 2011). Disse kvinder oplevede også en signifikant forbedring i niveau af angst og depression, og forbedringen i PPS var koblet til forbedringen i graden af depression. Desuden var gennemførlighed høj, da mere end 90 % af kvinderne stadig brugte programmet dagligt 6 måneder efter start(Axelsson A et al., 2010).

En 10-årig prospektiv, ukontrolleret, klinisk case-control database undersøgelse med 160 patienter med iskæmisk hjertesygdom (alle var kandidater til invasiv behandling), og som brugte finger palperet ømhed på brystbenet som en biofeedback mål for PPS i kombination med et omfattende egenomsorgsbaseret stress management program, viste, at interventionen havde langvarig gavnlig effekt på dødelighed samt på udgifter til sundhedsydelser (Ballegaard et al., 2004). Den 3-årige risiko for at dø var signifikant lavere end den generelle danske befolknings, med et betydeligt fald i brug af medicin, antal indlæggelsesdage på hospital, samt faldende behov for invasiv kardiologisk behandling, hvilket samlet set førte til ca. 70.000 DKK reduktion af de årlige sundhedsudgifter per patient (baseret på omkostningerne for 10 år siden). Disse patienter startede med 3 ugers akupunktur behandling, mens egenomsorgsdelen var den vigtigste indsats for resten af observationsperioden (Ballegaard et al., 2004;Ballegaard et al., 1999;Ballegaard et al., 1996).
I samme undersøgelsesserie fandtes, med hensyn til effekt på depression og livskvalitet, at der ved base-line var en signifikant sammenhæng mellem graden af depression og graden af sygdom målt via NYHA-klassifikation, og at graden af depression ved baseline desuden korrelerede selvstændigt til effekten målt som ændring i NYHA klassifikationen. Interventionen viste sig, at have en signifikant effekt på livskvaliteten og ydeevnen målt ved NYHA -klassifikation (Ballegaard et al., 1999).

Denne formodede gavnlige effekt mht. forbedret overlevelse, når stress håndtering anvendes af patienter med iskæmisk hjertesygdom, er for nylig blevet bekræftet i randomiserede undersøgelser (Gulliksson et al., 2011;Orth-Gomer et al., 2009). Andre forskere har vist, at sensorisk stimulation via akupunktur forbedrer ydeevne og HRV hos patienter med hjertesygdom (Kristen et al., 2010).
Hos patienter med slagtilfælde har det samme program, evalueret i en prospektiv, ukontrolleret, klinisk case-control database undersøgelse, vist sig at reducere den akkumulerede 4 års dødelighed til et niveau, der svarer til den generelle danske befolknings (Magnusson et al., 2010), mod en forventet overdødelighed på 50%. Halvdelen af dødsfald efter apopleksi kan tilskrives iskæmisk hjertesygdom (Bronnum-Hansen et al., 2001).

Konklusion med hensyn til en effekt af den valgte aktive intervention:

- PPS målet har vist sig at være koblet til fysiologisk stress, hjertets arbejde, graden af depression samt livskvalitet..
- To langtids-prospektive database studier viser markant effekt på forbrug af sundhedsydelser og overlevelse uden risiko for patienten. En leder artikel i det ansete tidsskrift JAMA konkluderer, at når effekten er stor og risikoen lille, er prospektive observationsstudier tilstrækkelige som baggrund for at indføre kliniske anbefalinger (Radford and Foody, 2001).
- Psykologiske spørgeskemaer viser, at patienterne i disse observationsstudier ikke adskiller sig fra hjertepatienter i medicinsk behandling, hvad angår psykologiske forhold.
- Der er observeret effekt i form af stress reduktion i uselekterede patienter (med Brystkræft).
- Der er i to prospektive randomiserede undersøgelser hos erhvervsaktive personer i arbejde vist effekt på stress niveauet og på centrale hjerte- og helbreds-risiko markører.
- Behandlingen har i samtlige undersøgelser vist sig harmløs.
- Når interventionen således har vist sin stress reducerende værdi i ikke randomiserede studier for patienter med livstruende sygdom som hjertesygdom, apopleksi og brystkræft, og efterfølgende i randomiserede studier for den almindelige befolkning i arbejde, er det naturlige næste skridt at undersøge om effekten er stærk nok til også at vise sig stress reducerende i et randomiseret studie hos én af de grupper af patienter med en livstruende sygdom, hvor interventionen allerede har vist sig virksom – her

iskæmisk hjertesygdom. Viser virkningen sig her at være kraftig nok til at kunne bekræfte de tidligere resultater, nu i et randomiseret studie, er virkningen forventeligt også kraftig nok til at kunne reducere stress belastningen hos patienter med andre livstruende sygdomme – og hos patienter med kroniske ikke-livstruende sygdomme.

1.7 Effekten af den anvendte hjerterehabilitering

De anvendte hjerterehabilitering programmer viser ingen effekt på de kardielle livsstils risikofaktorer og tjener hovedsageligt til at opretholde compliance i forhold til den medicinske behandling (Dansk Cardiologisk Selskab, 2010).

2. Hypotese

På ovenstående baggrund finder vi det relevant, at undersøge effekten af den aktive intervention i et prospektivt randomiseret design hos patienter, der har gennemgået et hjerte rehabiliteringsprogram, for der igennem at belyse dens eventuelle værdi, såfremt interventionen blev givet til danske hjertepatienter som et tillæg til den eksisterende rehabilitering.

Hypotese: Kan et simpelt, individualiseret og egenomsorgsbaseret interventions program, der bruger hyppig PPS måling som en feedback markør for stress, efterfulgt af nerve stimulation med henblik på at reducere stress niveauet, med heraf forventet efterfølgende øget personlig empowerment, forbedre patienternes grad af stress, depression, livskvalitet (QOL) samt en række faktorer af betydning for forværring af hjertelidelsen, med potentiel gunstig betydning for forbruget af sundhedsydelser samt langtidsprognosen?

Det er internationalt anerkendt, at der er en kobling mellem stress og depression (Grippo and Johnson, 2009). Ved at inddrage depressions målet som en måde at måle stress reduktion på, kobles det nye stress mål (PPS målet) til et kendt mål (depression). I forlængelse heraf er det også anerkendt, at depression har primær og sekundær negativ prognostisk betydning for hjertesygdom, samt at reduktion i depressions symptomer har en gunstig indflydelse på patienternes prognose mht. ”hårde” endepunkter, det vil sige hospitals indlæggelser, operationsbehov, tilbagefald og død. Tilsvarende gælder for livskvalitet målt ved WHO-5.

Det vil også være interessant såfremt det forholder sig sådan, at de patienter, der tager den nye teknologi til sig, får en klinisk effekt, selvom en sådan effekt ikke kan vise sig for aktiv-gruppen som helhed. Derfor er som sekundær effekt variabel anvendt korrelationen mellem ændringen i PPS og ændringen i MDI og WHO-5, således at forstå, at det ud fra et kardiologisk synspunkt vil være fuldt tilfredsstillende hvis undersøgelsen viser, at de hjertepatienter, der får et bedre PPS også opnår en forbedret livskvalitet, såfremt deres livskvalitet er nedsat ved baseline.

3. Formål

Undersøgelsen har to hovedformål:

1) Ud fra data fra baseline undersøgelsen:
   - Er der en sammenhæng mellem PPS målet og kronisk stress målt som Quality of life, forekomst af depression, hjerte rytme variabilitet, hjertet arbejde hos patienter med iskæmisk hjertesygdom
   - Er det en sammenhæng mellem PPS og kronisk stress vurderet som forløber for metabolisk syndrom. Metabolisk syndrom vurderes her primært ud fra nedsat insulin resistens/forstyrret sukkerstofskifte
   - Er der en sammenhæng mellem PPS og kronisk stress målt som ændret kropsfedt fordeling, dvs ændret forhold mellem android og gynoid fedt fordeling.

2) Ud fra data fra interventionsundersøgelsen:
   - At undersøge om to centrale elementer i omtalte egenomsorgsbaserede interventionsprogram (Ull Care programmet), PPS måling som bio-feedback markør for stress samt nerve stimulation for stress reduktion, kan hjælpe patienter med iskæmisk hjertesygdom, via øget egenomsorg, til:
     - reduktion i kronisk stress målt over en 3 måneders periode:
       - som reduktion i depression og forbedret livskvalitet
       - reduktion i stress målet PPS
       - gavnlig påvirkning af udvalgte kardiovaskulære risikofaktorer, f.eks. hjertets arbejde, HRV, lipider.
     - over en 12 måneders periode tillige føre til:

- reduktion i forbrug af sundhedsydelser
- en positiv effekt på patientens sygdomsprognose

3) Der laves særskilte understudier for følgende forhold
   a. Som følge af den anvendte randomiseringsprocedure opstår der mulighed for at lave en randomiseret prospektiv interventionsundersøgelse på de undergrupper, der opstår som følge af de anvendte kriterier (se afsnit 8 for detaljer). Det drejer sig om: alder, køn, tilstedeværelse af sukkersyge, tilstedeværelse af hjerteinsufficiens, samt varighed fra sidste myokardieinfarkt eller invasive behandling. Graden af depression indgår i hovedformålet under punkt 2.
   b. Sammenligne udfaldet af den valgte PPS måling med en kendt algometer måling på skinnebenet hos en tilfældig udvalgt gruppe af patienterne i forbindelse med den afsluttende kliniske efterkontrol efter 3 måneder.

4. Design

Prospektivt, randomiseret, kontrolleret, enkelt-blindet studie, med 50% allokations ratio for intervention. Tidsforløbet for interventions og kontrolgruppe er parallelt.
Undersøgelsen foregår på Herlev Hospital og er åben for følgende: deltagende patienter og instruktør af aktivgruppen. Undersøgelsen er blindet for alle andre, inklusiv de statistikere der skal lave hovedanalyserne. Det personale, der gennemfører de kliniske undersøgelser har således ikke adgang til dokumenter, der indeholder oplysninger om behandling eller behandlingsprocedurer. Dette gøres for at forebygge bias i sammenligningen mellem behandlingsgrupperne.

5. Materiale

5.1 Forsøgspersoner:

I undersøgelsen indgår 300 patienter med iskæmisk hjertesygdom. Patienterne vil blive rekrutteret via center for hjerterehabilitering på hhv. Gentofte og Herlev hospitaler.

5.2 Inklusionskriterier:

- Gennemført hjerterehabiliterings program på Gentofte eller Herlev Hospital for minimum 6 måneder siden.

- Dokumenteret iskæmisk hjertesygdom som har kvalificeret til hjerterehabiliterings forløb.
- Brug af aspirin, kolesterolsænkende og blodtrykssænkende medicin efter danske kardiologiske retningslinjer.
- Yngre end 75 år gamle på inklusionstidspunktet.
- Forhøjet niveau af stress, defineret som PPS ≥ 60.

5.3 Eksklusionskriterier:

- Tidligere diagnosticeret og behandlet for psykiatriske lidelse, undtaget er depression.
- En kronisk konkurrerende lidelse, der statistisk set er livs forkortende (som fremskreden kræft med metastaser).
- En kronisk konkurrerende lidelse, som klart forringer patientens livskvalitet, og som ikke er hverken hjertesygdom eller diabetes (f.eks. KOL; Kræft; kronisk smertesyndrom).
- Patienter, som ikke kan klare sig selv.
- Patienter, der af forskellige årsager vil have svært ved at deltage i interventions-programmet, eks. sprogbarrierer.
- Planlagt hjerteoperation.
- Tidligere behandling med den undersøgte aktive behandling.
- Fået ændret sin hjertemedicin (hvad angår angina pectoris og/eller hjertesvigt) inden for det sidste måned.

NB: Patienterne kan deltage i andre kliniske forsøg på samme tid, da dette ikke er et udelukkelseskriterium.

6. Effekt variable (interventions studiet)

For baseline undersøgelse tages supplerende blodprøver i forhold til hvad der er nævnt i punkt 6.3.1. neden for (se punkt 9.3)

6.1 Primær effekt variabel:

Graden af depression ved spørgeskemaet MDI (Bech et al., 2001;Olsen et al., 2003)

6.2 Sekundære effekt variable:

1. Fysiologisk måling af stress: Niveau af stress målt ved PPS og foretaget af den samme blindede sygeplejerske (uddannet i PPS måling) ved baseline og efter 3 måneders observation.
2. WHO-5 spørgeskemaet (Folker and Folker, 2008).
3. Korrelation mellem ændringer i PPS og ændringer i henholdsvis MDI og WHO-5.

6.3 Tertiære effekt variable:

6.3.1 Biologiske variable:

1. Mål for vedvarende stress: serum Oxytocin.
2. Insulinresistens: Fastende plasma insulin, C-peptid, blod glukose, (med beregning af HOMA), HbA1c, Adiponektin.
3. Kronisk inflammation: Serum / plasma high sensitive CRP, Defensin, YKL-40.
4. Fedt stofskiftet: total kolesterol, LDL- og HDL kolesterol, Triglycerid.
5. Hjertets belastning NT-proBNP og BNP.

6.3.2 Fysiologiske variable:

1. Blodtryk, puls, blodtryk-puls produkt (PRP).
2. Body Mass Index, (højde og vægt).
3. Kropsfedtsammensætningen målt ved DEXA scanning.
4. Hjerte rytme variabilitet (HRV) målt ved vippetest.

6.3.3 Generel trivsel

1. Målt ved hjælp af spørgeskemaet SF-36 (Alonso et al., 2004;Bjorner et al., 1998).

6.3.4 Sundhed økonomi (cost / benefit) analyse

Data fra de sidste 12 måneder forud for inklusionstidspunktet vil blive brugt som baseline variable. Udføres kun såfremt de primære og sekundære effekt mål opfylder kriterierne for succes (se punkt 12). Data vil blive indsamlet for følgende ydelser:

1. Antal besøg hos praktiserende læge (for hjerteproblemer)

2. Antal besøg hos en kardiolog (specialist)
3. Antal hospitalsbesøg (ambulant)
4. Antal indlæggelsesdage på hospital
5. Brug af invasive procedurer (PCI og/eller CABG)
6. Forbrug af hjertemedicin
7. Antal sygedage pga. hjertesygdom (såfremt personen er i arbejde)

6.3.5 Andre variable:

1. Hyppighed af klinisk begivenhed: måles som et sammensat endepunkt når én af følgende begivenheder indtræffer: 1) besøg på skadestuen for en kardiologisk grund; 2) indlæggelse på hospital af kardiologisk årsag og 3) hjertedød. Besøgene hos den praktiserende læge vil ikke indgå i regnskabet, da disse besøg vil blive opfattet som også at omfatte andre end patientens hjerte sygdom (fx halsbetændelse/influenza).

2. Patient compliance: vurderes ud fra 1) antal patienter, der følger programmet til udgangen af forsøgsperioden 2) personlige opfattelse af nytte af programmet på en 7 punkt ordinal skala, og 3) overholdelse af Ull Care programmet målt som graden for integration i det daglige liv på en binomial skala.
3. Antallet af kliniske stress tegn vurderet ud fra et konstrueret og valideret spørgeskema (Ballegaard et al., 2011).
4. Ændring i medicinering, for at sikre, at alle observerede forbedringer ikke skyldes en stigning i medicinering ordineret af læger uden for forsøgsprotokollen.
5. Brug af invasive procedurer (PCI og CABG).
6. I forbindelse med understudiet, hvor PPS sammenlignes med en kendt algometri måling, bliver de to algometri målingerne lavet i randomiseret rækkefølge på skinnebenet på 50 tilfældigt udvalgte patienter i forbindelse med den kliniske efterkontrol.

7. Praktisk forløb (inkl. flowdiagram)

- Via databaser over hjerterehabiliteringspatienter på hhv. Gentofte og Herlev Hospital identificeres ca. 600 patienter med afsluttet hjerterehabiliterings forløb > 6 mdr. siden, for at sikre en stabil tilstand med hensyn til effekten af den givne hjerterehabilitering. Patienterne inviteres i brev til at deltage i undersøgelsen.

- Det forventes at ca. 450 siger ja tak. Disse indkaldes til undersøgelse på Herlev Hospital medicinsk afdeling O.
- Alle underskriver ”samtykke erklæring” og gennemgår den fysiologiske forundersøgelse.
- Patienter med PPS < 60 afsluttes umiddelbart, dog således at en undergruppe på de første 50 patienter med PPS < 45 inviteres til en måling af kropssammensætning.
- Idet det forventes, at 2 / 3 af patienterne har en forhøjet PPS niveau (≥ 60), vil 300 patienter med PPS ≥ 60 bliver randomiseret til aktiv eller kontrol gruppe, 150 i hver gruppe:
    - **Kontrol gruppe:** Fortsat egen indsats i forlængelse af den allerede givne hjerte-rehabilitering, samt udlevering af en undervisningsbog om generel stresshåndtering (REHAB + CARE).
    - **Aktiv gruppe:** Samme, samt Ull behandlingsprogram (REHAB + ULL).
- Alle patienter vil ved baseline få tilbudt en supplerende måling af kropssammensætning.
- Projekt periode: 3 mdr:
    - Både kontrol og aktiv gruppe fortsætter med den givne medicinske behandling. Hvis der opstår en ændring i den hjertemæssige tilstand herunder mulige medicinbivirkninger, tilpasses medicinen efter procedurerne i Rehabiliteringsprogrammerne på det hospital hvor patienten er tilknyttet (Gentofte eller Herlev Hospital). Hvis det er muligt, forbliver medicinen stabil under hele studiets periode på 3 måneder. Patient og patientens egen læge informeres om, at det tilstræbes i studie perioden, at al justering af hjertemedicin sker efter kontakt til projekt sygeplejersken (evt. via telefon hot-line).
    - Kontrol/Aktiv gruppe:
        1. Kontrol gruppe: får bog om generel stresshåndtering
        2. Aktiv gruppe: der foretages i tillæg:
            - Instruktion i selv måling af PPS ved hjemmebesøg af PPS instruktør. Her instrueres kun i PPS måling, nerve stimulation samt brug af webplatform med henblik på at indtaste egne PPS målinger og ikke i stress håndtering. Der foretages et hjemmebesøg af 45 minutters varighed ved start, samt 2 besøg efter henholdsvis 1 og 2 måneder med henblik på supplerende

teknisk instruktion såfremt PPS målet ≥ 60. Derudover er der 5 telefonsamtaler á 15 minutters varighed, alene med henblik på teknisk instruktion, efter henholdsvis uge 1, 3, 5, 6 og 10. Patienten får udleveret et Ull Meter, en skriftlig bruger manual, samt en instruktions DVD.

- Patienterne registrerer deres PPS målinger, enten på web baseret platform (www.songheart.org) eller på papir, mindst én gang om ugen.
- Såfremt deres PPS registreringer viser, at PPS≥ 60 i 7 konsekutive morgen målinger, kontaktes patienten med henblik på behov for supplerende teknisk instruktion.

- Efter 3 måneders deltagelse i projektet indkaldes alle patienter (begge grupper) til gentagelse af base-line besøg, på Herlev Hospital. For de personer, der ved baseline valgte at få lavet kropssammensætning vil dette tilbud blive gentaget for de personer, der ved den fysiske undersøgelse viser sig at have tabt 3 kilo eller mere.
- Patienterne i aktiv gruppen kan fortsætte med at bruge PPS instrument såvel som webplatform til indtastning af egne målinger i samtlige 12 måneder.
- De 2 undersøgelse på Herlev foretages af den samme, blindede sygeplejerske ved henholdsvis baseline og 3 måneders opfølgning.
- Hvis succeskriterierne for nærværende undersøgelse er opfyldt, vil de efterfølgende analyser foretages efter 12 måneders observation:
    - Brug af sundhedstjenester i løbet af de sidste 12 måneder før starten på deltagelse af undersøgelsen, vil blive sammenholdt med de 12 måneder efter påbegyndelsen af deltagelse
    - Hyppigheden af kliniske begivenheder: Her vil blive anvendt Kaplan-Meier plot analyse. Den mindste relevante kliniske forskel mellem aktiv og kontrolgruppe (MID) er fastsat til 10 % forbedring i den aktive gruppe i forhold til kontrolgruppen.

7.1 Flowdiagram:

Besøg (nummer)	1	2	3	4
Fysisk placering	Herlev	hjemme besøg	Herlev	Dataindsamling via offentlige registre
Måneder	0	0	3	12
Vindue for besøg		+/-7 dage	+/-7 dage	
Informeret samtykke	X			
Spørgeskemaer udfyldes	X		X	
Inklusion/eksklusion	X			
Blodtryk, puls, PPS og HRV test	X		X	
Blodprøve	X		X	
Vurdering af medicin	X		X	
Randomisering	X			
Registrering af kliniske begivenheder	X			X
Forbrug af sundhedsydelser	X			X
Implementering af fælles behandling	X			
Implementering af aktiv behandling		X		
Måling af kropssammensætning (undergruppe)	X		X	

CONSORT Flow Diagram for SongHeart studiet

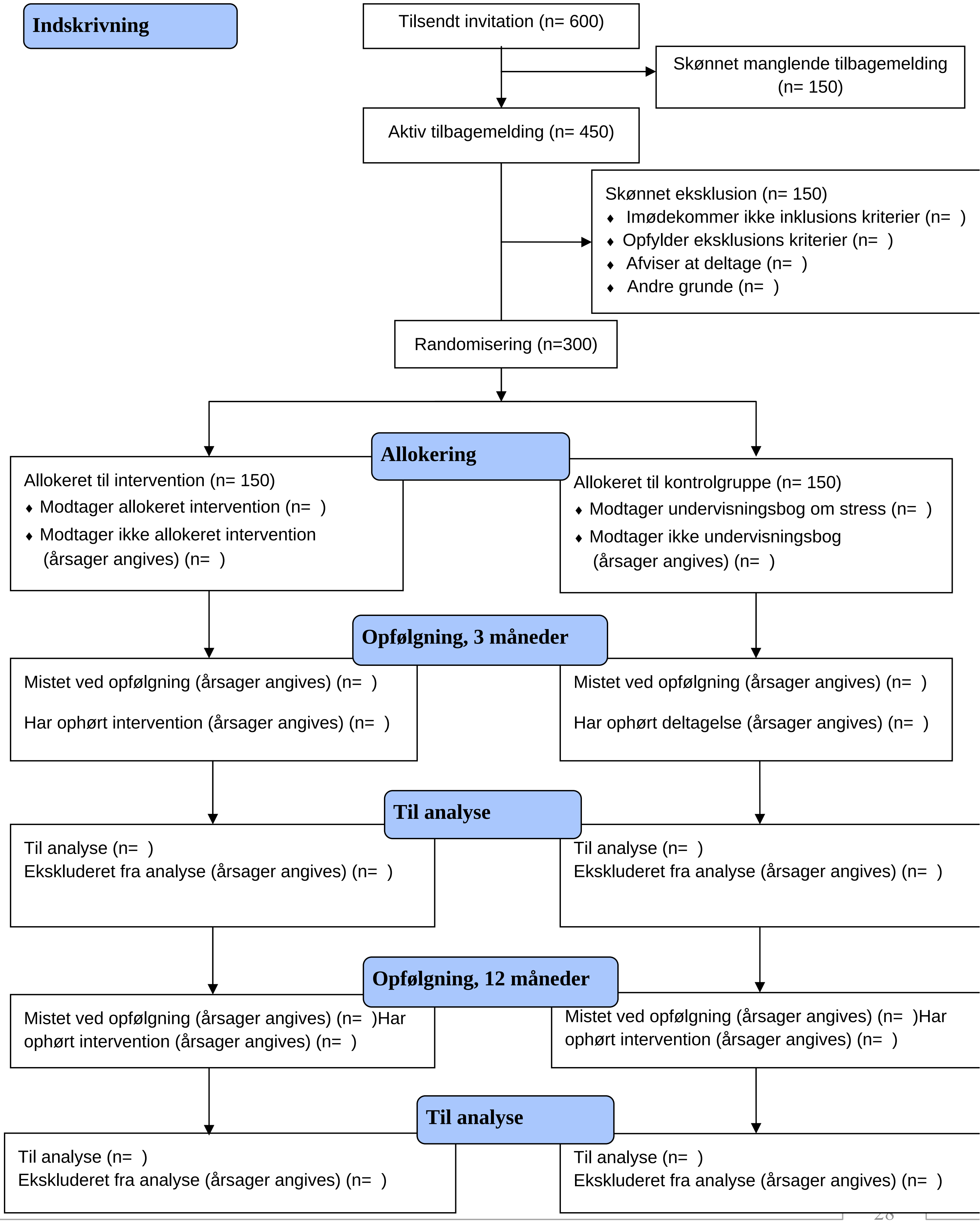

8. Randomiserings procedure

Behandlingen tildeles randomiseret, med lige mange patienter i kontrol gruppe og til aktiv intervention. Den praktiske randomisering foretages elektronisk af en uvildig forskningsvirksomhed (www.larix.dk). Resultatet af randomiseringen er blindet for de undersøgende sygeplejersker og læger og andre der er ansat til at deltager i studiet, herunder også de statistikere, der efterfølgende analyser data i forhold til den videnskabelige hovedartikel. Undtaget blinding er de personer, der forestår undervisning af Aktivgruppen samt patienterne.

Behandlingen tildeles ved en minimerings procedure med indbygget tilfældighed (Pocock and Simon, 1975). Minimering vil sikre tilnærmet balance i antallet af patienter i de to behandlingsgrupper der har hhv. lave og høje værdier af de baseline parametre som der tages højde for i proceduren. Pococks og Simons minimeringsmetode (Pocock and Simon, 1975) vil blive brugt med en tilfældighedsparameter på 0.8. Følgende baseline parametre vil indgå:

- Alder (< 65, ≥ 65 år)
- Køn (Mand, Kvinde)
- Hjerteinsufficiens (Ja, Nej)
- Diabetes (Ja, Nej)
- MDI (score <15, ≥ 15)

Et uafhængigt forskningsselskab udfører randomiseringen elektronisk (www.larix.dk).Behandlings allokeringen vil blive blindet i forhold til de undersøgende sygeplejersker, men ikke i forhold til patienten og de fag personer, der skal yde den aktive behandling.

9. Metoder

9.1 Stressmåling:

Der anvendes et Ull Meter, som er et instrument udviklet til at måle sensibiliteten/aktiviteten i det polymodale nervesystem svarende til det mest ømme punkt på sternum mellem costa 3-5. Punktet identificeres ved tryk med finger. Forinden lærer patienten sin smertetærskel at

kende ved at instruktøren med instrumentet applicerer et gradvist stigende tryk på skinnebenet, og patienten siger stop, når tærskel for smerte/ubehag nås. Herefter gentages proceduren på det mest ømme sted på brystbenet, hvor patienten selv applicerer tryk og stopper når smertetærskel nås. Instrumentet viser derefter et tal på en skala fra 30 til 100, hvor en øget sensibilitet medfølges af et stigende tal, PPS; det vil sige, at et højt PPS er udtryk for højt stressniveau (= høj følsomhed)(Ballegaard et al., 2009).

Ull Meteret har følgende specielle funktioner:

- Skjult måleværdi indtil målingen er gennemført.
- En beregning af sensitiviteten til en ømhedsscore på en logaritmisk skala lignende den decibel scala der benyttes for at estimere grænseværdierne for lydtryk i forbindelse med høretest.
- En alarm der aktiveres ved et tryk, før opståen af eventuelle skade.
- En særlig fodpude som forhindrer irritation af huden, for at sikre at det er tryk sensitiviteten på knoglen der måles og ikke hudens sensitivitet.

9.2. Behandlingsmodaliteter:

9.2.1 Nuværende hjerterehabilitering:

Alle patienter har gennemgået og deltager i den hjerterehabilitering, som er defineret af de respektive kardiologiske afdelinger i overensstemmelse med de offentlige sundhedsregler (The National Board of Health, 2004;The network group of "heart rehabilitation", 2004).

9.2.2 Ull Care programmet:

Grundelementerne af interventionen er som følger:

1) En egenomsorgsdel.
2) En professionel instruktion i PPS måling, kognitiv refleksion i forhold til målet samt nerve stimulation.
3) Løbende registrering af deltagernes PPS mål via Webjournal.

Det egenomsorgsbaserede interventionsprogram er et stresshåndteringsprogram baseret på brugen af PPS målet som en bio-feedback markør for stress. Den vigtigste indsats er den

daglige egenomsorg i form af PPS måling, kognitiv refleksion og nerve stimulation, koblet til professionel teknisk hjælp afhængig af behov.

Elementerne af egenomsorgs programmet

- Individuel opstart bestående af en 45 minutters ansigt-til-ansigt hjemmekonsultation med en Ull Care instruktør, der instruerer i brug af instrument, fortolkning af PPS målingen, nerve stimulation og web platform.
- Et personligt Ull Meter instrument.
- En personlig webjournal til track recording af deltagerens PPS måls udvikling (patienter uden internetadgang modtaget printede registrerings-ark).
- Et hæfte der beskriver egenomsorgsprogrammet i detaljer.
- 2 supplerende ansigt-til-ansigt møder efter henholdsvis 1 og 2 måneder mhp. supplerende teknisk instruktion, såfremt PPS målet forbliver ≥ 60.
- 5 x 15 minutters telefonkonsultationer efter henholdsvis 1, 3, 5, 6, og 10 uger. Kun de følgende fire emner omtales: PPS målet, fortolkning af målene, nerve stimulation og brug af webjournalen. Dette er for at sikre korrekt brug af Ull Meter instrumentet og korrekt forvaltning af nerve stimulationsteknikken.
- Online vurdering af deltagerens PPS mål. Denne vurdering bliver automatisk foretaget på hjemmesiden. Hvis PPS målet har været≥60 i mere end 7 dage i træk, bliver deltageren tilbudt supplerende teknisk instruktion.

De daglige trin i egenomsorgsprogrammet

Fire elementer udgør det daglige egenomsorgssystem: måling, kognitiv refleksion, handling og kontrol.

Måling: PPS måles to gange dagligt som et fuldt privat mål og indskrives i en personlig webjournal, således at personen løbende kan følge sin egen udvikling, samtidig med at der også er løbende ekstern faglig kontrol.

Refleksion: Kognitiv refleksion som reaktion på det faktiske PPS mål og deres udvikling.

Handling: Nerve stimulation på udvalgte akupunktur punkter svarende til instruktion. Eventuel yderligere egen indsats som nævnt i bogen vedrørende generel håndtering af stress, der gives til alle deltagere (både aktiv- og kontrolgruppen). PPS målet bliver brugt som

personlig feedback markør for stress, hvor der tages hensyn til virkningen af deltagerens valgte indsats.
Kontrol: Mål effekten af hvilken som helst handling/manglende handling på hvilket som helst tidspunkt.

Denne fire-trins procedure tilstræbes mindst to gange dagligt (morgen og aften) for at være forbyggende; det anbefales at udføre proceduren morgen og aften med henblik på at fremme en opbygning af en betinget refleks. Denne refleks (i) letter vanen og (ii) forbedrer effekten af handlingen, hvilket er vigtigt for ad hoc-brug, hvis deltageren skulle ønske at nedsætte sit stressniveau akut elle får ondt i brystet. Det er samtidig vigtigt at programmet ikke opfattes som en sur pligt, hvilket understreges over for patienten.

De specifikke terapeutiske elementer i programmet:

Bio-feedback måling af stress, ved brug af PPS målet som målet for i stress og i hvilket omfang patientens hjerte er påvirket af stress efterfulgt af:

- Sensorisk nerve stimulation (akupressur/shiatsu massage)

Patienten er instrueret i at udføre forebyggende nerve stimulation to gange dagligt + ad hoc når vedkommende akut ønsker at reducere stress. Nerve stimulation udføres ved, at man med en finger påfører et tryk i et minut, der er tilstrækkeligt til at man kan mærke det, men uden at det forårsager smerte. Dette gøres på følgende akupunktur punkter: på brystbenet på niveau med den 4. interkostale plads (Shanzhong, C.V. 17), og på ryggen 3,8 cm lateralt for den spinale proces i 4. og 5. brysthvirvel (Jueyinshu and Xinshu, U.B. 14 and 15). Nerve stimulationen er udført succesfuldt, når patienten efter at have trykket kan observere, at ømheden på punktet er faldet. Hvis dette resultat ikke er opnået, bliver patienten instrueret i at gentage behandlingen. Den gentagne nerve stimulation har to primære formål: 1) at genskabe nervesystemets adaptationsevne via den gentagne stimulering af det diffuse smertehæmmende kontrol (DNIC) system (Arendt-Nielsen L and Yarnitskyy D, 2010) og 2) at reducere et forhøjet stress niveau via den gentagne ikke-smertefulde sensoriske stimulation

af den polymodale nervecelle, der medfører en frigivelse af hormonet oxytocin (Uvnãs-Moberg, 1997a). Hvis muligt bliver ægtefælle eller samlever instrueret i at udføre nerve stimulation på ryggen, hvilket uafhængigt kan bidrage til stresslindrende effekt gennem en særskilt oxytocin frigivelse forårsaget af den udviste omsorg (Carter et al., 2007;Grewen et al., 2005;Light et al., 2005;Uvnãs-Moberg, 1997b). Hvis en ægtefælle eller samlever ikke er tilgængelig, kan behandlingen udføres af personen selv ved hjælp af bold (en teknik personen modtager instruktion i).

9.2.3 Fælles intervention for både aktiv og kontrol gruppe:

Alle deltagende patienter får udleveret en bog om generel stresshåndtering ("Stress, Lægens Bord" Netterstrøm, Bo, DR, 2002).

9.3 Blodprøve udtagning og analyse:

Blodprøver tages lokalt på Herlev Hospital hvor de opbevares ved -80 grader indtil analyse. Efter analyse, og senest 1. juni 2013 destrueres rest materiale.

Alle blodprøve analyser vil blive udført på Medicinsk Afdeling O, Endokrinologisk Enhed, Forskningslaboratorium 54O4, Herlev Hospital, enten som rutine eller forsknings analyse via kommercielle analyse-kits. Alle prøver analyseres i samme assay, for at undgå inter-assay variation, dvs. analyserne bliver først udført efter afslutning af studiet.

Følgende analyseres (baseline undersøgelse og interventionsundersøgelse tilsammen):

- Mål for vedvarende stress: HbA1c, serum Oxytocin.
- Mål for insulinresistens: Fastende serum / plasma insulin, C-peptid, blodglukose (med beregning af HOMA), HbA1c, Adiponektin.
- Mål for kronisk inflammation: Serum / plasma high sensitive CRP, Defensin, YKL-40.
- Mål for fedstofskiftet: total kolesterol, HDL- og LDL kolesterol samt Triglycerid.
- Mål for hjertets belastning: NT-proBNP og BNP.

Ud over dette tages et reserveglas på 5 ml blod til evt. senere brug ved såvel baseline som ved 3 måneders kontrol. Disse reserveglas opbevares i kodet form (uden CPR nummer) i en af

Datatilsynet og Region Hovedstaden godkendt biobank (database id: 30122009.HEH.O.JF). Denne database er en fortløbende database uden påtænkt afslutningstidspunkt

9.4 Kropssammensætning:

Udføres ved brug af helkrops-DEXA scanning (apparatur: Hologic) med bestemmelse af kropssammensætning med hensyn til fedt-, muskel- og knoglemasse. Endvidere kan det viscerale fedt beregnes via et nyt software, dvs man skelner mellem android og gynoid fedt. Der anvendes en velkalibreret røntgenstråle til scanning af patienten.

9.5 HRV stillingstest:

Forsøgsdeltagerne får målt korttids-HRV (5-min) ved et EKG (hjerte eletrokardiogram) i liggende hvile. Hvilemålingen foretages med forsøgsdeltageren liggende på et vippeleje. Efter hvileperioden vippes forsøgsdeltagerne uden brug af deres egen muskelkraft til 70 grader (oprejst) kropsposition. EKG registreres i denne position. Forskellen mellem hvile og stående repræsenterer reaktiviteten i det autonome nervesystem. Lavere reaktivitet i det autonome nervesystem er forbundet med vedvarende stress. Måling af HRV er non-invasiv, og er ikke forbundet med ubehag eller nogen risiko for forsøgsdeltageren.

9.6 Algometrisk måling på skinneben med kendt algometer

Smertetærskel til tryk undersøges skinnebenet med henholdsvis den i forsøget anvendte PPS måler, samt et kendt algometer {Arendt-Nielsen, 2009). Undersøgelses proceduren er her en lille smule anderledes: Undersøgeren trykker instrument ind på patientens skinneben med at forudbestemt tryk, hvorefter patienten skal sige ”stop”, når tiden for trykket bliver så lang, at det opleves som ubehageligt. Stimulationen bliver påført af den undersøgende sygeplejerske og er således ikke forbundet med hverken risiko eller smerte, idet patienten jo netop skal sige ”stop” når grænsen for ubehag/smerte nås. Denne undersøgelse vil kun blive gennemført på 50 tilfældigt udvalgte personer i forbindelse med den afsluttende kliniske kontrol.

10. Tidsramme for studiet

- Første patient forventes at starte 3 måneder efter at de finansielle midler er tilvejebragt, samt efter godkendelse fra Den Videnskabsetiske Komité og Datatilsynet
- Inklusionsperioden ophører når det ønskede antal inkluderer patienter er nået (300).
- Behandlingsperioden afsluttes 3 måneder efter at sidste patient er inkluderet.
- Den videnskabelige hovedartikel forventes færdig efter yderligere 3 måneder.
- Den samlede projektperiode er anslået til 1 år fra det tidspunkt hvor første patient inkluderes.

11. Statistik

11.1 Power og beregning af studie populations størrelse:

Den primære analyse er baseret på spørgeskemaet MDI depression score. Forekomsten af depression, målt som en MDI score ≥ 20, er fundet at være cirka 20 % hos patienter med iskæmisk hjertesygdom (Olsen et al 2003; Bech P 2001). I en undersøgelse blandt 292 kontorarbejdere fandtes hyppigheden af en MDI score at være dobbelt så stor blandt personer med PPS ≥ 60, sammenlignet med personer med PPS < 40 (Ballegaard et al 2011). På den baggrund skønnes det, at blandt patienter med iskæmisk hjertesygdom og et samtidigt forhøjet PPS, er hyppigheden af en MDI score ≥ 20 øget med 80 % i forhold til gruppen af patienter med iskæmisk hjertesygdom som helhed, det vil sige 36 %.

Den mindste relevante kliniske forskel (MID) er sat til 0,4 når effekten af aktiv behandling sammenlignes med kontrolbehandling. MID hvad angår forbedring i MDI score er sat til 5 point på MDI skalaen, når aktiv- sammenlignes med kontrolgruppe. Standardafvigelsen på forbedring i begge grupper er anslået til 9 enheder på MDI skalaen. Alfa er sat til 5 %, Beta til 20 %. På denne baggrund kræves der 50 patienter i hver gruppe med en MDI score ≥ 20, og dermed 300 patienter i alt, når der desuden inkluderes en 10 % sikkerhedsmargin for de skønnede værdier. En interim analyse på de første 60 patienter har vist, at de skønnede prevalenser for MDI score afviger fra de fundne. Derfor ændres cutpoint på MDI skalaen til MDI score = 15.

- Effekt størrelse beregnet ud fra alle inkluderede patienter (on intention to treat basis)
- Effekt størrelse er brøk, der består af forskellen i MDI score fra før intervention til efter behandling for aktivgruppe, minus den samme forskel for kontrolgruppen divideret med spredningen på ændringen i MDI score, når man tager alle patienter under et. En gennemgang af alle de 12 SSRI præparater, der af FDA er godkendt til behandling af depression, er den gennemsnitlige effekt størrelse 0.3 (Turner et al., 2008). Eksempel: MDI score i aktivgruppen ændrer sig fra 24-11 = 13; mens MDI score i kontrolgruppen ændrer sig fra 24-14 = 10). Denne forskel i MDI score er 13-10= 3; hvis spredningen (standard deviation) på MDI scoren samlet set er 7.5, vil effekt størrelse være 3/7,5 = 0,4 (Per Bech, 2010).
- Den mindste klinisk relevante effekt størrelsen for WHO – 5 beregnes på samme måde til 0,5 (Per Bech, 2010).

11.2 Analyse af effekt variable

Fem procent (5 %) vil blive brugt som signifikans niveau i alle statistiske tests.

De statistiske analyser vil se på to grupper af hjertepatienter hver for sig: 1) de patienter for hvem alle terapeutiske tiltag er udtømte og 2) resten. En samlet analyse vil blive foretaget såfremt det er statistisk set rimeligt. Statistik udarbejdes på ”intention to treat basis”; det vil sige at alle inkluderede patienter indgår i den efterfølgende analyse.

I den statistiske analyse vil der tillige laves subgruppe analyse for de patienter, der har de højeste PPS værdier: f.eks. alle med PPS ≥ 75; eller for den højeste kvartil af PPS.

“Closed test procedure” vil blive brugt i forhold til analyse af data efter henholdsvis 3 og 12 måneder hvad angår vurdering af behandlingseffekt:

- Første prioritets hypotese: Ull Care programmet forbedrer graden af depression målt med MDI depressions score, målt efter 3 måneders intervention
- Anden prioritets hypotese: Ull Care programmet forbedrer sundhedsøkonomiske variable, målt efter 12 måneders intervention.
- Tredje prioritets hypotese: Ull Care programmet forbedrer hyppigheden af kliniske begivenheder, målt efter 12 måneders intervention.

Baseline målinger vil blive co-variante variable i forhold til den anvendte stratificeringsvariabel: graden af depression målt med MDI skalaen. Data fra før og efter intervention vil blive analyseret særskilt. Delta værdier for henholdsvis aktiv- og kontrol-gruppe vil blive sammenlignet for at analyse en mulig behandlingseffekt. Alle sekundære og tertiære effekt variable vil blive analyseret efter intervention via varians og regressions analyser. Såfremt præmisserne for de foreslåede statistiske analyser ikke kan imødekommes, anvendes non-parametriske analyser. Quality-of-life data vil blive analyseret svarende til anvist for det enkelte spørgeskema. Alle effekt data vil blive præsenteret i en samlende tabel og primære, sekundære og tertiære effekt variable vil blive anført.

11.3. Cost-Utility analyse (CUA)

En cost-utility analyse vil blive gennemført efter 12 måneders intervention, til sammenligning af aktiv versus kontrol behandling. Relevante data, dvs. forbrug af sundhedsydelser i form af hospitalsbesøg, ambulatorie besøg, medicin forbrug, forbrug af invasive procedurer etc. vil blive indsamlet mens undersøgelsen pågår. Data fra SF36 spørgeskema vil blive brugt til at genere QALY's, baseret på, at den mindste relevante kliniske effekt på fysisk helbred (PCS skala) og psykisk helbred (MCS skala) er 0,3 (beregnet som vist i punkt 11.1)(Ware JE et al 2007)). Skøn for indirekte omkostninger, f.eks. sociale pensioner vil indgå.. CUA vil blive beregnet med såvel med som uden de indirekte omkostninger.

11.4. Supplerende undersøgelser

Det er hensigten at gentage Songheart studiet med en identisk design i Israel med det formål efterfølgende at kunne lægge data fra de to studier sammen og således øge den samlede statistisk styrke.

11.5 Analyse af understudier

Der vil blive lavet særskilt analyse svarende til de valgte understudier:

1) Vurdering af effekt af interventionen ift følgende undergrupper:¨

    a. Alder (< 65, ≥ 65 år)
    b. Køn (Mand, Kvinde)

c. Hjerteinsufficiens (Ja, Nej)

d. Diabetes (Ja, Nej)

e. Varighed siden sidste AMI/invasive indgreb: < 2 år, ≥ 2 år

12. Succeskriterier

Resultatet af undersøgelsen vil blive konkluderet som positivt, hvis følgende resultater nås:

- I forhold til kontrolgruppen, opnår den aktive gruppe en signifikant forbedring med hensyn til MDI depression score og med en terapeutisk gevinst på mindst 0,4 når virkningen i aktiv gruppe sammenlignes med kontrolgruppen.
- Når man sammenligner med kontrolgruppen, opnår den aktive gruppe en signifikant forbedring med hensyn til PPS målet målt af professionel instruktør, og med en MID på 10 enheder på PPS skalaen.
- Patienten påføres ikke en øget risiko i forbindelse med interventionen, målt som ingen stigning i blodtrykket og ej heller en stigning i serum kolesterol efter behandling. Begge dele er målt som gennemsnitsværdier (mean values) for de to variable i aktiv gruppen, når baseline værdier sammenlignes ved behandlingsperiodens afslutning.

13. Nytte af forsøget og behandlingen i et bredere perspektiv

Ved deltagelse i forsøget kan den enkelte patient få et generelt udbytte i form af muligheden af et supplerende omfattende helbredstjek. Såfremt resultatet af undersøgelsen falder positivt ud, betyder det, at patienterne i aktiv gruppe har fået et ekstra udbytte i form af mindre stress, bedre humør samt nogle konkrete værktøjer til at kunne styre fremtidigt stress bedre. Såfremt fornyede fondsansøgninger giver mulighed for det, vil kontrolgruppen da også få tilbudt den aktive behandling. For det omgivende samfund giver forsøget mulighed for at afprøve værdien af ny og økonomiske set billig behandling hos hjertepatienter, der forventeligt vil få målbar indflydelse på patienternes fremtidige sygelighed, brug af sundhedsydelser samt på længere sigt, patienternes overlevelse - idet andre videnskabelige undersøgelser har vist, at såfremt depressions- og stress niveau falder hos en hjertepatient, forbedres overlevelsen på lang sigt. Da interventionen repræsenterer et paradigme skift i behandling af kronisk syge, med hovedvægt langt på en øget egen indsigt og egen indsats, vil et positivt udfald forventeligt føre til, at interventionen vil blive testet hos mennesker med andre kroniske sygdomme.

Forsøget er tillige designet således, at det giver mulighed for at afdække mulige koblinger mellem vedvarende stress og hjertesygdom, samt hvilke virkningsmekanismer, der kan ligge til grund for den effekt, som et positivt udfald vil vise.

14. Etik

Undersøgelsen forudsættes godkendelse af videnskabsetisk komité. Efter godkendelse anmeldes forsøget til www.clinicaltrials.gov. Studiet anmeldes ligeledes til datatilsynet. Projektet overholder Helsinki Deklaration.

Undersøgelsen vil imødekomme de danske medicinske forskrifter og ICH retningslinjer for god klinisk praksis.

Alle forsøgspersoner informeres mundtligt og skriftligt om forsøget. Der indhentes informeret skriftligt samtykke af forsøgsansvarlige. Alle forsøgspersoner vil i det initiale brev med invitation til deltagelse få vedlagt pjecen ”dine rettigheder som forsøgsperson i et biomedicinsk forskningsprojekt”, en skriftlig ”Deltagerinformation” samt få tilbudt en personlig mundtlig informationssamtale.

Der vil blive lagt vægt på, at informationssamtalen drejer sig om en forespørgsel om deltagelse, og at forsøgspersonen har ret til at medbringe en bisidder til samtalen. Desuden vil forsøgspersonens ret til ikke at få oplysninger om helbredstilstand og disposition til sygdom blive gennemgået og respekteret.

Hvis der under afviklingen af forsøget fremkommer nye oplysninger om effekt, risici og bivirkninger, vil forsøgsdeltagerne blive informeret og der indhentes evt. fornyet samtykke.

Såfremt forsøgspersonen giver skriftlig tilladelse, vil der blive gemt et reserve blodprøve glas (5 ml) fra såvel baseline som ved 3 måneders kontrol i en af Datatilsynet godkendt biobank (jr.nr 2005-41-5655, senere 2008-41-2031, og fra dec 2009: 30122009.HEH.O.JF). Disse prøver opbevares i kodet form (dvs uden CPR nummer) og på ubestemt tid.

Brug af blodprøverne til andet formål, end hvad forsøgspersonerne oprindeligt har givet tilladelse til, og som er omtalt i nærværende protokol, vil følge de gængse retningslinier, herunder fornyet anmeldelse til videnskabsetisk komité.

Der tages ca. 40 ml blod per gang (2 x i alt). Dette er inklusiv reserve blodprøve glas.

Der ydes ingen økonomisk godtgørelse til forsøgspersonerne.

15. Risiko og rettigheder for deltagerne

Patienterne er ikke frataget for anden kendt effektiv behandling, der tilbydes tilsvarende patienter på hospitaler i København, og deltagelsen i undersøgelsen interfererer ikke med patientens nuværende behandling.
Blodprøver sker via vene punktering og vil blive udført med standardudstyr, hvilket fører til kendt og begrænset ubehag.

DEXA skanning benytter svage røntgenstråler. Stråledosis er ca. 0,1 mSv pr. undersøgelse, svarende til ca. 3 % af naturlig baggrundsstråling pr. år.
Ved måling af tryk-tærskel kan der opstå nogen ømhed eller komme blå mærker, såfremt deltageren ikke følger instruksen og siger stop eller stopper i tide.
HRV stillingstest er non-invasiv og uden ubehag eller risiko for personen.
Den givne aktive intervention er risikofri. Såvel kontrol som aktiv gruppe får en bog om generel stress håndtering.
Forsøgspersonerne informeres om, at de har mulighed for at klage til Patientklagenævnet og desuden søge erstatning via Patientforsikringen.

16. Interessekonflikter

Søren Ballegaard er aktionær i Ull Care A/S og er opfinderen af Ull Meter. For at minimere mulig bias, deltager han ikke i a) patient udvælgelse, b) patient-instruktion og kontakt (han vil alene udøve tilsyn med behandling udført af andre) c) evaluering af behandlingen
Ansvarlig forsker er professor dr.med. Jens Faber, Medicinsk Afdeling O, Herlev Hospital, og han er associeret til Ull Care A/S som uvildig, ulønnet og ekstern, videnskabelig rådgiver. Jens Faber ejer ikke aktier eller lignende økonomiske andele i Ull Care A/S, og har aldrig modtaget økonomisk støtte/honorar fra selskabet.

Ingen øvrige deltagere er associeret til Ull Care A/S, og ingen har økonomisk gevinst af undersøgelsen.

17. Publikationer

Både positive og negative resultater vil blive sammenskrevet til en eller flere videnskabelige artikler og sendt til internationale peer-reviewed tidsskrifter mhp. Publikation. Styre- og ledergruppe vil være medforfattere på alle videnskabelige artikler. Tilknyttede forskere vil blive medforfattere på de artikler, hvor de har bidraget til.

18. Brug af data og anmeldelse til Datatilsynet

Alle rettigheder til data mht. videnskabelig evaluering og publikation ejes af den projektansvarlige forsker, og projektet anmeldes til Datatilsynet efter de for Herlev hospitals godkendte retningslinjer.
Rå data vil i anonymiseret form og efter aftale med projektansvarlige være tilgængelig for alle de tilknyttede institutioner til brug i forskningsprojekter. Alle publikationer skal bekræftes af den hovedansvarlige forsker Jens Faber.

19. Økonomi

Undersøgelsen søges finansieret af såvel private som offentlige fonde. Det samlede budget er på 3.2 mill DKK.

20. Organisation og ansvar

Jens Faber er den projektansvarlige forsker.
Styregruppen består af Per Bech, Åke Hjalmarson og Søren Ballegaard. Søren Ballegaard indgår i styregruppen på grund af hans specielle kompetence hvad angår den aktive intervention, der indgår i undersøgelsen. Jens Faber er adjungeret medlem af styregruppen og således uden stemmeret.

21. Reference oversigtReferences Cited

Alonso, J. et al., 2004, Health-related quality of life associated with chronic conditions in eight countries: results from the International Quality of Life Assessment (IQOLA) Project: Qual.Life Res., v. 13, no. 2, p. 283-298.

Arendt-Nielsen L, and Yarnitskyy D, 2010, Experimental and Clinical Applications of Quantitative Sensory Testing Applied to Skin, Muscles and Viscera: J of Pain.

Axelsson A, Schousen P, Karpatschof B, Nyboe, and Ballegaard S, 2010, Acupuncture and Breast Cancer: A pilot study. 2010: submitted for publication.

Ballegaard, S., E. Borg, B. Karpatschof, J. Nyboe, and A. Johannessen, 2004, Long-term effects of integrated rehabilitation in patients with advanced angina pectoris: a nonrandomized comparative study: J Altern.Complement Med, v. 10, no. 5, p. 777-783.

Ballegaard, S., A. Johannessen, B. Karpatschof, and J. Nyboe, 1999, Addition of acupuncture and self-care education in the treatment of patients with severe angina pectoris may be cost beneficial: an open, prospective study: J Altern.Complement Med, v. 5, no. 5, p. 405-413.

Ballegaard, S., B. Karpatschof, W. Trojaborg, A. M. Hansen, G. Magnusson, and P. B. Petersen, 2009, A simple and objective marker for stress: Scand J Clin.Lab Invest, v. 69, no. 6, p. 713-721.

Ballegaard, S., S. Norrelund, and D. F. Smith, 1996, Cost-benefit of combined use of acupuncture, Shiatsu and lifestyle adjustment for treatment of patients with severe angina pectoris: Acupunct.Electrother.Res., v. 21, no. 3-4, p. 187-197.

Ballegaard, S., P. B. Petersen, F. Gyntelberg, and J. Faber, 2011, Validation of a new stress marker in Danish office workers: A feasibility study: submitted for publication.

Bech, P., 2010, Klinisk psykometri: København, Munkgaards forlag.

Bech, P., N. A. Rasmussen, L. R. Olsen, V. Noerholm, and W. Abildgaard, 2001, The sensitivity and specificity of the Major Depression Inventory, using the Present State Examination as the index of diagnostic validity: J Affect.Disord., v. 66, no. 2-3, p. 159-164.

Bibbins-Domingo, K., G. M. Chertow, P. G. Coxson, A. Moran, J. M. Lightwood, M. J. Pletcher, and L. Goldman, 2010, Projected effect of dietary salt reductions on future cardiovascular disease: N.Engl.J Med, v. 362, no. 7, p. 590-599.

Birket-Smith, M., B. H. Hansen, J. A. Hanash, J. F. Hansen, and A. Rasmussen, 2009, Mental disorders and general well-being in cardiology outpatients--6-year survival: J.Psychosom.Res., v. 67, no. 1, p. 5-10.

Bjorner, J. B., K. Thunedborg, T. S. Kristensen, J. Modvig, and P. Bech, 1998, The Danish SF-36 Health Survey: translation and preliminary validity studies: J Clin.Epidemiol., v. 51, no. 11, p. 991-999.

Bronnum-Hansen, H., M. Davidsen, and P. Thorvaldsen, 2001, Long-term survival and causes of death after stroke: Stroke, v. 32, no. 9, p. 2131-2136.

Carter, C. S., H. Pournajafi-Nazarloo, K. M. Kramer, T. E. Ziegler, R. White-Traut, D. Bello, and D. Schwertz, 2007, Oxytocin: behavioral associations and potential as a salivary biomarker: Ann N Y Acad Sci, v. 1098, p. 312-322.

Dansk Cardiologisk Selskab, 2010, Hjerterehabilitering, in Dansk Cardiologisk Selskab ed., National Behandlingsvejledning 2010.

De, B. G. et al., 2003, European guidelines on cardiovascular disease and prevention in clinical practice: Atherosclerosis, v. 171, no. 1, p. 145-155.

Del Pozo, J. M., R. N. Gevirtz, B. Scher, and E. Guarneri, 2004, Biofeedback treatment increases heart rate variability in patients with known coronary artery disease: Am.Heart J, v. 147, no. 3, p. E11.

Dempster, M., and M. Donnelly, 2000, Measuring the health related quality of life of people

Dimsdale, J. E., 2008, Psychological stress and cardiovascular disease: J Am.Coll.Cardiol., v. 51, no. 13, p. 1237-1246.

Ekman, R., and G. Lindstedt, 2002, Molekyler på liv og død, in R Ekman and B Arnetz eds., Stress; Molekylerne, Individen, Organisationen, Samhället: Sverige, Liber Press, p. 69-89.

Elhendy, A., K. M. Modesto, D. W. Mahoney, B. K. Khandheria, J. B. Seward, and P. A. Pellikka, 2003, Prediction of mortality in patients with left ventricular hypertrophy by clinical, exercise stress, and echocardiographic data: J Am.Coll.Cardiol., v. 41, no. 1, p. 129-135.

Farmer, J. A., 2004, Hypertension and the metabolic syndrome: Curr.Cardiol.Rep., v. 6, no. 6, p. 427-433.

Folker, H., and A. P. Folker, 2008, [WHO-5 as a simple method for measuring quality of life in daily psychiatric clinics]: Ugeskr.Laeger, v. 170, no. 10, p. 830-834.

Grewen, K. M., S. S. Girdler, J. Amico, and K. C. Light, 2005, Effects of partner support on resting oxytocin, cortisol, norepinephrine, and blood pressure before and after warm partner contact: Psychosom.Med., v. 67, no. 4, p. 531-538.

Grippo, A. J., and A. K. Johnson, 2009, Stress, depression and cardiovascular dysregulation: a review of neurobiological mechanisms and the integration of research from preclinical disease models: Stress., v. 12, no. 1, p. 1-21.

Gulliksson, M., G. Burell, B. Vessby, L. Lundin, H. Toss, and K. Svardsudd, 2011, Randomized controlled trial of cognitive behavioral therapy vs standard treatment to prevent recurrent cardiovascular events in patients with coronary heart disease: Secondary Prevention in Uppsala Primary Health Care project (SUPRIM): Arch.Intern.Med, v. 171, no. 2, p. 134-140.

Harkonmaki, K., O. Rahkonen, P. Martikainen, K. Silventoinen, and E. Lahelma, 2006, Associations of SF-36 mental health functioning and work and family related factors with intentions to retire early among employees: Occup Environ.Med, v. 63, no. 8, p. 558-563.

Holmes, S. D., D. S. Krantz, H. Rogers, J. Gottdiener, and R. J. Contrada, 2006, Mental stress and coronary artery disease: a multidisciplinary guide: Prog.Cardiovasc.Dis., v. 49, no. 2, p. 106-122.

Jain, D., T. Joska, F. A. Lee, M. Burg, R. Lampert, and B. L. Zaret, 2001, Day-to-day reproducibility of mental stress-induced abnormal left ventricular function response in patients with coronary artery disease and its relationship to autonomic activation: J Nucl.Cardiol., v. 8, no. 3, p. 347-355.

Koschke, M., M. K. Boettger, S. Schulz, S. Berger, J. Terhaar, A. Voss, V. K. Yeragani, and K. J. Bar, 2009, Autonomy of autonomic dysfunction in major depression: Psychosom.Med., v. 71, no. 8, p. 852-860.

Kristen, A. V. et al., 2010, Acupuncture improves exercise tolerance of patients with heart failure: a placebo-controlled pilot study: Heart, v. 96, no. 17, p. 1396-1400.

Kumazawa, T., 1998a, Primitivism and plasticity of pain--implication of polymodal receptors: Neurosci.Res., v. 32, no. 1, p. 9-31.

Lanza, G. A., K. Fox, and F. Crea, 2006, Heart rate: a risk factor for cardiac diseases and outcomes? Pathophysiology of cardiac diseases and the potential role of heart rate slowing: Adv.Cardiol., v. 43, p. 1-16.

Lewington, S., G. Whithlock, R. Clarke, P. Sherliker, J. Emberson, J. Halsey, N. Qizilbash, R. Peto, and R. Collins, 2007, Blood cholesterol and vascular mortality by age, sex and bloodpressure: a meta-analysis of individual data from 61 prospective studies with 55000 vascular deaths: Lancet, v. 370, p. 1829-1839.

Lewington, S., R. Clarke, N. Qizilbash, R. Peto, and R. Collins, 2002, Age-specific relevance of usual blood pressure to vascular mortality: a meta-analysis of individual data for one million adults in 61 prospective studies: Lancet, v. 360, no. 9349, p. 1903-1913.

Light, K. C., K. M. Grewen, and J. A. Amico, 2005, More frequent partner hugs and higher oxytocin levels are linked to lower blood pressure and heart rate in premenopausal women: Biol.Psychol., v. 69, no. 1, p. 5-21.

Magnusson, G., S. Ballegaard, B. Karpatschof, and J. Nyboe, 2010, Long-term effects of integrated rehabilitation in patients with stroke: a nonrandomized comparative feasibility study: J Altern.Complement Med, v. 16, no. 4, p. 369-374.

Meagher, M. W., A. R. Ferguson, E. D. Crown, S. McLemore, T. E. King, A. N. Sieve, and J. W. Grau, 2001, Shock-induced hyperalgesia: IV. Generality: J Exp.Psychol Anim Behav.Process, v. 27, no. 3, p. 219-238.

Nilius, B., J. Vriens, J. Prenen, G. Droogmans, and T. Voets, 2004, TRPV4 calcium entry channel: a paradigm for gating diversity: Am.J Physiol Cell Physiol, v. 286, no. 2, p. C195-C205.

Nishiyama, S. et al., 2005, [Health-related quality of life in Japanese patients with ischemic heart disease: a multicenter cooperative investigation assessed using SF-36]: J.Cardiol., v. 46, no. 6, p. 211-220.

Noble, R. E., 2002, Diagnosis of stress: Metabolism, v. 51, no. 6 Suppl 1, p. 37-39.

Nolan, R. P., P. Jong, S. M. Barry-Bianchi, T. H. Tanaka, and J. S. Floras, 2008, Effects of drug, biobehavioral and exercise therapies on heart rate variability in coronary artery disease: a systematic review: Eur.J Cardiovasc.Prev.Rehabil., v. 15, no. 4, p. 386-396.

Oldridge, N., A. Perkins, and Z. Hodes, 2002, Comparison of three heart disease specific health-related quality of life instruments: Monaldi Arch.Chest Dis., v. 58, no. 1, p. 10-18.

Olsen, L. R., D. V. Jensen, V. Noerholm, K. Martiny, and P. Bech, 2003, The internal and external validity of the Major Depression Inventory in measuring severity of depressive states: Psychol.Med, v. 33, no. 2, p. 351-356.

Opie, L. H., 2001, Normal and abnormal cardiac function, in E Braunwald, Zippes D.P., and P Lippy eds., Heart Disease: W. B. Saunders, p. 468-469.

Orth-Gomer, K., N. Schneiderman, H. X. Wang, C. Walldin, M. Blom, and T. Jernberg, 2009, Stress reduction prolongs life in women with coronary disease: the Stockholm Women's Intervention Trial for Coronary Heart Disease (SWITCHD): Circ.Cardiovasc.Qual.Outcomes., v. 2, no. 1, p. 25-32.

Pilcher, C. W., and J. L. Browne, 1983, Effects of naloxone and Mr 1452 on stress-induced changes in nociception of different stimuli in rats: Life Sci., v. 33 Suppl 1, p. 697-700.

Pocock, S. J., and R. Simon, 1975, Sequential treatment assignment with balancing for prognostic factors in the controlled clinical trial: Biometrics, v. 31, no. 1, p. 103-115.

Pradhan, A. D., and P. M. Ridker, 2002, Do atherosclerosis and type 2 diabetes share a common inflammatory basis?: Eur.Heart J., v. 23, no. 11, p. 831-834.

Radford, M. J., and J. M. Foody, 2001, How do observational studies expand the evidence base for therapy?: JAMA, v. 286, no. 10, p. 1228-1230.

Rogowski, O., I. Shapira, A. Shirom, S. Melamed, S. Toker, and S. Berliner, 2007, Heart rate and microinflammation in men: a relevant atherothrombotic link: Heart, v. 93, no. 8, p. 940-944.

Routledge, F. S., T. S. Campbell, J. A. McFetridge-Durdle, and S. L. Bacon, 2010, Improvements in heart rate variability with exercise therapy: Can.J.Cardiol., v. 26, no. 6, p. 303-312.

Seematter, G., C. Binnert, J. L. Martin, and L. Tappy, 2004, Relationship between stress, inflammation and metabolism: Curr.Opin.Clin.Nutr.Metab Care, v. 7, no. 2, p. 169-173.

Sher, Y., S. Lolak, and J. R. Maldonado, 2010, The impact of depression in heart disease: Curr.Psychiatry Rep., v. 12, no. 3, p. 255-264.

Sneddon, L. U., V. A. Braithwaite, and M. J. Gentle, 2003, Do fishes have nociceptors? Evidence for the evolution of a vertebrate sensory system: Proc.Biol.Sci., v. 270, no. 1520, p. 1115-1121.

Stansfeld, S. A., H. Bosma, H. Hemingway, and M. G. Marmot, 1998, Psychosocial work characteristics and social support as predictors of SF-36 health functioning: the Whitehall II study: Psychosom.Med, v. 60, no. 3, p. 247-255.

Surtees, P. G., N. W. Wainwright, R. N. Luben, N. J. Wareham, S. A. Bingham, and K. T. Khaw, 2008, Depression and ischemic heart disease mortality: evidence from the EPIC-Norfolk United Kingdom prospective cohort study: Am.J Psychiatry, v. 165, no. 4, p. 515-523.

Szczepanska-Sadowska, E., A. Cudnoch-Jedrzejewska, M. Ufnal, and T. Zera, 2010, Brain and cardiovascular diseases: common neurogenic background of cardiovascular, metabolic and inflammatory diseases: J.Physiol Pharmacol., v. 61, no. 5, p. 509-521.

The National Board of Health,D. [Guidance on heart rehabilitation in hospitals]. 2004. Copenhagen, The National Board of Health, Denmark.
Ref Type: Pamphlet

The network group of "heart rehabilitation". [Heart rehabilitation in Danish hospitals]. 2004.
Ref Type: Pamphlet

Timio, M., G. Lippi, S. Venanzi, S. Gentili, G. Quintaliani, C. Verdura, C. Monarca, P. Saronio, and F. Timio, 1997, Blood pressure trend and cardiovascular events in nuns in a secluded order: a 30-year follow-up study: Blood Press, v. 6, no. 2, p. 81-87.

Turner, E. H., A. M. Matthews, E. Linardatos, R. A. Tell, and R. Rosenthal, 2008, Selective publication of antidepressant trials and its influence on apparent efficacy: N.Engl.J.Med., v. 358, no. 3, p. 252-260.

Uvnãs-Moberg, K., 1997a, Oxytocin linked antistress effects - the relaxation and growth response: Acta Psychologica Scandinavica Supplementum, p. 38-42.

Uvnãs-Moberg, K., 1997b, Physiological and endocrine effects of social contact: Ann N Y Acad Sci., v. 807, p. 146-163.

Villella, M., A. Villella, S. Barlera, M. G. Franzosi, and A. P. Maggioni, 1999, Prognostic significance of double product and inadequate double product response to maximal symptom-limited exercise stress testing after myocardial infarction in 6296 patients treated with thrombolytic agents. GISSI-2 Investigators. Grupo Italiano per lo Studio della Sopravvivenza nell-Infarto Miocardico: Am.Heart J, v. 137, no. 3, p. 443-452.
