# Supplementary material for: The Effect of Daily Self-Measurement of Pressure Pain Sensitivity Followed by Acupressure on Depression and Quality of Life versus Treatment as Usual in Ischemic Heart Disease: A Randomized Clinical Trial
Source: PLoS One. 2014 May 21;9(5):e97553. doi: 10.1371/journal.pone.0097553 (PMC4029626; doi:10.1371/journal.pone.0097553)
Supplement: Protocol S2 — Trial Protocol (English). (PDF) [file pone.0097553.s002.pdf]

PROTOCOL The SongHeart study

Stress, ongoing self monitoring and ischemic heart disease rehabilitation, a randomized controlled trial.

Version 10, date 1100527; VEK reg. Nr. 29698

Principal investigator

Jens Faber, Professor, MD, DMSci [1)]

Steering group

Per Bech, Professor, MD, DMSci [6)]

Åke Hjalmarson, Professor,MD, DMSci [5)]

Søren Ballegaard, CSO, MD [4)]

Participants

Finn Gyntelberg, Professor,MD, DMSci [3)]

Lars Juel Andersen, MD[2)]

Åse Marie Hansen, Senior Researcher, DMSci Pharm [3)]

Jesper Kristiansen, Senior Researcher MSc, DMSci [3)]

Kjeld Møller Pedersen, Professor, DMSci [7)]

Lars Arendt-Nielsen, Professor,MD, DMSci [8)]

Caroline Kistorp, Senior Registrar, MD, PhD [1)]

[1] Department of Medicine O, Endocrine

Unit Herlev University Hospital

University of Copenhagen

2730 Herlev, Denmark

E- mail: jens.faber@regionh.dk

Telephone: +45 38 68 90 16, mobile +45 23 23 51 34

[2] Department of Cardiology

P Gentofte University

Hospital Niels Andersens

Vej 65

2820 Gentofte, Denmark

E-mail: lajuna01@geh.regionh.dk

Telephone: + 45 39 77 22 61, mobile: +45 51 50 27 57

[3] The National Research Center for the Working

Environment Lersø Park Alle 105

2100 Copenhagen OE, Denmark

E-mail: AAMH@arbejdsmiljoforskning.dk

E-mail: jkr@arbejdsmiljoforskning.dk

E-mail: eva@post2.tele.dk

[4] Ull CareA/S

Lemchesvej1

2900 Hellerup, Denmark

Telephone: +45 39 40 41 42, mobile: + 45 40 19 26 45

E-mail: sba@ullcare.com

[5] The Cardiovascular
Institute Sahlgrenska
University Hospital University
of Gøteborg
SE 413 45 Gøteborg,
Sweden E-mail:
ake.hjalmarson@gu.se

[6] Psychiatric Research Unit
Psychiatric Center
Nordsjælland Dyrehavevej
48
3400 Hillerød, Denmark
Telephone:+ 45 48 29 32 53 E-
mail: per.bech@regionh.dk

[7] Health economics
University of Southern Denmark
Copenhagen Department
Øster Farimagsgade 5 A, 2.
1399 København , Denmark
Telephone: +45 65 50 38 47
E-mail: kmp@sam.sdu.dk

[8] Department of Health Science and Technology, Center for Sensory-Motor
Interaction Aalborg University
Frederik Bajers Vej 7
9220 Aalborg, Denmark
Telephone: 99 40 88 30 E-
mail: lan@hst.aau.dk

Abbreviations:

HRV: Heart rate variability: a measure for the balance between the sympathetic and parasympathetic nerve system.

MDI: Major Depression Inventory: A questionnaire which measures depression.

PPS: Pressure pain sensitivity of the chest bone. It is a marker for physiological stress.

PRP: Pressure-Rate Product; systolic blood pressure x pulse rate. The measure is an indicator of cardiac work and cardiac oxygen supply.

SF-36: A questionnaire which reports self evaluated mental and physical health as well as quality of life.

QOL: Quality of life.

Summary

Background: Stress and depression has been found to be mutually linked as well as to have an independent prognostic influence on the survival of heart patients. A new biological measure measuring the Pressure Pain Sensitivity (PPS) of the chest bone has been found to be a reliable marker for physiological stress and to be linked to depression and quality of life. An intervention which uses the PPS measure as a biological feedback marker for stress has been found to reduce the level of stress as well as the level of depression and increase life quality.

Objectives: The primary objective of the study is to examine, if a simple individualized self-care based intervention, using the PPS as a biofeedback marker for stress, reduces the level of depression and stress and improves life quality in patients with ischemic heart disease.

Design: A prospective, randomized single-blinded trial in which the effect of self-care based intervention program in addition to that of best practice in cardiac rehabilitation (REHAB + ULL) is compared to a control group who only receives the best practice in cardiac rehabilitation (REHAB + CARE) over a 3 month period. The study will be open to the patient and the treating professionals but blinded towards the evaluating researchers.

Patients: 300 patients with ischemic heart disease, who have been through a cardiac rehabilitation program at Gentofte University Hospital and Herlev University Hospital respectively.

Effect variables: The primary effect variable is the degree of depression. The secondary effect variables are the level of stress measured by PPS and quality of life.

Treatment: All patients have been through best practice Cardiac Rehabilitation. Half of them, allocated by randomization, receive the self-care based intervention program in addition, which focuses on daily PPS measurement for cognitive reflection and nerve stimulation for stress reduction.

Ethics: All patients continue with their standard cardiac rehabilitation programme as before. The self-care based intervention programme is documented as a risk-free intervention.

Project organization and flow: Jens Faber is the principal investigator. The medical evaluation of the patients will be conducted by an unbiased and blinded nurse in collaboration with a doctor. Total project period is one year.

Perspective: If the present study meets the success criteria, it is concluded that the

intervention is possible and an evidence based choice for reduction of the degree of depression and the level of stress in heart patients.

1. Scientific background

1.1 Prognostic factors in heart disease with special reference to the present trial

There is international consensus that the following factors has a prognostic influence with respect to survival of patients with ischemic heart disease: serum cholesterol (Lewington et al., 2007); blood pressure (Bibbins-Domingo et al., 2010;Lewington et al., 2002); the work of the heart measured as Systolic blood pressure x pulse rate (PRP)(Elhendy et al., 2003), depression (Surtees et al 2008, Sher 2010) as well as chronic stress (De et al., 2003;Dimsdale, 2008;Holmes et al., 2006;Orth-Gomer et al., 2009).

1.2 Definitions and assumptions regarding stress

Even though stress is defined and measured differently in research, there is general agreement regarding the two following aspects:

1) A distinction between transient (acute) and persistent (chronic) stress:

- *Transient stress* is a physiological condition of preparedness, a condition that is automatically induced by neural and hormonal signals from the brain when a threat or challenge occurs. The preparedness functions as a defence mechanism and contributes to an improved performance whether to be used in a flight situation or to solve a work task. When the threat or challenge has been overcome, the preparedness is turned off and the body re-establishes homeostasis.
- *Persistent stress* occurs when one is exposed to the hormones involved in transient stress for a prolonged period. This can lead to psychological and physiological changes that could ultimately be harmful to one's health.

2) The extent of persistent stress depends on the balance between environmental demands and the individual's expectations for the outcome plus the perception of the balance betweendemands and available resources. Additionally, the individual's adaptability also affects the level of stress.

1.3 Stress, depression and ischemic heart disease

In recent years there has been an increased focus on the link between heart disease, depression and chronic stress (Grippo and Johnson, 2009). Evidence of a coincidence of significant metabolic changes for the following conditions has emerged: hypertension, heart failure, obesity, diabetes mellitus, metabolic syndrome, chronic stress, depression and other mental disorders (Koschke et al., 2009;Szczepanska-Sadowska et al., 2010).

The prevalence of depression, as measured as a MDI score ≥ 20, was found to be approximately 20 % in patients with ischemic heart disease (Olesen 2003; Bech 2001).

1.4 Stress measurement

So far there is no international consensus on biochemical diagnostic methods for measurement of transient and persistent stress (Ekman and Lindstedt, 2002;Noble, 2002). This is partly because it is difficult to identify a single physiological measure of stress as stress involves several factors, each of which that has been used as markers of stress in research. These are: 1) objective environmental stimuli, 2) the individual's perception of stimuli, 3) coping mechanism and 4) physiological reactions. These four factors are not necessarily present simultaneously, which has complicated the measurement of stress further (Holmes et al., 2006).

1.4.1 *Biological warning systems and stress (the PPS measure)*

Biological warning systems have always been essential for the survival of living organisms (Kumazawa, 1998a). The ability in animals to discover something in the environment that is a potential threat is developed to a nociceptive system, which involves a reflex making the animal pull back from potential threatening stimuli. The nociceptive system is based on a polymodal receptor, an undifferentiated nerve cell of the same kind as throughout the evolutionary chain from fish to higher vertebras and humans (Sneddon et al., 2003). The receptor is stimulated by mechanic pressure, temperature and acidity.

The sensitivity of the receptor is susceptible to various forms of modulation. One

example is stress-induced analgesia (pain relief) where the pain sensation is suppressed) (Lewis et al 1980). This analgesia helps the wounded animal suppress general pain sensations, thereby letting the animal continue its fight or flight. Another example is stress-induced hyperalgesia (pain hypersensitivity) where the pain sensation is increased. This is reflected in animal studies as an increased sensitivity in paw-pressure tests and tail flick test (Meagher et al., 2001;Pilcher and Browne, 1983).

Clinical observations of heart patients show that an increased tonus in the sympathetic nerve system causes an increased tenderness in certain areas on thorax. A pilot study of 250 healthy people showed a significant connection between the degree of tenderness in certain areas on thorax and the numbers of clinical stress signs. These observations have been confirmed later in a cross-section study (Ballegaard et al., 2011).
Such observations have not previously been described as a part of the warning system in humans, but from an evolutionary biological perspective the survival potential improves by both aspects of stress-induced pain modulation – and through a mechanism that can be detected as far back in the evolution as the fish (Sneddon et al., 2003).

1.4.1.1 *The sensor, the PPS measure and known markers of stress*

Polymodal sensory nerves are responsible for nociception and send their impulses through small myelinized A-delta fibers or unmyelinized C-fibres (Kumazawa, 1998b). They are sensitive to sympathetic input (Nilius et al., 2004). The polymodal sensory nerves in the chest bone have proved to increase their sensitivity during stress (Ballegaard et al., 2009). This can be measured by a new instrument that registers the sensitivity to pain caused by pressure (Pressure Pain Sensitivity, PPS). The PPS measure is simple and has proved reproducible both when measured by the individual and the professional (Ballegaard et al., 2009), and repeated measures do not seem to cause systematic changes conditional to the habituation of the measurement technique (Ballegaard et al., 2011). This means that the PPS measure can become a new tool for stress management: Daily measurements followed by reflection and action as when diabetes patients measure their blood sugar. An intervention program for personal PPS based biofeedback guided stress

management has been developed (see points 1.5 and 9.2.2).

The new medical measure has been shown to be linked to known markers of transient stress such as pulse, blood pressure, salivary cortical and work of the heart measured as Pressure- rate-Product (PRP) (Ballegaard et al., 2009). PRP has been shown to be linked to stress in ischemic heart disease patients with ischemic heart disease (Jain et al., 2001).

In 292 office workers the PPS measure has proven to be linked to both the number of clinical stress signs and the prevalence of depression, as measured by the IC 10 Major depression Inventory (MDI) regarding markers of persistent stress (Ballegaard et al., 2011). Depression has been shown to have a prognostic influence on ischemic heart disease patients (Surtees et al., 2008). PPS was also linked to general mental and physical health measured by the SF-36 questionnaire (Ballegaard et al., 2011) – a questionnaire that reflects the quality of life in patients with ischemic heart disease (Dempster and Donnelly, 2000; Oldridge et al., 2002).

1.4.1.2 *Validations studies of the PPS measurement itself*

In collaboration with researchers from The National Research Center for the Working Environment, Columbia University in New York and University of Copenhagen, a number of validation studies of the PPS measurement have been made in order to enlighten the following:

1. The reproducibility; the ability to reproduce the same measurement values in repeated measurements, was examined in two distinct situations with respectively 5 seconds or a full day between the two measurements.
2. Categorization ability and its reproducibility; the ability to identify groups of people with a higher health risk than another group and the ability to reproduce this categorization.
3. The PPS measure’s specificity and sensitivity when other health markers are used as categorization variables.
4. Risk assessment regarding to the systematic changes in the PPS measure caused by habituation to the measurement technique.

Ad 1: Reproducibility

a) Studies with 5 seconds between the two PPS measurements
    a. In 181 patients in an outpatient clinic the measurements were conducted by a professional and showed high consistency between the first and the second measurement (correlation coefficient r = 0.97, p < 0.001) with a mean difference of 0.3 units (p > 0.5), and a standard deviation (SD) of 5.7 units.
    b. A high consistency between the first and second measurement was also found when ten different professionals measured 308 office workers (r = 0.90, p < 0.001; mean difference 1.32 units, SD = 7.40 units) (Ballegaard et al., 2011).
    c. In a study of 35 opera singers who measured themselves two times daily for a two week period, a total of 698 sets of PPS measurements (r = 0,95, p < 0.001; mean difference = - 0,32 units, SD = 7,6 units).

b) A Bland-Altmann analysis on the studies mentioned above (a, b and c) shows an uniform measurement difference between the first and second measurement on the entire scale (Ballegaard et al., 2011).

c) Studies with a full day between the two PPS measurements:
    a. There was a significant correlation between a total of 318 sets of daily measurements (morning and same-day evening) in the study with 35 opera singers who measured themselves twice a day for two weeks; correlation coefficient r= 0.91 (p < 0.0001); mean difference: 2.2 units (SD = 10,5 units)(p < 0.001).
    b. In another study where 60 office workers measured themselves at home for three months, there was also a significant correlation between a total of 1854 sets of daily measurements: correlation coefficient r = 0.87 (P < 0.0001), mean difference + 2.2 units (SD = 8.9 units) (p < 0.001).

Ad 2: Categorization

a) The PPS measurement’s ability to make a 2-parted classification of people in ”persistent stressed” (PPS ≥ 60) and “non-persistent stressed” (PPS < 60) was examined through a ROC analysis of the 292 office workers (Ballegaard et al., 2011) compared to an analog 2-parted classification ”having an elevated health risk marker” and ”not having an elevated health risk marker”

for the following effect variables and based on the following discrimination criteria: 1) the number of clinical stress signs (less than 10/ 10 or more)(average PPS 49/58; $p < 0.001$), 2) SF-36 (above or below the 25 % percentile compared to the normal Danish population matched for gender and age): overall physical health (PCS scale)(average PPS 49/56; $p = 0.004$) and mental health (MCS scale) (average PPS 49/57; $p = 0.004$),and 3) the prevalence of depression measured by the Major Depression Inventory (MDI) questionnaire (MDI score below 20/equal to or higher than 20) (average PPS 51/61; $p = 0.012$). For all of the above variables there were significantly higher PPS values for people who were initially categorized as being in the group with higher stress level.

b) The PPS measurement's ability to reproduce classification of people by repeated measurements is illuminated in two aspects with respectively five seconds and a full day between two measurements:
    a. Studies with 5 seconds between two measurements:
        i. 10 different instructors' measurements on 292 office workers; probability of uniform classification (PPS ≥ 60): 89 %
        ii. 698 self-measurements in 35 opera singers; probability of equivalent uniform classification: 82 %
        iii. Two different instructors' measurements on 181 outpatients. Here the equivalent probability was 83 %.
        iv. If a tolerance of 1 standard deviation in the second measurement is introduced, the same probabilities would be 97 %, 95 % and 98 % respectively (Ballegaard et al., 2011).
    b. Studies with a full day between two measurements:
        i. The probability of a uniform classification (PPS ≥ 60) is 87 % when all 6004 morning PPS measurements on Ull Care's web platform per 12th of April 2011 are compared to the same-day evening measurements.
        ii. The probability of an equivalent uniform classification among the 318 day measurements made by 35 opera singers

during two weeks was 83 %.

Ad 3: The PPS measurement's specificity and sensitivity
When the discrimination criteria mentioned above are used for a two-parted categorization regarding grouping from a PPS measurement that is 60 units, the specificity was 0.79 and the sensitivity 0.32 was found in terms of the prevalence of clinical stress signs, mental and physical health measured by the SF-36 and the prevalence of depression measured by the MDI. The result is deemed acceptable since the high specificity ensures that a large percentage of the people not in need of treatment are not offered. The low sensitivity can cause some people to be offered treatment even though the need is not present, but since the treatment is harmless and without side-effects, it is deemed acceptable.

Ad 4: Risk assessment in regard to habituation bias of repeated PPS measurements The studies hitherto implemented cannot definitely exclude that measure adaption affects the measurement result when PPS is measured daily over a period of time, but the following studies suggest that such influence does not have decisive significance in patients' everyday clinical practice.

a) A review of the scientific database PUBMED was unable to provide scientific evidence that the phenomenon exists, which in itself obviously does not mean that the phenomenon does not exist.
b) In two case-control database studies in patients with ischemic heart disease and stroke respectively and at 3 to 4 years of observation, the individual patient used the daily PPS measurement as a biological biofeedback marker of stress in regard to cognitive reflection on his/hers stress level. The results showed a significant decrease in the use of health services and an improved survival rate in both studies (Ballegaard et al., 2004;Magnusson et al., 2010).
c) In two randomized intervention studies with respectively 50 opera singers and 308 office workers and with respectively 6 and 3 months of observation, a decrease in PPS was linked to significant and clinical relevant improvements in known risk factors such as blood pressure, pulse rate, work of the heart and serum cholesterol (Ballegaard et al., 2011).
d) A statistical analysis of 6004 PPS self-measurements on the Ull Care web

platform, where at least one measurement of the day is >30, shows that there is a significant increase in the group as a whole from morning to the same day evening (mean) = 2,9 units; SD = 8,4; p < 0,001), and an equivalent decrease in the PPS measure from bedtime to the next morning (mean = - 3,1 units; SD = 8,6, p < 0,001) (Ballegaard et al., 2011).

e) It has been found that mean PPS differences between morning and the same evening during four calendar months for 60 office workers were; +2.0, +2.0 , + 1.0, and +3.1
(232, 785, 624 and 213 person days, respectively). When evening PPS measures were compared to the PPS measure for the next morning, the corresponding numbers were:
- 2.0, - 1.0,- 3.0, and – 2.4, respectively (both p for trend > 0.1).

f) A possible explanation of the continued usefulness of the PPS measure may be due to the fact that it is rarely the same polymodal sensory cell that is the most tender two days in a row, and therefore the measuring point is moved to a new sensory cell. This makes the risk of pain threshold adaption due to repetitive sensory stimulation limited. The individual polymodal sensory cell has a measure area of about 1 square millimeter (Kumazawa T 1998), the measure foot pad an area of about 1 square centimeter and the measure area itself is the size of a palm (about 50 square centimeters).

Summary of the validation studies

- There is a satisfactory agreement between repeated measurements both when taken with a 5 second interval and when measuring in the morning and the same day evening.
- A classification of people with PPS ≥ 60 as belonging to a group a people with an elevated health risk profile compared to people with a lower PPS measure seems clinically useful and reproducible.
- A possible clinical and decisive importance of systematic errors caused by habituation to the measuring technique does not seem to be the case, neither for the ability to obtain relevant clinical effects after respectively months or years of PPS measuring, nor as to the possibility of using changes in the PPS measure as a marker of stress in the daily cognitive reflection.

1.4.1.2 Compliance of domestic PPS measuring

Compliance was 90 % in the study with 35 opera singers, who measured themselves twice a day for two weeks, whereas it was 20 % among the 60 office workers, who measured themselves daily for three months. Given the results achieved among the 60 stressed office workers, it does not seem a concern that their measure compliance was only 20 % measured over a three month period The reasons for the lower compliance can be many: 1) that not all measurements are entered, 2) that measurements are not entered when they have been low for a long time (e.g. < 40), 3) that in the long term it is not necessary to measure daily, but maybe just a few times per week or whenever the need for cognitive reflection is present.

1.4.2 *Questionnaires (MDI, SF-36, WHO-5)*

Major Depression Inventory (MDI) is a valid and reproducible questionnaire for measuring depression (Bech, 2010).

SF-36 is a questionnaire that measures quality of life and has shown to be a valuable prognostic tool in patients with ischemic heart disease (Dempster and Donnelly, 2000;Nishiyama et al., 2005;Oldridge et al., 2002). In addition, the SF-36 has been used in large population studies to assess the long term health damaging effects of work related stress (Stansfeld et al., 1998). A connection between SF-36 and early retirement from the labor market has also been found (Harkonmaki et al., 2006).

WHO-5: Measures well-being. A study has shown that those heart patient who have a low score (< 50) on the WHO-5 questionnaire has a significant higher mortality within six year than patients with a high score (> 50) (Birket-Smith et al., 2009).

1.4.3 *Metabolic variables and stress*

Persistent stress has been associated to the development of Metabolic Syndrome (MES) (Folkow B 2001) (Rosmond R 2004). MES is a cluster of risk factors for the development of cardiovascular diseases including 1) reduced glucose tolerance/diabetes type II, 2) abdominal fat deposition, 3) dyslipidemia (disturbed lipid metabolism, and 4) hypertension (high blood pressure). These risk factors are

considered a significant health problem in the western world and affect 40 million Americans (Farmer, 2004;Ford, 2005).
Insulin resistance is regarded as one of the key factors in the development of MES. Moreover, a review by Sutherland et al. 2004 points that MES is associated or caused by chronic low-grade inflammation due to activation of the immune system. Chronic low-grade inflammation is probably also a relevant factor in the development of atherosclerosis disease in the heart (Pradhan and Ridker, 2002). Insulin resistance, MES and chronic low-grade inflammation are all associated with endothelial dysfunction, which is characterized by blood vessels becoming stiffer and changes in the blood vessels' structure. Seematter et al. (2004), finds that these changes are connected with the development of atherosclerosis.

1.4.4 *Blood pressure as a marker of stress*

Obesity, insulin resistance, hypertension and dyslipidemia (all seen in MES), are each a risk factor for cardiovascular disease and early death (Farmer, 2004).
The increase in blood pressure often seen with increasing age has shown to be related to persistent stress (Timio et al., 1997).

1.4.5 *Heart-rate variability and stress*

Decreased heart-rate variability (HRV) has proven to be associated with increased stress and bad prognosis for heart disease. In studies with a stress reducing intervention, HRV has improved (Del Pozo et al., 2004;Nolan et al., 2008;Routledge et al., 2010).

1.4.6 *Pulse and Pressure-rate Product as stress markers*

Resting pulse has proven to have a prognostic value in ischemic heart disease (Lanza et al., 2006), and the link has proven to be the degree of persistent stress (Rogowski et al., 2007). Pressure-rate Product (PRP) is the product of the systolic blood pressure and the pulse, which is used in cardiology as an index of heart's ability to obtain oxygen and therefore reflects the work of the heart. The work of the heart is mainly controlled by beta-adrenergic catecholamine receptors (Opie, 2001), which makes the PRP suitable as an indicator of the sympathetic tonus in the heart (Noble, 2002) and suitable as a prognostic marker of ischemic heart disease (Villella et al., 1999).

A previous study has shown a significant link between resting pulse, PRP and the PPS measure (Ballegaard et al., 2009), yet we have opt to use blood pressure, pulse, Pressure-rate product and serum cholesterol as primary and secondary variables in the Songheart study because the study's primary target group are receiving medication that affects these variables.

1.5 The PPS measure, stress and Diffuse Noxious Inhibitory Control system (DNIC)

Changes in DNIC might in part explain the increased pain sensation among chronically stressed people (over reaction of the ascending stimulating nerve signal in combination with a reduced descending and inhibitory nerve signal (Arndt-Nielsen et al. 2010). This fits with a plausible hypothesis for the effect of daily nerve stimulation (acupressure), where the persons own stimulation followed by control for reducing tenderness seems to result in a restitution of the lost DNIC function, typically for the chronically stressed person.

1.6 The effect of the intervention that uses PPS as a biofeedback marker of stress

The use of PPS as a biofeedback marker in combination with a multifaceted stress handling program has been validated in two bloc-randomized controlled experiments; one smaller with 50 opera singing students (Ballegaard et al., 2011) and a larger with 292 office workers (Ballegaard et al., 2011).
In the first study 50 opera singing students from two different schools were randomized either to an active treatment group or a control group. The active treatment is comparable to the treatment that is used in this study, while the control group had a two hour lecture on general stress handling. Compared to the control group the active group obtained a decrease in pulse (14 %), a decrease in work of the heart measured as PRP (14 %), a decrease in the number of clinical stress signs (67 %), in PPS (35 %) and in the total number of elevated health risk markers (60 %) (all $p < 0.05$) (Ballegaard et al., 2011).

In the subsequent larger study on 292 office workers, the participants were divided

into two groups based on a PPS measurement; very stressed (PPS ≥ 60) and little to moderate stressed (PPS <60). The very stressed were bloc-randomized and placed in either an active treatment group where they receive a PPS measuring instrument plus a personal guide to the programme in a three month period, or a control group. The results showed that when the active group was compared to the control group, the intervention had had a clinically useful and scientific significant effect in blood pressure (10 %), work of the heart (10 %), serum cholesterol (13 %) and PPS (42 %) (all p < 0,001) and with an 80 % response rate (Ballegaard et al., 2011).

In a prospective uncontrolled study on a random selected group of women with breast cancer, the effect of the intervention gave the following results on PPS (Axelsson et al., 2011). The average change was from 78 to 56 in PPS measure, compared to an average change from 74 to 49 among the 60 office workers in the active group of the previously discussed study (Ballegaard et al., 2011). These women also experienced a significant improvement in level of anxiety and depression, and the improvement in PPS was linked to the improvement in level of depression. Furthermore the feasibility was high, more than 90 % of the women were still using the program six months after start (Axelsson et al., 2011).

A prospective, uncontrolled, clinical case-control database study of 10 years with 160 patients with ischemic heart disease (all were candidates for invasive treatment), who used finger palpated tenderness on the sternum as a biofeedback measure of PPS in combination with a comprehensive self-care based stress management program, showed that the intervention had a long lasting beneficial effect on mortality and healthcare expenses (Ballegaard et al., 2004). The three year risk of dying was significant lower than the general Danish population's and there was a considerable decrease in use of medication, number of hospitalization days, and a decreasing demand of invasive cardiologic treatment, which overall led to an approximately 70.000 DKK reduction of the yearly health expenses per patient (based on the costs ten years ago). These patients started with three weeks of acupuncture treatment while the self-care part was the most important contribution for the rest of the observation period (Ballegaard et al., 2004; 1999;1996).

With respect to psychosocial characteristics the patients were found to have similar characteristics as heart patients receiving pharmaceutical treatment at a hospital outpatient clinic, both being significantly different from a group of healthy controlled and a group of patients without heart disease (both $p < 0.05$) (Ballegaard et al., 1996).

In the same study series, it was found that at baseline there was a significant correlation between the degree of depression and the degree of disease measured by NYHA- classification (correlation coefficient $r = 0.4$, $p < 0.001$), and in addition that the degree of depression at baseline correlated independently to the effect measured as change in the NYHA-classification ($r = 0.6$, $p < 0.001$). The intervention was found to have a significant effect on quality of life, need for medication and degree of disease (all $p < 0.001$) measured by NYHA-classification (Ballegaard et al., 1999).

The finding of a beneficial effect in terms of improved survival when stress management is used by patients with ischemic heart disease has recently been confirmed in randomized studies (Gulliksson et al., 2011;Orth-Gomer et al., 2009). Other researchers have shown that sensory stimulation through acupuncture improves capacity and HRV in patients with heart disease (Kristen et al., 2010). Evaluated in a prospective, uncontrolled, clinical case-control database study the same program in patients with stroke has shown to reduce the cumulative 4-year mortality to a level that corresponds to the general Danish population (Magnusson et al., 2010), against an expected excess mortality of 50%. Half of the deaths after stroke can be attributed to ischemic heart disease (Bronnum-Hansen et al., 2001).

Conclusion on an effect of the selected active intervention:

- The PPS measure has shown to be linked to physiological stress, work of the heart, degree of depression and quality of life.
- Two long-term prospective database studies show a pronounced effect on the consumption of healthcare services and survival without risk for the patient. An editorial article in the prestigious JAMA concludes that when the effect is large and the risk small, prospective observation studies are sufficient as a basis for introducing clinical recommendations (Radford and Foody, 2001).

- Psychological questionnaires show that the patients in these observation studies do not differ from heart patient in medical treatment in terms of psychological conditions.
- There has been observed effect in terms of stress reduction in a group of unselected patients (with breast cancer).
- In two prospective randomized studies in people who are engaged in active employment there has been shown effect on the level of stress on the central heart and health risk markers.
- The treatment has in all studies proved harmless. When the intervention has shown its stress reducing value in not-randomized studies with patients with a life threatening disease such as heart disease, stroke and breast cancer, and subsequently shown in randomized studies with a general population in active employment, the next natural step is to examine whether the effect is strong enough to show as stress reducing in a randomized study in one of the groups of patients with a life threatening disease where the intervention has already proven effective – here ischemic heart disease. If the effect is shown to be powerful enough to confirm the earlier findings, now in a randomized study, then the effect is expectedly also strong enough to reduce the stress strain in patients with other life threatening diseases – and in patients with chronic non life threatening illnesses.

1.7 The effect of the used heart rehabilitation

The used heart rehabilitation programs show no effect on the cardiac life style risk factors and mainly serve to withhold compliance in relation to the medical treatment (Danish Society of Cardiology, 2010).

2. Hypothesis

In the light of the above mentioned, we find it relevant to examine the effect of the active intervention in a prospective randomized design in patients that have been through a heart rehabilitation program, in order to illuminate its eventual value, provided that the intervention was given to Danish heart patients as a supplement

to the existing rehabilitation.

Hypothesis: Can a simple, individualized and self-care based intervention program, that frequently uses the PPS measure as a feedback marker of stress followed by nerve stimulation in order to reduce the level of stress, subsequently expecting increased personal empowerment: improved degree of stress, depression, well-being and life quality (QOL) plus a number of factors affecting exacerbation of the heart disease with potential favorable impact on the consumption of healthcare services and long-term prognosis?

It is internationally recognized that there is a link between stress and depression (Grippo and Johnson, 2009). By involving the depression measurement as a way to measure stress reduction, the new stress measurement (PPS) is linked to a known measurement (depression). In continuation of this, it is also recognized that depression has a primary and secondary negative prognostic significance in heart disease and that reduction of depression symptoms has a beneficial influence on the patients' prognosis in regard to "hard" endpoints, meaning hospitalizations, need of operations, relapse and death. The same applies to well-being measured by WHO-5. It will also be interesting if the patients that embrace the new technology have a clinical effect, even though such an effect cannot be proven for the active group as a whole. Therefore the secondary effect variable is the correlation between change in PPS and change in MDI and WHO-5 scores, in the sense that from a cardiac point of view it will be fully satisfactory, if the study shows that heart patients, who get a better PPS also achieves an improved quality of life if their quality of life is impaired at baseline.

3. Purpose

The study has two main purposes:

1) **To test, based on the data from the baseline study, whether:**
    - There is a connection between the PPS measure and chronic stress measured as quality of life, occurrence of depression, heart rhythm variability and work off the heart in patients with ischemic heart disease.

- There is a connection between PPS and chronic stress estimated as a forerunner of metabolic syndrome. Metabolic syndrome is in this study primary valued based on decreased insulin resistance/disturbed sugar metabolism.
- There is a connection between PPS and chronic stress measured as altered body fat distribution, i.e. altered relationship between android and gynoid fat distribution.

2) **Based on the data from the intervention study:**

- To investigate whether two key elements in the aforementioned self-cared based intervention program (the Ull Care program), PPS measurement as biofeedback marker of stress and nerve stimulation for stress reduction, may help patients with ischemic heart disease through increased self-care to:
- Reduce chronic stress measured over a period of three months:
  - A reduction in depression and improved quality of life
  - A reduction in the stress measure PPS
  - Beneficial effect on selected cardiovascular risk factors, e.g. work of the heart, HRV, lipids
- Over a period of 12 months to also lead to:
  - A reduction in consumption of health services
  - A positive effect on the patient's disease prognosis

3) Several sub studies are planned:

1. Due to the chosen randomization procedure it will be possible to perform analyses on the randomized intervention trial on the subgroups listen in section 8:
   age, gender, diabetes mellitus, heart failure, time from last myocardial infraction or invasive cardiological procedure. Degree of depression is listed among the main purpose, section 2.

2. Comparison of the chosen PPS measure with another and established algometer.

4. Design

Prospective, randomized, controlled, single-blinded study with 50 % allocation ratio for intervention. The time course of the intervention and control group is parallel. The study takes place at Herlev Hospital and is open to the following: participating patients and the instructor of the active group. The study is blinded to all others, including the statisticians who must make the main analysis. The personnel conducting the clinical examinations do not have access to the documents containing information about the treatment or treatment procedures. This is to prevent bias in the comparison between the treatment groups.

5. Material

5.1 Subjects:

The study includes 300 patients with ischemic heart disease. The patients will be recruited through the center for cardiac rehabilitation at Gentofte and Herlev Hospital respectively.

5.2 Inclusion criteria:

- Implemented cardiac rehabilitation program at Gentofte or Herlev Hospital minimum 6 months ago.
- Documented ischemic heart disease that qualified for a heart rehabilitation program.
- Use of aspirin, cholesterol-lowering and antihypertensive medication after Danish cardiac guidelines.
- Younger than 75 years at the time of inclusion.
- Elevated level of stress, defined as PPS $\geq$ 60.

5.3 Exclusion criteria

- Previously diagnosed and treated for a psychiatric disorder, except for depression.
- A chronic competing disorder that statistically is life-shortening (such as advanced cancer with metastases).
- A chronic competing disorder that is neither heart disease nor diabetes and which clearly impairs the patient’s quality of life /e.g. COPD, cancer, chronic pain syndrome).

- Patients who cannot take care of themselves.
- Patients who, for various reasons, find it difficult to participate in the intervention program (e.g. language barriers).
- A scheduled cardiac surgery.
- Previous treatment with the examined active treatment.
- A change in heart medication (in terms of angina pectoris and/or heart failure) within the last month.

NB: Patients may participate in other clinical trials at the same time because this is not an exclusion criterion.

6. Effect variables (the intervention study)

For the baseline study additional blood samples are taken in relation to what is mentioned in section 6.3.1 below (see point 9.3).

6.1 Primary effect variable:

The degree of depression by the MDI questionnaire (Bech et al., 2001;Olsen et al., 2003)

6.2 Secondary effect variables:

1. Physiological measurements of stress: Level of stress measured by PPS by the same blinded nurse (trained in PPS measurement) at baseline and after three months of observation.
2. The WHO-5 questionnaire measuring well-being (QOL) (Folker and Folker, 2008).
3. Correlation between changes in PPS and changes in MDI and WHO-5 scores respectively.

6.3 Tertiary effect variables:

6.3.1 *Biological variables:*

1. Measure of persistent stress: serum Oxytocin, HbA1c.
2. Insulin resistance: Fasting plasma insulin, C-peptide, blood glucose (with calculation of HOMA), HbA1c, Adiponectin.
3. Chronic inflammation: Serum / plasma high sensitive CRP, Defensin, YKL-40.
4. Fat metabolism: total cholesterol, LDL and HDL cholesterol, Triglycerides.

5. Heart strain NT-proBNP and BNP.

6.3.2 Physiological variables:

1. Blood pressure, pulse, blood pressure pulse product (PRP).
2. Body Mass Index, (height and weight).
3. Body fat composition by a DEXA scan.
4. Heart rhythm variability (HRV) measured by tilt testing.

6.3.3 General QOL

1. Measured using the SF-36 questionnaire (Alonso et al., 2004;Bjorner et al., 1998).

6.3.4 Health economics (cost / benefit) analysis

Data from the last 12 months prior to the inclusion date will be used as baseline variables. This is only executed if the primary and secondary effect objectives meet the criteria for success (see paragraph 12). Data will be collected for the following services:

1. Number of visits to the practitioner (for heart problems)
2. Number of visits to a cardiologist (specialist)
3. Number of hospital visits (outpatient)
4. Number of inpatient days in a hospital

5. Use of invasive procedures (PCI and/or CABG)
6. Use of cardiovascular medicine
7. Number of sick days due to heart disease (if the person is working)

6.3.5 *Other variables:*

a. Frequency of clinical event: measured by a composite endpoint when one of the following occurs: 1) visit to a hospital for a cardiac reason; 2) hospitalization of cardiac causes and 3) cardiac death. Common visits to the GP will not be counted.
b. Patient compliance: assessed by 1) number of patients who follow the program until the end of the trial, 2) personal view of the usefulness of the program on a 7 point ordinal scale, and 3) compliance of the Ull Care program as measured by the degree of integration into daily life on a binomial scale.
c. The number of clinical stress signs based on a designed and validated questionnaire (Ballegaard et al., 2011)
d. Changes in medication, to ensure that any observed improvement is not due to an increase in medication prescribed by physicians outside the study protocol.
e. Use of invasive procedures (PCI and CABG).
f. Number of days on leave due to heart disease

7. Practical course (including flowchart)

Using databases of cardiac rehabilitation patients from Gentofte and Herlev Hospital respectively approximately 600 patients with complete cardiac rehabilitation course > 6 months ago, to ensure a steady state in regard to the efficacy of the given cardiac rehabilitation, are identified. The patients are invited by letter to participate in the study.

It is expected that about 450 will accept. These are convened to an examination at Herlev Hospital Medical Department O.

All patients sign the consent form and go through the physiological preliminary examination.

Patients with PPS < 60 are ended immediately, and a subset of the first 50 patients with PPS < 45 are invited to a measuring of their body composition.

Since it is expected that 2/3 of patients have an elevated PPS measure (≥ 60), 300 patients with PPS ≥ 60 are randomized to either an active or control group with 150 in each:

Control group: Continue their own efforts in extension of the already given cardiac rehabilitation and receive a textbook on general stress management (REHAB + CARE).

Active group: the same plus the Ull treatment program (REHAB + ULL).

All included patients will at baseline be offered an additional measurement of body composition.

Intervention period: 3 months.

Both the control and active group continue with the given medical treatment. If there is a change in heart condition including possible side effects of medication, the medication is adjusted according to the procedures in the rehabilitation programs at the hospital the patient is assigned to (Gentofte or Herlev Hospital). If possible, the medication should remain stable during the 3 months of the observation. The patient and the patient's own doctor are informed that the aim of the study is that any adjustment of cardiovascular medicine happen after contact with the project nurse (perhaps through a telephone hot-line).

Control / Active group:

1. Control group: receive a book about general stress management
2. Active group: receive in addition to the above mentioned:

Instruction in self-measurement of PPS by a PPS instructor at home. The patient is instructed in PPS measurement, nerve stimulation and the use of the web platform in order to be able to enter their own measurements. They are not instructed in stress management. There is a 45 minute home visit in the beginning plus two visits after one and two months respectively for additional technical instruction if the PPS measure is ≥ 60. In addition there are 5 telephone dialogues á 15 minutes solely for technical instruction after 1, 3, 5, 6 and 10 weeks respectively. The patients receive an Ull meter, a written user's manual and an instructional DVD.

The patients record their PPS measurements either on the web platform (www.songheart.org) or on paper at least once a week

If their PPS records show a PPS ≥ 60 for 7 consecutive morning measurements, the patient is contacted regarding the need for additional technical instruction.

After three months of participation in the project all patients (both groups) are called to a repetition of the baseline visit at Herlev Hospital. Those who at baseline opted to have made body composition will have the same offer again if, at the physiological examination, they have lost 3 kg or more.

The two examinations at Herlev are conducted by the same blinded nurse at baseline and the 3 month follow-up respectively.

If the success criteria for the study are met, the subsequent analysis are carried out after the 12 month observation:

Use of health services over the last 12 months before the start of the study compared to the 12 months after commencement of participation.

The prevalence of clinical events. Here a Kaplan-Meier plot analysis will be used. The Minimal Important Difference (MID) between the active and control group is set at a 10 % improvement in the active group versus the control group.

7.1 Flowchart:

Visit (number)	1	2	3	4
Physical location	Herlev	Home visit	Herlev	Data collection via public records
Months	0	0	3	12
Window for visits		+/-7 days	+/-7 days	
Informed consent	X			
Questionnaires completed	X		X	
Inclusion/exclusion	X			
Blood pressure, pulse, PPS and HRV test	X		X	
Blood sample	X		X	
Assessment of medication	X		X	
Randomization	X			
Registration of clinical events	X			X
Consumption of healthcare services	X			X
Implementation of joint treatment	X			
Implementation of active treatment		X		
Measurement of body composition (subgroup)	X		X	

CONSORT Flow Chart for the SongHeart study

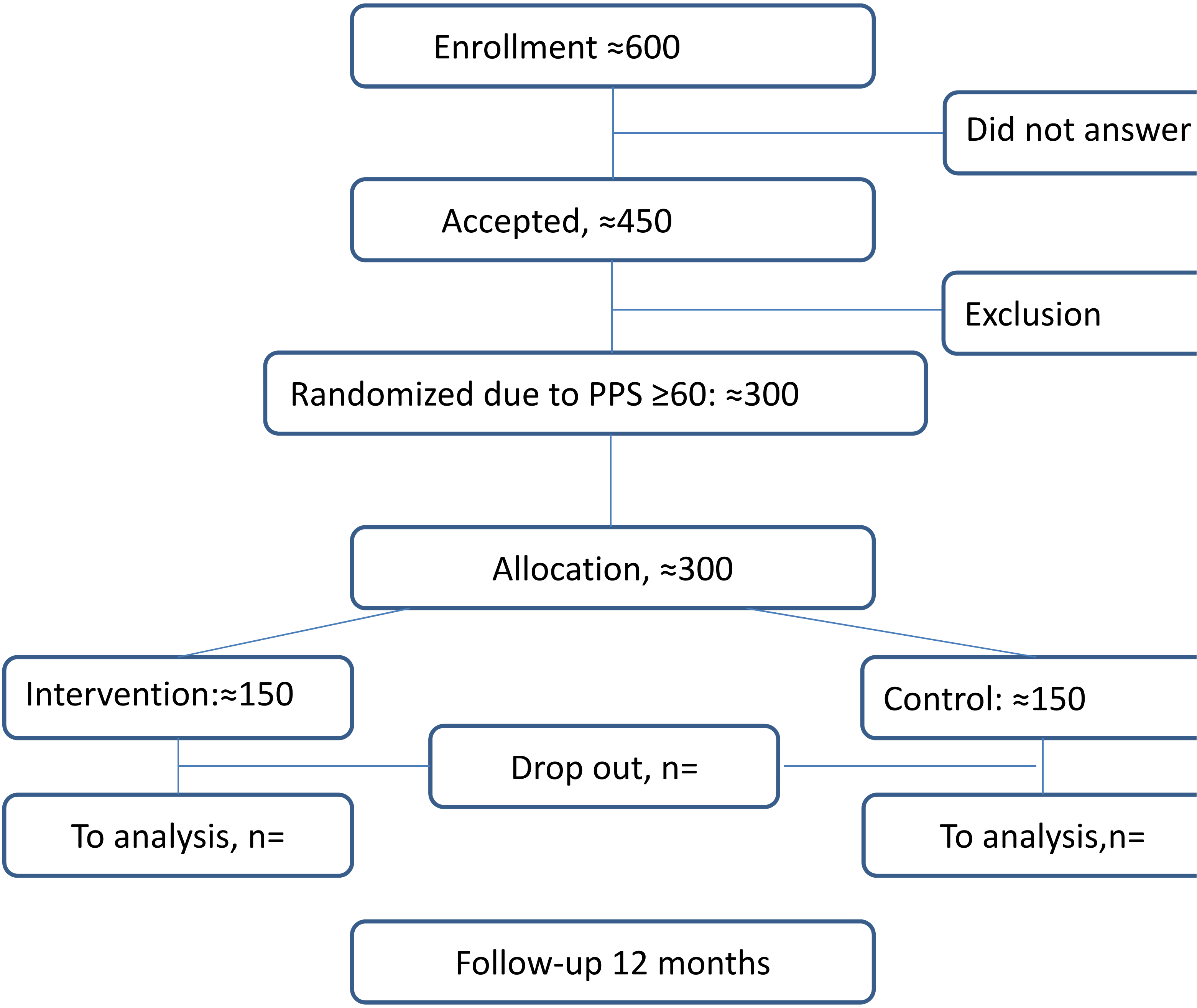

8. Randomization procedure

The treatment is assigned randomized and with an equal number of patients in the control group and for active intervention. An independent research company electronically conducts the practical randomization (www.larix.dk).

The treatment is allocated by a minimization procedure with built-in randomness (Pocock and Simon, 1975). The minimizing will ensure an approximate balance in the number of patients in both treatment groups with respectively low and high values of the baseline parameters taken into account in the procedure. Pocock and Simon's minimization method (Pocock and Simon, 1975) will be used with a randomness parameter of 0.8. The following baseline parameters will be included:

Age (< 65, ≥ 65 years)
Gender (male, Female)
Heart failure (Yes, No)
Diabetes (Yes, No)
MDI (score <15, ≥ 15)

The result of the randomization is blinded to the investigating nurses and doctors and other who are hired to participate in the study, including the statisticians who analyze the data in relation to the main scientific article. The people conducting the training of the active group as well as patients are excluded from blinding.

9. Methods

9.1 Stress measurement:

An Ull Meter is used, which is an instrument developed to measure the sensibility/activity in the polymodal nervous system corresponding to the most painful point on the sternum between costa 3-5. The point is identified by finger pressure. First the patient learns his/hers pain threshold as the instructor applies a gradually increasing pressure on the tibia with the instrument and the patient says stop when the threshold of pain/discomfort is reached. The procedure is then repeated on the most tender place on the sternum while the patient himself

applies the pressure and stops when the pain threshold is reached. The instrument then displays a number on a scale from 30 to 100, where an increased sensibility is accompanied by an increasing number, PPS; meaning that a high PPS measure reflects a high level of stress (high sensitivity) (Ballegaard et al., 2009).

The Ull Meter has the following special features:

- Hidden measurement value until the measurement is completed.
- A calculation of the sensitivity to a tenderness score on a logarithmic scale similar to the decibel scale used to estimate the limit values of sound pressure in connection with a hearing test.
- An alarm that is activated by a pressure before the occurrence of any injury.
- A special foot pad that prevents irritation of the skin to ensure that it is the pressure sensitivity of the bone that is measured and not the skin's sensitivity.

9.2. Treatment modalities:

9.2.1 *Current cardiac rehabilitation:*

All patients have been through and take part in the cardiac rehabilitation as defined by the respective cardiology departments and according to public health regulations (The National Board of Health, 2004;The network group of "heart rehabilitation", 2004)

9.2.2 *The Ull Care program:*

The basic elements of the intervention are as follows:

1. A self-care part.
2. A professional instruction in the PPS measurement, cognitive reflection in relation to the measure and nerve stimulation.
3. Continuous recording of the participants' PPS measurements through a Web journal.

The self-care based intervention program is a stress management program based on the use of the PPS measure as a biofeedback marker of stress. The most important effort is the daily

self-care in the form of PPS measuring, cognitive reflection and nerve stimulation, which is linked to professional technical help depending on need.

The elements of the self-care program

An individual start-up of a 45-minutes face-to-face consultation at home with an Ull Care instructor who explains the use of the instrument, the interpretation of the PPS measurement, nerve stimulation and the web platform.

A personal Ull Meter instrument.

A personal web journal for track recording of the participant's PPS measure's development (patients without internet access will receive printed registration sheets).

A booklet that describes the self-care program in details.

Two additional face-to-face meetings with additional technical instruction should the PPS measure remain ≥ 60 after 1 and 2 months respectively.

5 x 15 minutes of telephone consultations after 1, 3, 5, 6 and 10 weeks respectively. Only the following four subjects may be discussed: the PPS measure, interpretation of the measurements, nerve stimulation and the use of the web journal. This is to ensure a correct use of the Ull Meter instrument and a correct management of the nerve stimulation technique.

Online assessment of the participant's PPS measurements. This assessment is made automatically on the website. If the morning PPS measure has been ≥ 60 for more than seven days in a row, the participant is offered additional technical support.

The daily steps of the self-care program

Four elements constitute the daily self-care system: measuring, cognitive reflection, action and control.

Measuring: PPS is measured twice a day as a fully private measure and is entered in a personal web journal, so that the person can track his/hers own development, while at the same time there is a regular extern professional control.

Reflection: Cognitive reflection as a reaction to the actual PPS measure and its development.

Action: Nerve stimulation on selected acupuncture points corresponding to the

instruction. Possible further own efforts are as mentioned in the book on general stress management given to all participants (both the active and control group). The PPS measure is used as a personal feedback marker of stress, taking into account the effect of the participant's chosen effort.

Control: Measure the effect of any action/inaction at any time.

This four-step procedure should be done at least twice a day (morning and evening) to be preventive; it is recommended to perform the procedure morning and evening in order to promote the construction of a conditioned reflex. This reflex (i) facilitates the habit and (ii) improves the effect of the action, which is important for ad-hoc use, should the participant want to reduce his/hers level of stress acutely or if he/she experience chest pain. It is important the program is not perceived as a chore, which is emphasized to the patient.

The specific therapeutic elements of the program:

Biofeedback measuring of stress using the PPS measure as the measure of stress and to what extent the patient's heart is affected by stress followed by:

- Sensory nerve stimulation (acupressure/shiatsu massage)

The patients are instructed to perform the preventive nerve stimulation twice a day plus ad hoc when they urgently want to reduce their stress. Nerve stimulation is done by applying pressure with a finger for one minute – you should feel it, but it should not cause any pain, This is done at the following acupuncture points; on the sternum at the level of the fourth intercostals space (Shanzhong, C.V. 17), and on the back 3,8 cm lateral to the spinal process of the fourth and fifth thoracic vertebra (Jueyinshu and Xinshu, U.B. 14 and 15). Nerve stimulation is done successfully when the patient can observe that the tenderness of the point has been reduced after applying pressure. If this result has not been achieved, the patient is instructed to repeat the treatment. The repetitive nerve stimulation has two primary goals: 1) to restore the nervous system's ability to adapt through the repetitive stimulation of the diffuse pain inhibitory control (DNIC) system (Arendt-Nielsen L and Yarnitskyy D, 2010) and 2) to reduce an elevated stress level through the repeated non-painful sensory stimulation of the polymodal nerve cell, causing a

release of the oxytocin hormone (Uvnãs-Moberg, 1997a). If possible, the spouse or cohabitant will be instructed to perform nerve stimulation on the patient's back, which may independently contribute to stress-relieving effects through a separate oxytocin release caused by the human care (Carter et al., 2007;Grewen et al., 2005;Light et al., 2005;Uvnãs-Moberg, 1997b). If a spouse or cohabitant is not available, the patient can perform the treatment himself with a ball (the person will receive instructions in the technique).

9.2.3 Joint intervention for both the active and control group:

All participating patients receive a book on general stress management ("Stress, Lægens Bord" Netterstrøm, Bo, DR, 2002).

9.3 Blood sampling and analysis:

Blood samples are taken locally at Herlev Hospital where they are stored at -80 degrees until analysis.

After analysis, and by June 1st 2013, the debris is destroyed.

All blood tests will be performed at Medical Department O, Endocrinology Unit, Research Lab 54O4, Herlev Hospital, either as a routine or as a research analysis through commercial analysis-kits. All samples are analyzed in the same assay to avoid inter-assay variation, i.e. the analyses are first performed after completion of the study.

The following is analyzed (baseline study and intervention study combined):

Measure of persistent stress: HbA1c, serum Oxytocin.

Measure of insulin resistance: Fasting serum / plasma insulin, C-peptide, blood glucose (with calculation of HOMA), HbA1c, Adiponectin.

Measure of chronic inflammation: Serum / plasma high sensitive CRP, Defensin, YKL-40.

Measure of fat metabolism: total cholesterol, HDL and LDL cholesterol and Triglycerides.

Measure of cardiac strain: NT-proBNP and BNP.

Besides this, a spare glass of 5 ml of blood is taken for any later use at baseline and

at the 3 month follow-up. These spare glasses are stored in coded form (without CPR number) in one of the Danish Data Protection Agency and the Capital Region's approved bio banks (database id: 30122009.HEH.O.JF). This database is an ongoing database without a planned end date.

9.4 Body composition:

The body composition is done using whole-body DEXA scan (apparatus: Hologic) for the determination of body composition in fat, muscle and bone mass. Furthermore, the visceral

fat is calculated using new software to distinguish between android and gynoid fat. A well- calibrated x-ray is used for the scanning of the patient.

9.5 HRV post test:

The subjects get their short-term HRV (5 minutes) measured by an EKG (heart electro diagram) in supine rest. The measurement is done with the participant lying on a folding bed. After the rest, the participant is tilted without using his or her own muscle power to a 70 degrees (upright) position. EKG is registered in this position. The difference between resting and standing represents the reactivity of the autonomic nervous system. Lower reactivity in the autonomic nervous system is associated with persistent stress. The measurement of HRV is non-invasive and is not associated with discomfort or any risk for the participants.

9.6 Algometry on the Tibia bone with an established Algometer

Pain threshold will be measured at the Tibia bone with both the PPS measure device used in this study as well as with a known and established algometer (Arndt-Nielsen et al. 2010). Fifty randomly selected patients will be studied during their follow-up visit after 3 months.

10. Time frame for the study

- The first patient is expected to begin 3 months after the financial resources has been provided, and after approval by the Scientific Ethics Committee and the Danish Data Protection Agency.

- The inclusion period ends when the desired number of included patients is reached (300).
- The treatment period ends three months after the last patient is included.
- The scientific main article is expected to be complete after 3 additional months.
- The total project period is estimated as one year from the inclusion of the first patient.

11. Statistics

11.1 Power and calculation of the size of the study population

The primary analysis is based on the depression score of the MDI questionnaire. The prevalence of depression measured as a MDI score ≥ 20 was found to be approximately 20 % in patients with ischemic heart disease (Olsen et al 2003; Bech P 2001). In a study with 292 office workers, the prevalence of a MDI score ≥ 20 was twice as high in people with a PPS measure ≥ 60 compared to people with PPS < 40 (Ballegaard et al 2011). Against this background, it is estimated that among patients with ischemic heart disease who at the same time have an elevated PPS measure the prevalence of an MDI ≥ 20 is increased by 80 % compared to patients with ischemic heart disease as a whole, i.e. 36 %.

The Minimal Important Difference (MID) is set at 0.4 when the effect of active treatment is compared to control treatment. MID in terms of improvement of a MDI score is set at 5 points on the MDI scale when the active and control groups are compared. The standard deviation of improvement in both groups is set at 9 units on the MDI scale. Alfa is set at 5 %, Beta at 20 %. Against this background, 50 patients with an MDI score ≥ 20 are required in each group, thus 300 patients in total when safety margins of 10 % of the estimated values are included.

An interim analysis on the first 60 patients included showed, that the anticipated prevalences for MDI score were different than found. Therefore the cutpoint on the MDI scale was changed to MDI score = 15.

- Effect size is calculated from all included patients (on intention to treat basis)
- Effect size is a fraction that consists of the difference in a MDI score from before intervention till after treatment for the active group, minus the same

difference for the control group divided by the spread of the change in MDI score, when taking all patients together. In a review of all of the 12 SSRI preparations that the FDA has approved for treatment of depression, the average effect size is 0.3 (Turner et al., 2008). Example: the MDI score in active group changes from 24 to 11 (24-11) = 13; while the MDI score for the control group changes from 24 to 14 (24-14) = 10. This change in the MDI score is 13-10 = 3; if the spread (standard deviation) on the MDI score overall are 7.5, the effect size will be 3/7.5 = 0,4 (Per Bech, 2010).

- The Minimal Important Difference (MID) in effect size for the WHO-5 questionnaire is calculated similarly to 0,5 (Per Bech, 2010).

11.2 Analysis of the effect variables

Five percent (5 %) will be used as significance level in all statistic tests.
The statistical analysis will look at two groups of cardiac patients separately: 1) those patients for whom all therapeutic approaches have been exhausted and 2) the rest. One joint analysis will be made if it is statistically reasonable. The statistics are compiled on an ”intention to treat basis”; meaning that all enrolled patients are included in the subsequent analysis.
In the statistical analysis, subgroup analysis will be made for the patients with the highest PPS values: e.g. all patients with PPS ≥ 75; or for the highest quartile of PPS values.

“Closed test procedure” will be used to analyze data after 3 and 12 months in terms of the assessment of the treatment effect:

- First priority hypothesis: The Ull Care program improves the degree of depression measured by a MDI depression score, measured after 3 months of intervention.
- Second priority hypothesis: The Ull Care program improves health economic variables, measured after 12 months of intervention.
- Third priority hypothesis: The Ull Care program improves the frequency of clinical events, measured after 12 months of intervention.

Baseline measurements will be co-variant variables in relation to the applied stratification variable: the degree of depression measured by the MDI scale. Data from before and after the intervention will be analyzed separately. Delta values for

the active and control group respectively will be compared to analyze a possible treatment effect. All secondary and tertiary effect variables will be analyzed after the intervention through variance and regression analysis. If the premises for the proposed statistical analysis cannot be met, non- parametric analysis is used. Quality-of-life data will be analyzed to meet the directions of each of the questionnaires. All effect data will be presented a unifying table and primary, secondary and tertiary effect variables will be listed.

11.3. Cost-Utility analysis (CUA)

A cost-utility analysis will be carried through after 12 months of intervention for comparison of active versus control treatment. Relevant data, meaning consumption of healthcare services such as hospital visits, ambulatory visits, use of medication, consumption of invasive procedures etc. will be gathered during the study. Data from SF-36 questionnaires will be used to generate OALY's based on the fact that the Minimal Important Difference (MID) in effect on physical health (PCS scale) and mental health (MCS scale) is 0,3 (calculated as shown in point 11.1) (Ware JE et al 2007). Estimations of indirect costs, such as social pensions will be included. CUA will be calculated both with and without the indirect costs.

11.4. Additional studies

It is the intention to repeat the Songheart study with an identical design in Israel with the purpose of subsequently being able to add data from the two studies together and thus increase the overall statistical power.

11.5 Analysis of substudies:

Separate analyses will be performed with regard to:

The effect of intervention in the following subgroups:

a. Age (<65, ≥65)
b. Gender
c. Heart failure (yes,no)
d. Diabetes (yes,no)
e. Time since last MI or invasive procedure: <2 yr, ≥2 yr.

12. Success criteria

The result of the study will be concluded as positive, if the following results are reached:

Compared to the control group, the active group obtains a significant improvement in MDI depression score and with a therapeutic effect size of at least 0,4 when the effect in the active group is compared to the control group (see point 11.1 for exact calculation of the effect size).

When compared to the control group, the active group obtains a significant improvement in mean PPS measurement measured by a professional instructor and with a MID of 10 units on the PPS scale.

The patient does not experience any increased risk in connection with the intervention measured as no increase in blood pressure or serum cholesterol after treatment, Both parts are measured as mean values for the two variables in the active group when the baseline values are compared at the end of the treatment period.

13. Usefulness of the study and treatment in a broader perspective

By participating in the study, each patient can get a general profit from the opportunity of an additional comprehensive health check. If the findings of the study are positive, it means that the patients in the active group have an extra benefit in terms of reduced stress, improved mood and some concrete tools for better stress management. If renewed fund applications allow it, the control group will then be offered the active treatment. The study gives the surrounding society the possibility of testing the value of a new and economically inexpensive treatment in heart patients: A treatment that is expected to have a measurable impact on the patients' future health, use of health care services and patient survival. Other scientific studies have shown that if stress and depression is reduced in a heart patient, their survival is improved. As the intervention represents a paradigm shift in the treatment of chronic illness with an emphasis on a far greater self-insight and self-effort, a positive outcome will expectedly lead to a test of the intervention in people with other chronic illnesses. The study is also designed to uncover possible links between persistent stress and heart disease and which mechanisms that is responsible for the effect that a positive outcome will show.

14. Ethics

The study depends on an approval from the Scientific Ethics Committee. After approval, the study will be reported to www.clinicaltrials.gov. The study is also reported to the Danish Data Protection Agency. The study complies with the Helsinki Declaration.

The study will meet Danish medical guidelines of good clinical practice.

All subjects are informed orally and in writing about the study. The investigator will obtain informed written consent. All subjects receive the booklet ”Your rights as a test subject in a biomedical research project”, written ”Participant information” and an offer of a personal oral information session in the initial letter with an invitation for participation.

There will be emphasis on the fact that the information session is about a query of participation, and that the subject has the right to bring an accompanying person. The subject’s right not to receive information on his/hers health condition and disposition to disease will be reviewed and respected.

If during the conduct of the trial new information on effect, risks and side effects appear, the subjects will be informed and a new informed consent may be obtained.

If the subject gives written permission, a spare blood sample glass (5 ml) will be stored from both baseline and the month follow-up in one of the Danish Data Protection Agency’s approved bio banks (jr.nr 2005-41-5655, later 2008-41-2031, and from Dec 2009: 30122009.HEH.O.JF). These samples are stored in coded form (i.e. without CPR number) and indefinitely.

Use of the blood samples for any other purpose than what the subjects initially consented and what is referred to in this protocol will follow the usual guidelines, including a renewed reporting to the ethics committee.

Approximately 40 ml of blood per time (two times total) is taken. This includes the spare blood sample glass.

There will be no financial compensation to subjects.

15. Risks and rights of the participants

The patients are not deprived of other known effective treatment that are offered to similar patients at hospitals in Copenhagen, and participation in the study does not interfere with the patient's current treatment.
Blood samples are done through vein puncture and will be performed with standard equipment, which leads to a known and limited discomfort.

The DEXA scan uses low x-rays. The radiation dose is approximately 0,1 mSv per examination, which is the same as approximately 3 % of the natural background radiation every year.
By measuring the pressure threshold, some soreness or bruises may occur if the participant does not follow the instructions and says stop or stops in time.
The HRV position testing is non-invasive and without discomfort or risk to the person. The given active intervention is risk-free. Both control and active group receive a book on general stress management.
The subjects are informed that they have the opportunity to complain to the Patient Complain Board and seek compensation through the Patient Insurance Association.

16. Conflicts of interest

Søren Ballegaard is a shareholder in Ull Care A/S and is the inventor of the Ull Meter. To minimize possible bias, he shall not participate in 1) patient selection, 2) patient instruction and contact (he well only supervise the treatment performed by others), 3) evaluation of the treatment
The responsible researcher is Professor Jens Faber, Medical Department O, Herlev Hospital, and he is associated with Ull Care A/S as an independent, unpaid and external scientific advisor. Jens Faber does not own shares or other financial interests in Ull Care A/S and has never received funding/fees from the company.
No other participants are associated with Ull Care A/S and nobody has any economic benefit form the study.

17. Publications

Both positive and negative results will be compiled in one or more scientific articles

and sent to international peer-reviewed publications regarding publication. The steering group will be co-authors of all the scientific articles. Affiliated researchers will be co-authors on the articles where they have contributed.

18. Use of data and notification of the Danish Data Protection Agency

All data rights regarding scientific evaluation and publication are owned by the project responsible researcher, and the project is reported to the Danish Data Protection Agency after the approved guidelines of Herlev Hospital.
Raw data will in anonymized form and after appointment with the principal investigator be accessible for all the affiliated institutions for use in research projects. All publications must be confirmed by the primarily responsible researcher Jens Faber.

19. Economy

The study seeks funding by both private and public funds. The total budget is 3.5 million DKK.

20. Organization and responsibility

Jens Faber is the principal investigator.
The steering group consists of Per Bech, Åke Hjalmarson and Søren Ballegaard. Søren Ballegaard is included in the steering committee because of his special competence in relation to the active intervention included in the study. Jens Faber is an adjunct member of the steering group and therefore has no voting rights.

21. References

Alonso, J. et al., 2004, Health-related quality of life associated with chronic conditions in eight countries: results from the International Quality of Life Assessment (IQOLA) Project: Qual.Life Res., v. 13, no. 2, p. 283-298.

Arendt-Nielsen L, and Yarnitskyy D, 2010, Experimental and Clinical Applications of Quantitative Sensory Testing Applied to Skin, Muscles and Viscera: J of Pain.

Axelsson, A., P. Schousen, B. Karpatschoff, J. Nyboe, and S. Ballegaard, 2011, Acupuncture and Breast Cancer: A pilot study.: submitted for publication.

Ballegaard, S., E. Borg, B. Karpatschof, J. Nyboe, and A. Johannessen, 2004, Long-term effects of integrated rehabilitation in patients with advanced angina pectoris: a nonrandomized comparative study: J Altern.Complement Med, v. 10, no. 5, p. 777-783.

Ballegaard, S., A. Johannessen, B. Karpatschof, and J. Nyboe, 1999, Addition of acupuncture and self-care education in the treatment of patients with severe angina pectoris may be cost beneficial: an open, prospective study: J Altern.Complement Med, v. 5, no. 5, p. 405-413.

Ballegaard, S., B. Karpatschof, W. Trojaborg, A. M. Hansen, G. Magnusson, and P. B. Petersen, 2009, A simple and objective marker for stress: Scand J Clin.Lab Invest, v. 69, no. 6, p. 713-721.

Ballegaard, S., S. Norrelund, and D. F. Smith, 1996, Cost-benefit of combined use of acupuncture, Shiatsu and lifestyle adjustment for treatment of patients with severe angina pectoris: Acupunct.Electrother.Res., v. 21, no. 3-4, p. 187-197.

Ballegaard, S., P. B. Petersen, F. Gyntelberg, and J. Faber, 2011, Validation of a new stress marker in Danish office workers: A feasibility study: submitted for publication

Bech, P., 2010, Klinisk psykometri: København, Munkgaards forlag, p. 69-72.

Bech, P., N. A. Rasmussen, L. R. Olsen, V. Noerholm, and W. Abildgaard, 2001, The sensitivity and specificity of the Major Depression Inventory, using the Present State Examination as the index of diagnostic validity: J Affect.Disord., v. 66, no. 2-3, p. 159-164.

Bibbins-Domingo, K., G. M. Chertow, P. G. Coxson, A. Moran, J. M. Lightwood, M. J. Pletcher, and L. Goldman, 2010, Projected effect of dietary salt reductions on future cardiovascular disease: N.Engl.J Med, v. 362, no. 7, p. 590-599.

Birket-Smith, M., B. H. Hansen, J. A. Hanash, J. F. Hansen, and A. Rasmussen, 2009, Mental disorders and general well-being in cardiology outpatients--6-year survival: J.Psychosom.Res., v. 67, no. 1, p. 5-10.

Bjorner, J. B., K. Thunedborg, T. S. Kristensen, J. Modvig, and P. Bech, 1998, The

Danish SF-36 Health Survey: translation and preliminary validity studies: J Clin.Epidemiol., v. 51, no. 11, p. 991-999.

Bronnum-Hansen, H., M. Davidsen, and P. Thorvaldsen, 2001, Long-term survival and causes of death after stroke: Stroke, v. 32, no. 9, p. 2131-2136.

Carter, C. S., H. Pournajafi-Nazarloo, K. M. Kramer, T. E. Ziegler, R. White-Traut, D. Bello, and D. Schwertz, 2007, Oxytocin: behavioral associations and potential as a salivary biomarker: Ann N Y Acad Sci, v. 1098, p. 312-322.

Dansk Cardiologisk Selskab, 2010, Hjerterehabilitering, in Dansk Cardiologisk Selskab ed., National Behandlingsvejledning 2010.

De, B. G. et al., 2003, European guidelines on cardiovascular disease and prevention in clinical practice: Atherosclerosis, v. 171, no. 1, p. 145-155.

Del Pozo, J. M., R. N. Gevirtz, B. Scher, and E. Guarneri, 2004, Biofeedback treatment increases heart rate variability in patients with known coronary artery disease: Am.Heart J, v. 147, no. 3, p. E11.

Dempster, M., and M. Donnelly, 2000a, Measuring the health related quality of life of people with ischaemic heart disease: Heart, v. 83, no. 6, p. 641-644.

Dimsdale, J. E., 2008, Psychological stress and cardiovascular disease: J Am.Coll.Cardiol., v. 51, no. 13, p. 1237-1246.

Ekman, R., and G. Lindstedt, 2002, Molekyler på liv og død, in R Ekman and B Arnetz eds., Stress; Molekylerne, Individen, Organisationen, Samhället: Sverige, Liber Press, p. 69-89.

Elhendy, A., K. M. Modesto, D. W. Mahoney, B. K. Khandheria, J. B. Seward, and P. A. Pellikka, 2003, Prediction of mortality in patients with left ventricular hypertrophy by clinical, exercise stress, and echocardiographic data: J Am.Coll.Cardiol., v. 41, no. 1, p. 129- 135.

Farmer, J. A., 2004, Hypertension and the metabolic syndrome: Curr.Cardiol.Rep., v. 6, no. 6, p. 427-433.

Folker, H., and A. P. Folker, 2008, [WHO-5 as a simple method for measuring quality of life in daily psychiatric clinics]: Ugeskr.Laeger, v. 170, no. 10, p. 830-834.

Ford, E. S., 2005, Prevalence of the metabolic syndrome defined by the International Diabetes Federation among adults in the U.S: Diabetes Care, v. 28, no. 11, p. 2745-2749.

Grewen, K. M., S. S. Girdler, J. Amico, and K. C. Light, 2005, Effects of partner support on resting oxytocin, cortisol, norepinephrine, and blood pressure before and after warm partner contact: Psychosom.Med., v. 67, no. 4, p. 531-538.

Grippo, A. J., and A. K. Johnson, 2009, Stress, depression and cardiovascular dysregulation: a review of neurobiological mechanisms and the integration of

research from preclinical disease models: Stress., v. 12, no. 1, p. 1-21.

Gulliksson, M., G. Burell, B. Vessby, L. Lundin, H. Toss, and K. Svardsudd, 2011, Randomized controlled trial of cognitive behavioral therapy vs standard treatment to prevent recurrent cardiovascular events in patients with coronary heart disease: Secondary Prevention in Uppsala Primary Health Care project (SUPRIM): Arch.Intern.Med, v. 171, no. 2, p. 134- 140.

Harkonmaki, K., O. Rahkonen, P. Martikainen, K. Silventoinen, and E. Lahelma, 2006, Associations of SF-36 mental health functioning and work and family related factors with intentions to retire early among employees: Occup Environ.Med, v. 63, no. 8, p. 558-563.

Holmes, S. D., D. S. Krantz, H. Rogers, J. Gottdiener, and R. J. Contrada, 2006a, Mental stress and coronary artery disease: a multidisciplinary guide: Prog.Cardiovasc.Dis., v. 49, no. 2, p. 106-122.

Jain, D., T. Joska, F. A. Lee, M. Burg, R. Lampert, and B. L. Zaret, 2001, Day-to-day reproducibility of mental stress-induced abnormal left ventricular function response in patients with coronary artery disease and its relationship to autonomic activation: J Nucl.Cardiol., v. 8, no. 3, p. 347-355.

Koschke, M., M. K. Boettger, S. Schulz, S. Berger, J. Terhaar, A. Voss, V. K. Yeragani, and K. J. Bar, 2009, Autonomy of autonomic dysfunction in major depression: Psychosom.Med., v. 71, no. 8, p. 852-860.

Kristen, A. V. et al., 2010, Acupuncture improves exercise tolerance of patients with heart failure: a placebo-controlled pilot study: Heart, v. 96, no. 17, p. 1396-1400.

Kumazawa, T., 1998a, Primitivism and plasticity of pain--implication of polymodal receptors: Neurosci.Res., v. 32, no. 1, p. 9-31.

Lanza, G. A., K. Fox, and F. Crea, 2006, Heart rate: a risk factor for cardiac diseases and outcomes? Pathophysiology of cardiac diseases and the potential role of heart rate slowing: Adv.Cardiol., v. 43, p. 1-16.

Lewington, S., G. Whithlock, R. Clarke, P. Sherliker, J. Emberson, J. Halsey, N. Qizilbash, R. Peto, and R. Collins, 2007, Blood cholesterol and vascular mortality by age, sex and bloodpressure: a meta-analysis of individual data from 61 prospective studies with 55000 vascular deaths: Lancet, v. 370, p. 1829-1839.

Lewington, S., R. Clarke, N. Qizilbash, R. Peto, and R. Collins, 2002, Age-specific relevance of usual blood pressure to vascular mortality: a meta-analysis of individual data for one million adults in 61 prospective studies: Lancet, v. 360, no. 9349, p. 1903-1913.

Light, K. C., K. M. Grewen, and J. A. Amico, 2005, More frequent partner hugs and higher oxytocin levels are linked to lower blood pressure and heart rate in premenopausal women: Biol.Psychol., v. 69, no. 1, p. 5-21.

Magnusson, G., S. Ballegaard, B. Karpatschof, and J. Nyboe, 2010a, Long-term effects of integrated rehabilitation in patients with stroke: a nonrandomized comparative feasibility study: J Altern.Complement Med, v. 16, no. 4, p. 369-374.

Meagher, M. W., A. R. Ferguson, E. D. Crown, S. McLemore, T. E. King, A. N. Sieve, and J. W. Grau, 2001, Shock-induced hyperalgesia: IV. Generality: J Exp.Psychol Anim Behav.Process, v. 27, no. 3, p. 219-238.

Nilius, B., J. Vriens, J. Prenen, G. Droogmans, and T. Voets, 2004, TRPV4 calcium entry channel: a paradigm for gating diversity: Am.J Physiol Cell Physiol, v. 286, no. 2, p. C195- C205.

Nishiyama, S. et al., 2005, [Health-related quality of life in Japanese patients with ischemic heart disease: a multicenter cooperative investigation assessed using SF-36]: J.Cardiol., v. 46, no. 6, p. 211-220.

Noble, R. E., 2002, Diagnosis of stress: Metabolism, v. 51, no. 6 Suppl 1, p. 37-39.

Nolan, R. P., P. Jong, S. M. Barry-Bianchi, T. H. Tanaka, and J. S. Floras, 2008, Effects of drug, biobehavioral and exercise therapies on heart rate variability in coronary artery disease: a systematic review: Eur.J Cardiovasc.Prev.Rehabil., v. 15, no. 4, p. 386-396.

Oldridge, N., A. Perkins, and Z. Hodes, 2002, Comparison of three heart disease specific health-related quality of life instruments: Monaldi Arch.Chest Dis., v. 58, no. 1, p. 10-18.

Olsen, L. R., D. V. Jensen, V. Noerholm, K. Martiny, and P. Bech, 2003, The internal and external validity of the Major Depression Inventory in measuring severity of depressive states: Psychol.Med, v. 33, no. 2, p. 351-356.

Opie, L. H., 2001, Normal and abnormal cardiac function, in E Braunwald, Zippes D.P., and P Lippy eds., Heart Disease: W. B. Saunders, p. 468-469.

Orth-Gomer, K., N. Schneiderman, H. X. Wang, C. Walldin, M. Blom, and T. Jernberg, 2009, Stress reduction prolongs life in women with coronary disease: the Stockholm Women's Intervention Trial for Coronary Heart Disease (SWITCHD): Circ.Cardiovasc.Qual.Outcomes., v. 2, no. 1, p. 25-32.

Per Bech, 2010, Klinisk Psykometri Copenhagen, Munksgaard Danmark.

Pilcher, C. W., and J. L. Browne, 1983, Effects of naloxone and Mr 1452 on stress-induced changes in nociception of different stimuli in rats: Life Sci., v. 33 Suppl 1, p. 697-700.

Pocock, S. J., and R. Simon, 1975, Sequential treatment assignment with balancing for prognostic factors in the controlled clinical trial: Biometrics, v. 31, no. 1, p. 103-115.

Pradhan, A. D., and P. M. Ridker, 2002, Do atherosclerosis and type 2 diabetes share a common inflammatory basis?: Eur.Heart J., v. 23, no. 11, p. 831-834.

Radford, M. J., and J. M. Foody, 2001, How do observational studies expand the evidence base for therapy?: JAMA, v. 286, no. 10, p. 1228-1230.

Rogowski, O., I. Shapira, A. Shirom, S. Melamed, S. Toker, and S. Berliner, 2007, Heart rate and microinflammation in men: a relevant atherothrombotic link: Heart, v. 93, no. 8, p. 940- 944.

Routledge, F. S., T. S. Campbell, J. A. McFetridge-Durdle, and S. L. Bacon, 2010, Improvements in heart rate variability with exercise therapy: Can.J.Cardiol., v. 26, no. 6, p. 303-312.

Seematter, G., C. Binnert, J. L. Martin, and L. Tappy, 2004, Relationship between stress, inflammation and metabolism: Curr.Opin.Clin.Nutr.Metab Care, v. 7, no. 2, p. 169-173.

Sneddon, L. U., V. A. Braithwaite, and M. J. Gentle, 2003, Do fishes have nociceptors? Evidence for the evolution of a vertebrate sensory system: Proc.Biol.Sci., v. 270, no. 1520, p. 1115-1121.

Stansfeld, S. A., H. Bosma, H. Hemingway, and M. G. Marmot, 1998, Psychosocial work characteristics and social support as predictors of SF-36 health functioning: the Whitehall II study: Psychosom.Med, v. 60, no. 3, p. 247-255.

Surtees, P. G., N. W. Wainwright, R. N. Luben, N. J. Wareham, S. A. Bingham, and K. T. Khaw, 2008, Depression and ischemic heart disease mortality: evidence from the EPIC- Norfolk United Kingdom prospective cohort study: Am.J Psychiatry, v. 165, no. 4, p. 515- 523.

Szczepanska-Sadowska, E., A. Cudnoch-Jedrzejewska, M. Ufnal, and T. Zera, 2010, Brain and cardiovascular diseases: common neurogenic background of cardiovascular, metabolic and inflammatory diseases: J.Physiol Pharmacol., v. 61, no. 5, p. 509-521.

The National Board of Health,D. [Guidance on heart rehabilitation in hospitals]. 2004. Copenhagen, The National Board of Health, Denmark.
Ref Type: Pamphlet

The network group of "heart rehabilitation". [Heart rehabilitation in Danish hospitals]. 2004. Ref Type: Pamphlet

Timio, M., G. Lippi, S. Venanzi, S. Gentili, G. Quintaliani, C. Verdura, C. Monarca, P. Saronio, and F. Timio, 1997, Blood pressure trend and cardiovascular events in nuns in a secluded order: a 30-year follow-up study: Blood Press, v. 6, no. 2, p. 81-87.

Turner, E. H., A. M. Matthews, E. Linardatos, R. A. Tell, and R. Rosenthal, 2008, Selective publication of antidepressant trials and its influence on apparent efficacy: N.Engl.J.Med., v. 358, no. 3, p. 252-260.

Uvnãs-Moberg, K., 1997a, Oxytocin linked antistress effects - the relaxation and growth response: Acta Psychologica Scandinavica Supplementum, p. 38-42.

Uvnãs-Moberg, K., 1997b, Physiological and endocrine effects of social contact: Ann N Y Acad Sci., v. 807, p. 146-163.

Villella, M., A. Villella, S. Barlera, M. G. Franzosi, and A. P. Maggioni, 1999, Prognostic significance of double product and inadequate double product response to maximal symptom- limited exercise stress testing after myocardial infarction in 6296 patients treated with thrombolytic agents. GISSI-2 Investigators. Grupo Italiano per lo Studio della Sopravvivenza nell-Infarto Miocardico: Am.Heart J, v. 137, no. 3, p. 443-452.
